# Comparative assessment of immune evasion mechanisms in human whole-blood infection assays by a systems biology approach

**DOI:** 10.1371/journal.pone.0249372

**Published:** 2021-04-01

**Authors:** Teresa Lehnert, Maria T. E. Prauße, Kerstin Hünniger, Jan-Philipp Praetorius, Oliver Kurzai, Marc Thilo Figge

**Affiliations:** 1 Applied Systems Biology, Leibniz Institute for Natural Product Research Infection Biology, Hans Knöll Institute (HKI), Jena, Germany; 2 Center for Sepsis Control and Care (CSCC), Jena University Hospital, Jena, Germany; 3 Institute of Microbiology, Faculty of Biological Sciences, Friedrich Schiller University Jena, Jena, Germany; 4 Fungal Septomics, Leibniz Institute for Natural Product Research Infection Biology, Hans Knöll Institute (HKI), Jena, Germany; 5 Institute of Hygiene and Microbiology, University of Würzburg, Würzburg, Germany; University of the Witwatersrand, SOUTH AFRICA

## Abstract

Computer simulations of mathematical models open up the possibility of assessing hypotheses generated by experiments on pathogen immune evasion in human whole-blood infection assays. We apply an interdisciplinary systems biology approach in which virtual infection models implemented for the dissection of specific immune mechanisms are combined with experimental studies to validate or falsify the respective hypotheses. Focusing on the assessment of mechanisms that enable pathogens to evade the immune response in the early time course of a whole-blood infection, the least-square error (LSE) as a measure for the quantitative agreement between the theoretical and experimental kinetics is combined with the Akaike information criterion (AIC) as a measure for the model quality depending on its complexity. In particular, we compare mathematical models with three different types of pathogen immune evasion as well as all their combinations: (i) spontaneous immune evasion, (ii) evasion mediated by immune cells, and (iii) pre-existence of an immune-evasive pathogen subpopulation. For example, by testing theoretical predictions in subsequent imaging experiments, we demonstrate that the simple hypothesis of having a subpopulation of pre-existing immune-evasive pathogens can be ruled out. Furthermore, in this study we extend our previous whole-blood infection assays for the two fungal pathogens *Candida albicans* and *C*. *glabrata* by the bacterial pathogen *Staphylococcus aureus* and calibrated the model predictions to the time-resolved experimental data for each pathogen. Our quantitative assessment generally reveals that models with a lower number of parameters are not only scored with better AIC values, but also exhibit lower values for the LSE. Furthermore, we describe in detail model-specific and pathogen-specific patterns in the kinetics of cell populations that may be measured in future experiments to distinguish and pinpoint the underlying immune mechanisms.

## Introduction

As integral part of the systems biology cycle, mathematical models based on experimental measurements allow unraveling the complex network of immune reactions during infections. Representing the immune response by a mechanistic network, mathematical models have the strength to not only quantify immune reactions that are not directly accessible in experiments but also to predict the expected outcome of novel immune mechanisms. Moreover, the possibility to easily change model parameters or even the structure of a model enables making concrete predictions under well-defined conditions. Thereby, mathematical models help to guide future experimental measurements and by that can drastically reduce the financial and time expenditure.

In previous studies, we applied the iterative cycle between biological experiments and virtual infection modeling to unravel the immune response to fungal pathogens in human whole-blood infection assays [1–3]. The combination of experimental investigations with mechanistic modeling facilitated, on the one hand, the quantification of functional characteristics of the immune defense by specific immune cells and promoted, on the other hand, the identification of a fungal population that can be neither phagocytosed nor killed but instead evades the immune response. Virtual infection modeling was realized in terms of a state-based model (SBM), where the states are occupied by the populations of essential immune cells, *i*.*e*. polymorphonuclear neutrophils (PMNs) and monocytes, as well as pathogens that can be either alive or killed and that are located either in extracellular space or within the immune cells. The biological processes that take place during infection are implemented by state transitions, which are characterized by transition rates. In these SBMs, we defined rates for transitions representing phagocytosis of pathogens by PMNs and monocytes, intracellular and extracellular killing of pathogens as well as the acquisition of the immune-evasive state. We found that the SBM does not only mimic the infection dynamics in human whole-blood assays but also enables quantitative predictions of treatment strategies against fungal pathogens for neutropenic patients [4].

In our initial SBM the mechanism of becoming immune-evasive was implemented by a constant rate, because at that time there were no details known about this mechanism. Thus, we assumed that immune evasion takes place spontaneously [1]. Therefore, we will refer to this model as *spon-IE model* in the following. Even though, we could not yet identify the exact mechanism causing immune evasion, we were able to reject potential theories based on our previous experimental observations. For example, we observed that adding fresh blood from the same donor to an infected blood sample did not lead to elimination of the immune-evasive pathogens [1]. Consequently, immune evasion is not a result of exhausted immune cells. Additionally, we observed that immune-evasive *C*. *albicans* cells are still measureable when performing the whole-blood infection assays with thimerosal-killed *C*. *albicans* or with the non-filamentous *efg*1Δ, *cph*1Δ mutant of *C*. *albicans*. Thus, we could exclude that *C*. *albicans* proliferation or any process that is actively induced by the *C*. *albicans* cells is responsible for their immune evasion. Based on these experimental observations, we theoretically investigated the possibility that immune evasion is mediated by the host. In the study by Prauße *et al*. [2], we exploited the strength of mathematical modeling and implemented a host-mediated mechanism in the SBM in order to make predictions by comparing various infection scenarios. More precisely, we hypothesized that the degranulation of PMNs in response to initial phagocytosis events does not only cause the release of antimicrobial peptides but also leads to secretion of effector molecules that induce immune evasion. Since this potential evasion mechanism is mediated by molecules that originate from PMNs, the respective SBM is denoted by *PMNmed-IE model* in the following. In addition to whole-blood infection with *C*. *albicans*, we comparatively analyzed the immune response during infection with another fungal pathogen, *Candida glabrata*. We previously studied these two fungal pathogens with regard to differences in the PMN response against them [5, 6]. However, while we could identify remarkable differences in the model predictions for various infection scenarios under neutropenia, we could neither accept nor reject the PMNmed-IE model nor the spon-IE model on the basis of the existing experimental data [2]. Therefore, generating and analyzing additional experimental data will be one of the essential steps towards the identification of the immune evasion mechanism.

**Fig 1:**
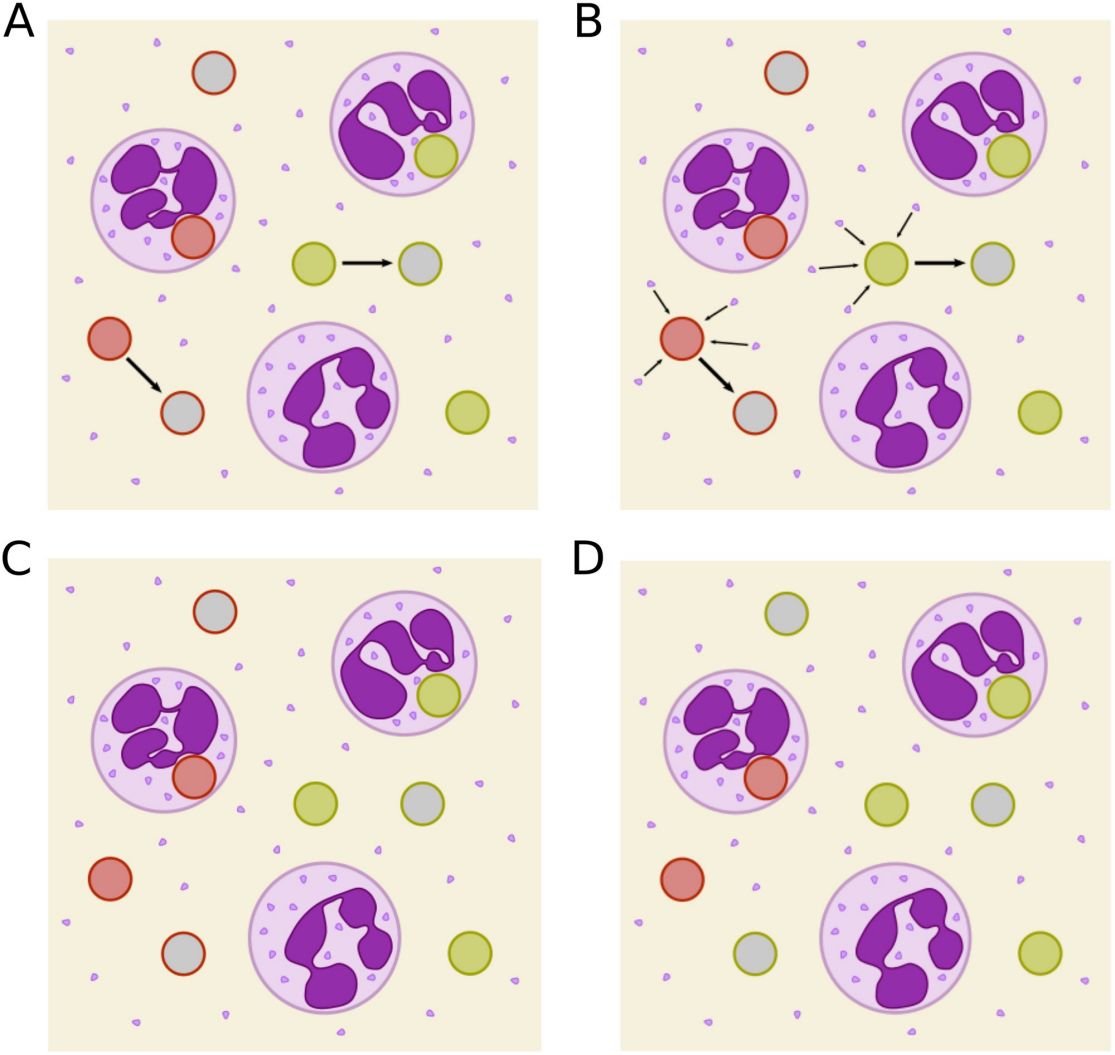
Schematic depictions of four immune-evasion mechanisms. PMN (purple) containing granules and pathogens that are either alive (green), killed (red), or immune-evasive (gray). Immune-evasive pathogens are either alive (green outer line) or killed (red outer line). (A) In the spontaneous immune evasion model (spon-IE model) pathogens acquire immune evasion with constant rate ρ. (B) PMN-mediated acquisition of immune evasion is mediated by factors released from neutrophils (PMNmed-IE model) that were secreted by PMNs in response to the first phagocytosis of pathogens with rate ρ(t). (C) In the pre-existing immune-evasion model (pre-IE model), immune-evasive properties have been acquired before infection. The alive as well as the killed subpopulation of immune-evasive cells is estimated by this model. (D) Schematics of the alivePre-existing immune-evasion model (alivePre-IE model). The difference to the pre-IE model is that in the alivePre-IE model only the population of alive immune-evasive cells is estimated and there is no possibility for killed immune-evasive cells to develop.

In the present study, we expand our search for the mechanism of immune evasion by performing a broader theoretical study involving further potential immune-evasion mechanisms and combine the corresponding models with the analysis of additional infection scenarios. According to the iterative cycle of systems biology, the resulting model predictions were partly tested experimentally within the scope of this study and, thereby, one potential immune-evasion model was rejected. First, we considered the possibility that immune evasion is not developing during the host response, but takes place before infection of the whole-blood sample by pathogens. This was realized in the so-called *pre-IE model* by eliminating the rate for immune evasion and instead implementing constant cell populations of alive and killed immune-evasive pathogens that are present before infection. Based on the quantitative predictions made by this model, we performed experiments that clearly negate the presence of killed immune-evasive pathogens before infection, such that the pre-IE model could be rejected. Instead, we continued our study with the *alivePre-IE model*, where we assume that solely a population of alive and immune-evasive pathogens is present before infection. Consequently, we were left with three base models that each contains one way to acquire immune evasion (see Fig 1): the spon-IE model, the PMNmed-IE model and the alivePre-IE model. In addition to these base models, we took into consideration that more than one immune-evasion mechanism could act in parallel and implemented all combinations of the three base models. Taken together, we obtained eight model combinations (see Table 1) and calibrated these models to the time-resolved experimental data on the viability of pathogens and the association of pathogens to PMNs and monocytes that were measured during whole-blood infection assays.

**Table 1:** State-based models (SBMs) with different immune-evasion mechanisms.

models	Immune-evasion mechanisms			Immune-evasion parameters	total number of parameters
	spontaneous	PMN- mediated	pre-existing		
spon-IE	x			*ρ*	7
PMNmed-IE		x		$\overset{-}{\rho },\gamma_{IE}$	8
pre-IE			x	*P*_*KIE*_, *P*_*AIE*_	8
alivePre-IE			x	*P*_*AIE*_	7
spon- PMNmed-IE	x	x		$\rho ,\overset{-}{\rho },\gamma_{AIE}$	9
spon-alivePre-IE	x		x	*Ρ*, *P*_*AIE*_	8
PMNmed-alivePre-IE		x	x	$\overset{-}{\rho },\gamma_{IE},P_{AIE}$	9
spon-PMNmed-alivePre-IE	x	x	x	$\rho ,\overset{-}{\rho },\gamma_{IE},P_{AIE}$	10

For each model, the implemented immune-evasion mechanisms (indicated by a cross as table entry), the respective *a priori* unknown parameters that contribute to immune evasion and the total number of parameters are given. The spon-IE model represents a spontaneous immune-evasion mechanism by a constant rate *ρ*. The hypothesis on immune evasion mediated by PMN is implemented in the PMNmed-IE model and represented by a time-dependent rate *ρ*(*t*) that is determined by the two constants $\overset{-}{\rho }$ and *γ*_*IE*_ (see Eq 10). The populations of immune-evasive pathogens that are alive (*P*_*AIE*_) and killed (*P*_*KIE*_), and that are pre-existent at infection time t = 0 are estimated in the pre-IE model. The alivePre-IE model contains only the population of evasive pathogens that are alive (*P*_*AIE*_) before infection. The SBMs containing more than one evasion mechanisms (combined models) are composed of at least two base models.

In addition to infection scenarios with *C*. *albicans* or *C*. *glabrata* infection, we extended the present study by a third scenario with the bacterial pathogen *Staphylococcus aureus*. These three microbes are opportunistic pathogens that colonize the human body and can become pathogenic in patients with a compromised immune system. Therefore, these pathogens constitute a serious risk, not only of superficial infections but also of severe systemic infections like bloodstream infections. The *Candida* species *C*. *albicans* and *C*. *glabrata* together colonize more than 50% of the human population [7–9] and are the most frequent species causing fungemia [10]. The gram-positive bacterium *S*. *aureus* is the leading cause of bacteremia [11–13]. A nationwide surveillance study on bloodstream infections in US hospitals revealed that *S*. *aureus*, *C*. *albicans* and *C*. *glabrata* together account for up to 30% of all cases and moreover, the associated mortality rates are extremely high [10]. Thus, there is an urgent need to investigate how these pathogens manage to evade the immune response and can frequently cause bloodstream infections potentially leading to organ failure and death.

After calibrating the models of immune evasion to the experimental data, we evaluated the goodness of these models using different quantitative measures: as a measure for the quantitative agreement between the theoretical and experimental kinetics, we compute the least-square error (LSE) that is minimized during the parameter estimation procedure [2–4, 12]. The LSE evaluation is combined with the Akaike information criterion (AIC) as a measure for the model quality depending on its complexity in terms of model parameterization [13]. Our results reveal that, by performing a comparative analysis of the different evasion models for *C*. *albicans*, *C*. *glabrata* and *S*. *aureus* infection, we can identify relative differences between the model predictions, which in turn enables us to suggest experimental investigations by which predicted immune-evasion mechanisms can be rejected or confirmed.

## Results

In this study, we tested hypotheses on the mechanism of immune evasion of pathogens in human whole blood by implementing various potential candidate mechanisms and their combinations into virtual infection models. These comprise four base models with a single mechanism to acquire immune evasion (see Fig 1 and Table 1) and four models with at least two evasion mechanisms, also referred to as combined models (see Table 1). In order to evaluate the different models, we calibrated each model to the experimentally determined kinetics of whole-blood infection assays with one of the three different pathogens *C*. *albicans*, *C*. *glabrata* and *S*. *aureus*.

### *S*. *aureus* induces specific immune response pattern in whole-blood infection assays

**Fig 2:**
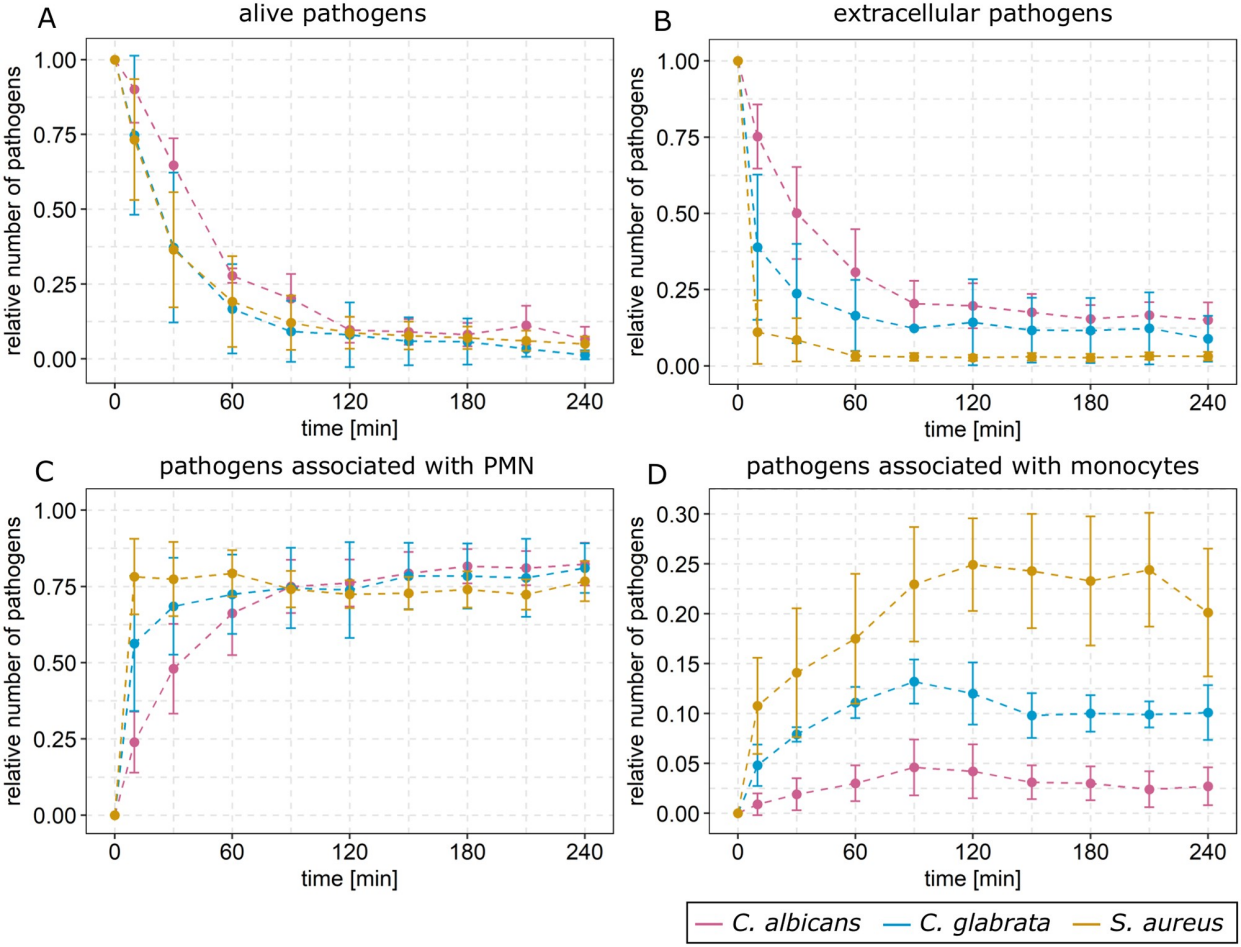
Experimental data acquired from whole-blood infection assays with *C*. *albicans*, *C*. *glabrata* and *S*. *aureus*. Experimental data from whole-blood infection assays infected with either *C*. *albicans* (pink line), *C*. *glabrata* (blue line) or *S*. *aureus* (orange line). Survival assays were performed to measure fractions of alive pathogens (A) and flow cytometry was used to determine the distribution of pathogens in human blood, shown as extracellular pathogens (B), pathogens associated with PMN (C) and with monocytes (D).

In our previous studies, we performed whole-blood infection assays with either *C*. *albicans* or *C*. *glabrata* and observed that infection outcomes strongly depend on the pathogen [1, 2]. In this study, we additionally performed whole-blood infection assays with the bacterial pathogen *S*. *aureus*. During the time course of four hours, we measured the viability of the pathogens by survival assays as well as the association of the pathogens to PMNs and monocytes using flow cytometry. The experimentally measured kinetics of pathogen association and viability are depicted in Fig 2 for *S*. *aureus* (orange lines) together with the kinetics from infection with *C*. *albicans* (pink lines) or *C*. *glabrata* (blue lines). Similar to *C*. *albicans* and *C*. *glabrata*, most *S*. *aureus* cells were killed in the whole-blood infection assay during four hours. A fraction of only 5.0% ± 2.4% alive *S*. *aureus* cells was measured four hours post infection (see Fig 2A). The time course of alive *S*. *aureus* cells in the whole-blood infection was qualitatively similar to that of alive *C*. *glabrata* cells, but in comparison to alive *C*. *albicans* cells a much faster dynamics was observed. The flow cytometry analysis revealed association of *S*. *aureus* cells to PMNs and monocytes, but no interaction with lymphocytes. Similar to infections with the fungal pathogens, association to immune cells increased with time and the majority of *S*. *aureus* cells was associated with PMNs (76.7% ± 6.5%), whereas a smaller fraction was associated with monocytes (20.1% ± 6.4%) at four hours post infection. However, the dynamics of *S*. *aureus* interaction with both immune cell types were faster compared to the two fungal pathogens, resulting in a smaller population of extracellular *S*. *aureus* cells after four hours of infection, and showed a markedly higher association to monocytes. (see Fig 2B).

Taken together, we found that, similar to the fungal pathogens, also *S*. *aureus* could not be cleared completely from human blood, which is a strong evidence for the existence of immune-evasive *S*. *aureus* cells at four hours post infection. Furthermore, we found that the immune response to *S*. *aureus* showed pathogen-specific properties, such as the greater impact of monocytes and the larger elimination of pathogens from whole blood in comparison to *C*. *albicans* and *C*. *glabrata*.

### Rejection of pre-IE model by image-based systems biology approach

Since we observed for all three types of pathogens that microbial cells can remain extracellular (see Fig 2B) and can remain alive, we investigated the hypothesis of a pre-existing immune-evasive phenotype in a proportion of the initial population used for infection (see Fig 1C). In the pre-IE model, we set the population of immune-evasive cells to a constant value that does not change over time (see Methods). This pre-existing subpopulation of immune-evasive cells can consist of alive and/or killed cells. In order to test whether this model captures the immune response as measured in the whole-blood infection assays with any of the three pathogens, we applied the parameter estimation algorithm Simulated Annealing based on the Metropolis Monte Carlo scheme (see S2 Fig of S1 File). This algorithm minimizes the least-square error (LSE); *i*.*e*. it determines the model parameter values for the best fit between model simulations and experimental data by minimizing the LSE. The resulting parameter values are given in S3 Table of S1 File. We observed that the corresponding model simulations matched the experimental data for all three infection scenarios. As shown in S10, S18 and S26 Figs of S1 File, respectively, for infection with *C*. *albicans*, *C*. *glabrata* and *S*. *aureus*, the model simulations were close to the mean values of the experimental data and within their corresponding standard deviations.

**Fig 3:**
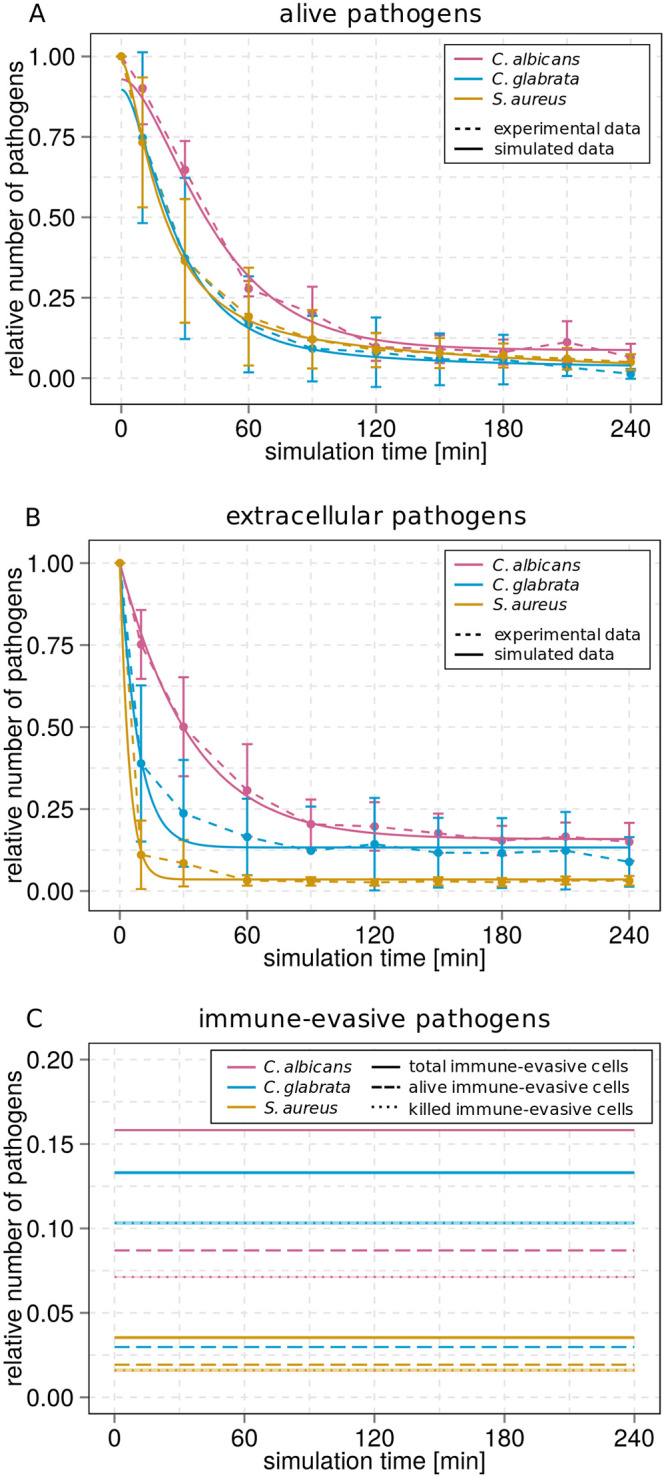
Kinetics of combined units for *C*. *albicans*, *C*. *glabrata* or *S*. *aureus* infection simulated by the pre-IE model. The thickness of the solid lines indicates the mean ± standard deviation (SD) of 50 simulations with transition rate values that were randomly sampled within their respective SD. Dashed lines correspond to the experimental data to which the model was fitted. The simulation results of the pre-IE model are depicted for an infection with *C*. *albicans* (pink line), *C*. *glabrata* (blue line) or *S*. *aureus* (green line). The time course of alive pathogens is shown at the top (A), the time course of extracellular pathogens in the middle (B) and the predicted time course total of immune-evasive (*P*_*IE*_), alive immune-evasive (*P*_*AIE*_) and killed immune-evasive cells (*P*_*KIE*_) is shown at the bottom (C).

In the following we focus on model simulations for extracellular pathogens and alive pathogens. For infection with *C*. *albicans* cells, the pre-IE model predicted that four hours post infection a fraction of 15.85% ± 0.0% pathogens was remaining in extracellular space (see Fig 3B) and that most of these cells were immune-evasive (15.81% ± 0.0%, see S10 Fig of S1 File). Furthermore, the model predicted that this fraction of evasive cells is composed of 7.1% ± 0.0% killed evasive cells and 8.7% ± 0.0% alive evasive cells. Thus, the model predicted that at the time point of infection (*t* = 0 *min*) a fraction of 7.1 ± 0.0% pathogens were already dead, *i*.*e*. not all pathogens were alive initially. This can be seen in Fig 3A, where the fraction of alive *C*. *albicans* cells was 92.9% ± 0.0% at *t* = 0 *min*. Similarly, the simulated dynamics of alive pathogens for infection with *C*. *glabrata* and *S*. *aureus*, respectively, started at *t* = 0 *min* with 10.3 ± 0.1% and 1.6 ± 0.01% killed evasive pathogens (see Fig 3C) and hence with 89.7% ± 0.1% and 98.4% ± 0.1% alive pathogens (see Fig 3A). Taken together, the pre-IE model predicted fractions of alive cells and killed cells that are immune-evasive before infection for all three infection scenarios.

So far, it is not possible to experimentally determine the fraction of immune-evasive cells before infection. However, we can test the model prediction by quantifying the amount of dead pathogens before infection using the live/dead staining with propidium iodide (PI) and subsequent microscopy imaging (see Methods). Example microscopy images for the three types of pathogens are shown in S27 Fig of S1 File. In order to count the number of alive and dead cells in these images, we applied the automated segmentation algorithm implemented in the framework AMIT (Algorithm for Migration and Interaction Tracking) [14], as described in Methods. In total, we analyzed six samples with *C*. *albicans* cells, six samples with C. *glabrata* cells and nine samples with *S*. *aureus* cells. Independent of the type of pathogen, we found that almost all cells in a viable pathogen inoculum were alive and that the number of dead pathogens was vanishingly small (see S28 Fig of S1 File): on average 0.0% ± 0.0% *C*. *albicans* cells, 0.2 ± 0.4% *C*. *glabrata* cells and 0.13% ± 0.07% *S*. *aureus* cells were dead before infection.

Taken together, for all three types of pathogens the experimentally determined fractions of dead pathogens before infection are vanishingly small and much smaller than the fractions of killed evasive pathogens that were predicted by the pre-IE model. These results strongly suggest that no dead pathogens are present at the beginning of the infection assays. On the other hand, since it cannot be excluded that alive evasive pathogens do exist before infection, we replace the pre-IE model by the alivePre-IE model. The latter model allows for the existence of an initial subpopulation of evasive pathogens that are all alive (see Methods). However, since dead cells do exist after four hours of infection (see Fig 2), the alivePre-IE model can only be considered in combination with other mechanisms of immune evasion. This conclusion motivated us to perform a comparative analysis of virtual infection models.

### Comparative analysis of virtual infection models for whole-blood assays

In order to find the most realistic immune-evasion model from the set of eight immune evasion models (see Table 1), we calibrated these models to the experimental data of all three types of pathogens using the global parameter estimation algorithm Simulated Annealing based on the Metropolis Monte Carlo scheme (see Methods section and S2 Fig of S1 File). Afterwards, we comparatively analyzed different model characteristics such as the estimated model parameter values and the goodness of the models, using the Akaike information criterion (AIC) in addition to the LSE. In the following paragraphs, we compare the predictions of the different immune-evasion models for each type of pathogen separately. Here, we first focus on the comparison of the alivePre-IE model with the other two base models that contain solely one way to acquire immune evasion, *i*.*e*. the spon-IE model and the PMNmed-IE model (see Fig 1A, 1B and 1D, Table 1 and Methods). Afterwards, we comparatively analyze the predictions made by the combined models, where three of them contain two evasion mechanisms (the spon-PMNmed-IE model, the spon-alivePre-IE model and the PMNmed-alivePre-IE model) and one model with all three potential evasion mechanisms, the spon-PMNmed-alivePre-IE model.

### Whole-blood infection with *C*. *albicans*

**Fig 4:**
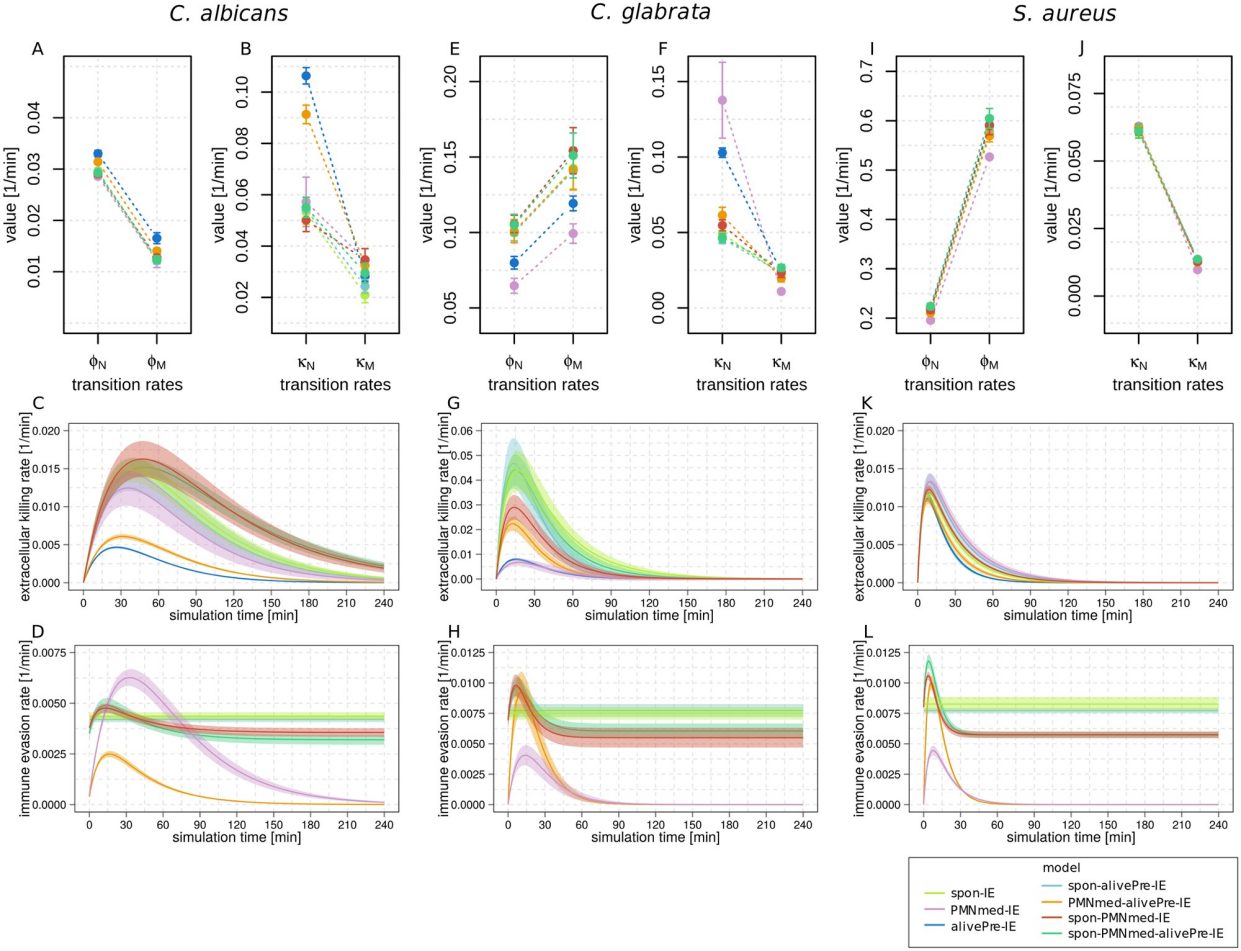
Resulting transition rate values from calibrating the potential immune evasion models to whole-blood infection scenarios with either *C*. *albicans*, *C*. *glabrata* or *S*. *aureus*. Mean values (data points) and standard deviations (error bars and line thickness) of transition rates were quantified by the global parameter estimation algorithm Simulated Annealing based. Dots depict the transition rate values for the spon-IE model (light green), PMNmed-IE model (pink), alivePre-IE model (dark blue), spon-alivePre-IE model (light blue), PMNmed-alivePre-IE model (orange), spon-PMNmed-IE model (red) and spon-PMNmed-alivPre-model (dark green). The transition rates for phagocytosis are defined by ***ϕ***_*N*_ for PMN and by *ϕ*_*M*_ for monocytes. Intracellular killing rates are defined by ***κ***_***N***_ for PMN and ***κ***_***M\***_ for monocytes. Transition rates ***γ*** and ${\overset{-}{\kappa }}_{EK}$ determine the time-dependent extracellular killing rate ***κ***_***EK***_(***t***). Depending on the chosen model, the transition rates ***ρ***, $\overset{-}{\rho }$ and *γ*_*IE*_ determine the immune-evasion rate over time (***ρ***(***t***)). Transition rate values for infection scenario with *C*. *albicans* for phagocytosis (A), intracellular killing (B), time course of the extracellular killing rate (C) and the time course of the immune-evasion rate (D). Transition rate values for infection scenario with *C*. *glabrata* infection for phagocytosis (E), intracellular killing (F), time course of the extracellular killing rate (G) and time course of the immune-evasion rate (H). Transition rate values for infection scenario with *S*. *aureus* infection for phagocytosis (I), intracellular killing (J), time course of the extracellular killing rate (K) and time course of the immune-evasion rate (L). Transition rates and start populations quantified for all base and combined models for each pathogen infection are also shown in S1-S3 Tables of S1 File.

**Fig 5:**
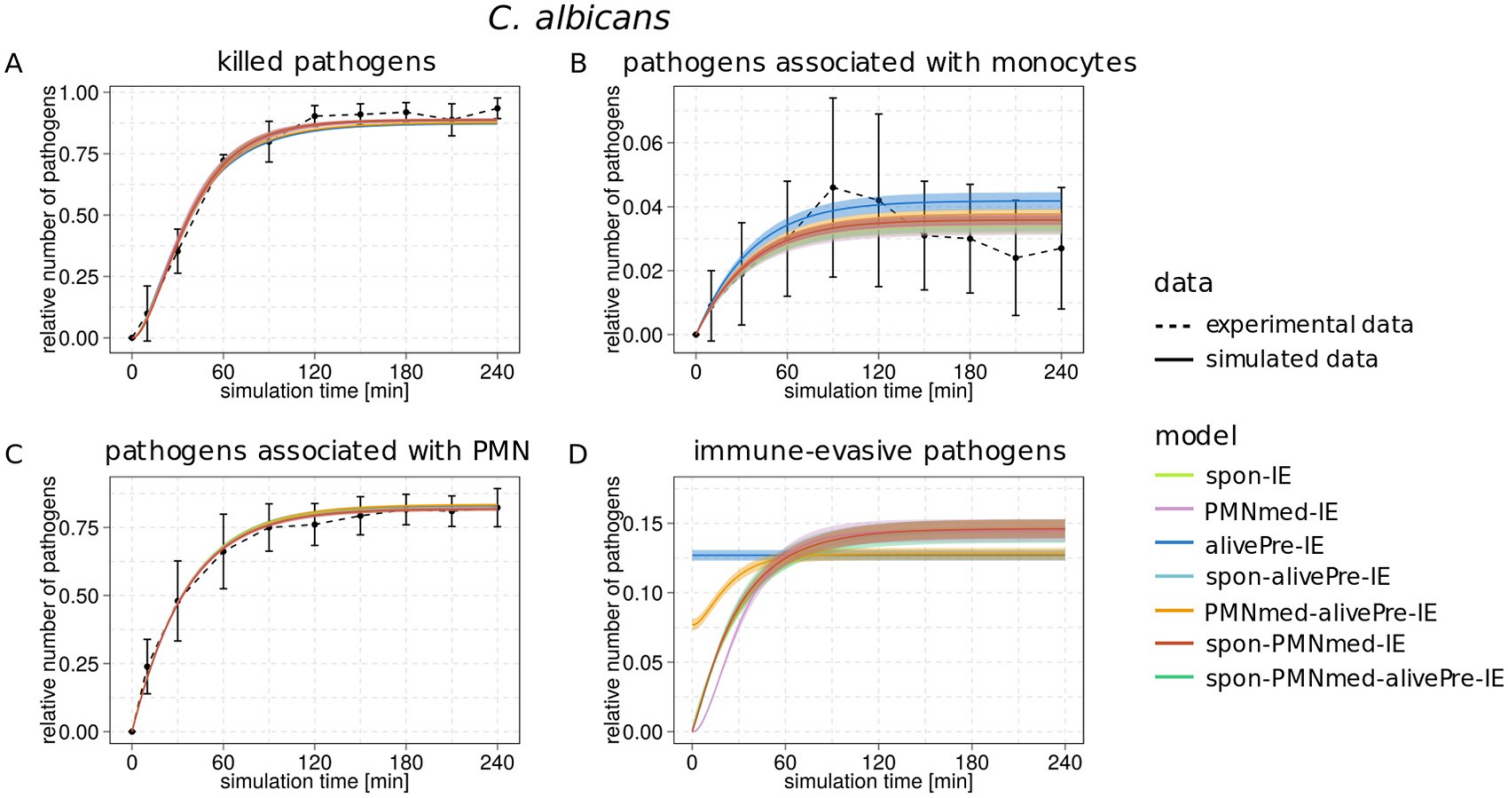
Kinetics of combined units of each immune-evasion model for infection scenario with *C*. *albicans*. Experimental data from whole-blood infection assays (dashed lines) with corresponding standard deviations (SDs) are compared to the simulated data (solid lines) for infection scenario with *C*. *albicans*. The simulated kinetics by the spon-IE model (light green line), PMNmed-IE model (pink line), alivePre-IE model (dark blue line), spon-alivePre-IE model (light blue line), PMNmed-alivePre-IE model (orange line), spon-PMNmed-IE model (red line) and spon-PMNmed-alivePre-IE model (dark green line) are shown in their corresponding color. The thickness of solid lines indicates the mean ± SD of 50 simulations with transition rate values that were randomly sampled within their respective SD. Simulated kinetics of killed cells (A), pathogens associated with monocytes (B), pathogens associated with PMN (C) and immune-evasive cells (D).

By fitting the immune-evasion models to data from whole-blood infection assays with the fungal pathogen *C*. *albicans* [1], we could estimate values of the model parameters and thereby quantify the immune reaction rates. The resulting parameter values are given in S1 Table of S1 File and depicted in Fig 4 for each immune-evasion model. We observed that the alivePre-IE model predicted larger phagocytosis rates of neutrophils (Φ_*N*_) and monocytes (Φ_*M*_) in comparison to the spon-IE model ($\frac{\Phi_{N}^{spon-IE}}{\Phi_{N}^{alivePre-IE}}=0.90$, $\frac{\Phi_{M}^{spon-IE}}{\Phi_{M}^{alivePre-IE}}=0.72$) and the PMNmed-IE model ($\frac{\Phi_{N}^{PMNmed-IE}}{\Phi_{N}^{alivePre-IE}}=0.87$, $\frac{\Phi_{M}^{PMNmed-IE}}{\Phi_{M}^{alivePre-IE}}=0.71$). The combined units that were directly influenced by these rates are *P*_*N*_ and *P*_*M*_, respectively, representing pathogens in neutrophils and in monocytes. Despite these differences in the phagocytosis rates between the base models, the dynamics of *P*_*N*_ and *P*_*M*_ were similar during the first 60 minutes post infection and differed only slightly at four hours post infection (see Fig 5B and 5C). However, the phagocytosis rates were larger in the alivePre-IE model, because at *t* = 0 *min* the fraction of 12.7% ± 0.4% pathogens were alive evasive pathogens and 87.3% ± 0.4% of non-evasive pathogens in extracellular space were accessible for phagocytosis, which is a value that is smaller in comparison to the two other base models. In the spon-IE and the PMNmed-IE model, no evasive cells were present before infection. Thus, 100% of the infected pathogens were in extracellular space and could be phagocytosed. In comparison to the spon-IE model and PMNmed-IE model, the alivePre-IE model predicted a smaller fraction of extracellular pathogens (*P*_*E*_) at *t* = 240 *min* and nearly all of them were alive and evasive (see S3C Fig of S1 File for spon-IE model, S4C Fig of S1 File for PMNmed-IE model and S5C Fig of S1 File for alivePre-IE model). Furthermore, we observed differences between models in rates of killing. Compared to the spon-IE model and the PMNmed-IE model, the alivePre-IE model predicted a larger value for the rate of intracellular killing by neutrophils (*κ*_*N*_) (see Fig 4B), whereas the rate for extracellular killing by antimicrobial peptides (*κ*_*EK*_(*t*)) was smaller (see Fig 4C). This inverted ratio of intracellular and extracellular killing rates between the alivePre-IE model and the two other base models seems to compensate the effect on the dynamics of the combined unit for killed pathogens (*P*_*K*_). Consequently, the course of *P*_*K*_ during 60 minutes post infection was similar for each of these three base models (see Fig 5A). At four hours post infection, the alivePre-IE model predicted a smaller fraction of killed pathogens (*P*_*K*_ = 87.3 ± 0.4%) and a larger fraction of alive pathogens (*P*_*A*_ = 12.7 ± 0.4%) in comparison to the spon-IE model with *P*_*K*_ = 88.6 ± 0.6% and *P*_*A*_ = 11.4 ± 0.6%, and the PMNmed-IE model with *P*_*K*_ = 88.9 ± 0.9% and *P*_*A*_ = 11.1 ± 0.9%.

**Fig 6:**
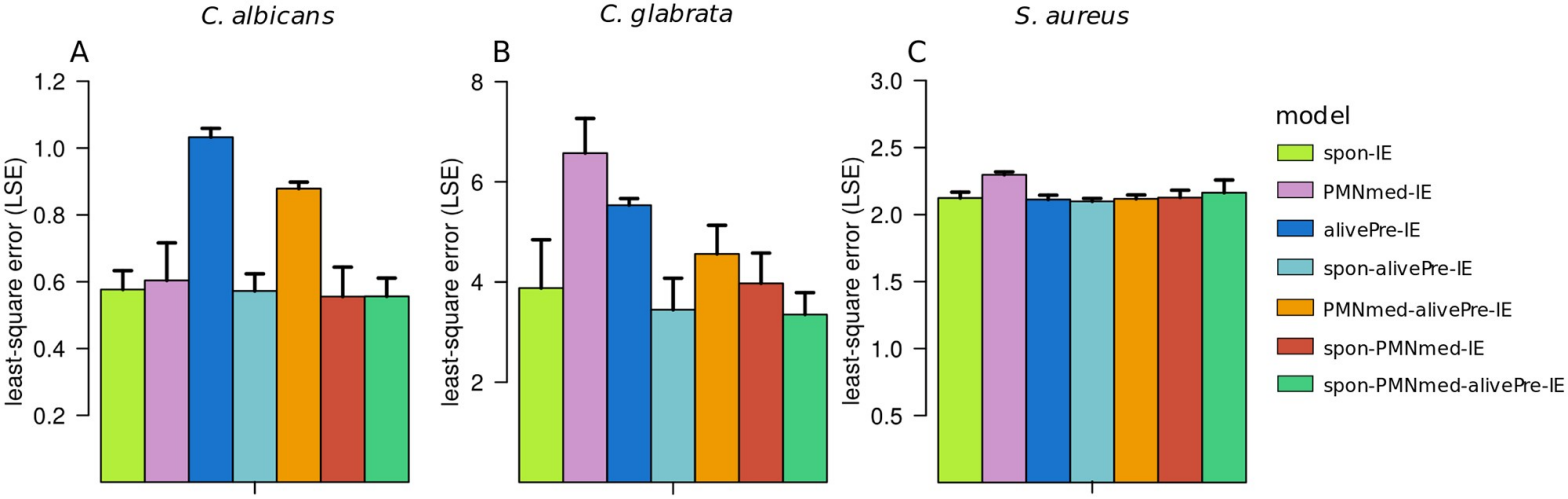
LSE values of immune evasion models resulting from calibration to infection scenarios with the different pathogens. Each bar refers to the mean LSE value ± SD of 50 simulations with transition rate values that were randomly sampled within their respective SD by the spon-IE model (light green line), PMNmed-IE model (pink line), alivePre-IE model (dark blue line), spon-alivePre-IE model (light blue line), PMNmed-alivePre-IE model (orange line), spon-PMNmed-IE model (red line) and spon-PMNmed-alivePre-IE model (dark green line). The LSE is compared for a simulated infection scenario with *C*. *albicans* (A), *C*. *glabrata* (B) and *S*. *aureus* (C).

**Fig 7:**
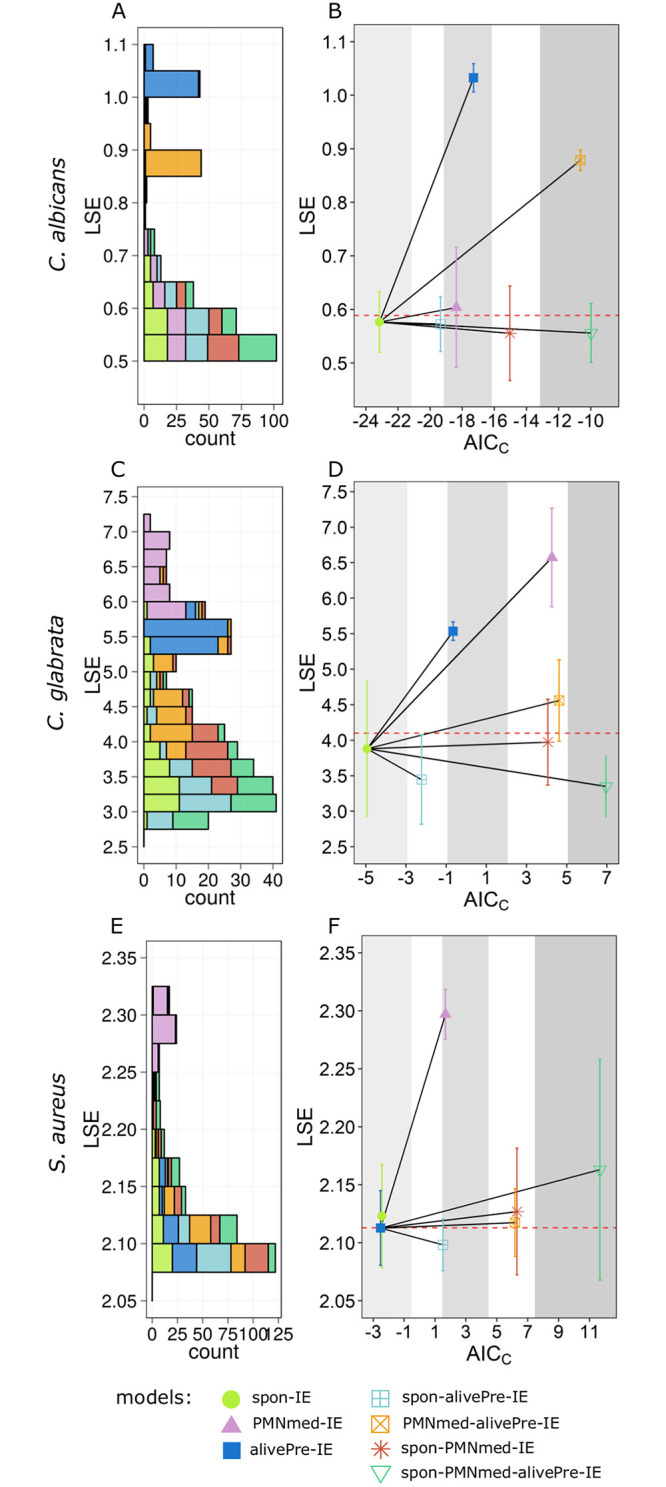
Distribution of LSE values and the *AIC*_*C*_ values for each immune evasion model. The LSE distribution of 50 simulations for each immune evasion model with transition rate values that were randomly sampled within their respective standard deviation are depicted in the left column. The colors refer to the spon-IE model (light green), PMNmed-IE model (pink bars), alivePre-IE model (dark blue), spon-alivePre-IE model (light blue), PMNmed-alivePre-IE model (orange), spon-PMNmed-IE model (red) and spon-PMNmed-alivePre-IE model (dark green). The mean of the LSE was used to calculate the corresponding *AIC*_*C*_ value for each model (same color as in the left column). The most supported model achieves the lowest *AIC*_*C*_ value and for every other model the Δ*AIC*_*C*_ value is calculated and depicted. Areas colored by different shades of grey represent the different categories of Δ*AIC*_*C*_ that are defined in the Methods section. The white regions indicate the areas between the Δ*AIC*_*C*_ categories. The horizontal red line depicts the median LSE of all LSE values. The subfigures show the LSE distribution and the *AIC*_*C*_ values for each model, respectively, for *C*. *albicans* infection (A, B), *C*. *glabrata* infection (C, D) and *S*. *aureus* infection (E, F).

Taken together, the alivePre-IE model predicted parameter values, especially for Φ_*N*_, Φ_*M*_, *κ*_*N*_ and *κ*_*EK*_(*t*), that differed in comparison to the spon-IE and the PMNmed model. Furthermore, we observed differences between these models in the simulation of the combined units *P*_*E*_, *P*_*K*_ and *P*_*A*_ (see Fig 5A–5C and S3-S5 Figs of S1 File). As described above, these differences in the parameter values and the combined units were a direct consequence of the initial presence of alive evasive pathogens at time *t* = 0 *min*. Furthermore, by comparing the degree of agreement of model simulations with the experimental data using the LSE (see Methods), we found that the alivePre-IE model simulations had the largest LSE (see Fig 6A) and, moreover, this was mainly caused by deviations in the combined units *P*_*E*_ and *P*_*A*_, which were shown to be mainly affected by the presence of evasive pathogens that are alive. In other words, the model could not fill in the gap between extracellular pathogens (15.0±5.8%) and alive pathogens (6.5±4.2%) at t = 240 min post infection. In addition to the LSE, we compared the goodness of the models using the *AIC*_*C*_ (corrected Akaike information criterion, see Methods), which involves not only the deviation to the experimental data but also the number of model parameters (see Methods). We observed that the spon-IE model not only had the smallest LSE, among the three base models (see Figs 6 and 7A), but also showed the smallest *AIC*_*C*_ (see Fig 7A). The goodness of the two other base models was evaluated by their Δ*AIC*_*C*_, which is defined as the absolute difference to the model with the smallest *AIC*_*C*_, *i*.*e*. the spon-IE model. The differences Δ*AIC*_*C*_ = 4.78 for the PMNmed-IE model and Δ*AIC*_*C*_ = 5.86 for the alivePre-IE model show that these models are in the category of models with *considerably less support* in comparison to the spon-IE model (see Methods).

By involving the results of the four combined immune-evasion models into the comparative analysis, we observed that the PMNmed-alivePre-IE model predicted killing rates that have also an inverse relationship to the other combined models (see Fig 4B and 4D), but the resulting dynamics of *P*_*K*_ was not affected (see Fig 5A).

However, similar to the alivePre-IE model, the PMNmed-alivePre-IE model predicted a smaller fraction of immune-evasive cells (*P*_*IE*_) in comparison to the other models (see Fig 5D). This resulted in much larger LSEs of these two models (see Fig 6A). For example LSEs of the alivePre-IE model (*P*′ < 0.001) and PMNmed-alivePre-IE model (*P*′ < 0.001) are significantly different in comparison to the LSE of the PMNmed-IE model and this is also the case when comparing to the other models. Since the complexity of the PMNmed-alivePre-IE is also large (nine parameters), the difference Δ*AIC*_*C*_ to the model with the smallest *AIC*_*C*_, which is the spon-IE model, indicates that this model is in the category of models that have *essentially no support* (see Methods for the respective categorization). The spon-alivePre-IE model simulations caused a similar LSE to the spon-IE model simulations and the corresponding Δ*AIC*_*C*_ is the smallest among the compared models. The other combined models have an LSE similar to the spon-IE model, but are either close to or within the category of models that have *essentially no support* according to their Δ*AIC*_*C*_ values.

Taken together, we observed that the immune-evasion models can be calibrated to the experimental data of whole-blood infection with *C*. *albicans*. However, differences in the parameter values caused differences in the goodness of the fit with respect to the LSE and the *AIC*_*C*_. We found that the spon-IE model simulations caused the smallest LSE and the best *AIC*_*C*_. Since no killed evasive cells are allowed in the alivePre-IE model, the simulated kinetics causes the highest LSE. The PMNmed-alivePre-IE model and the spon-PMNmed-alivePre-IE model are the evasion models with the largest complexity, *i*.*e*. with the largest number of model parameters, causing the highest *AIC*_*C*_ values. The respective difference to the best *AIC*_*C*_ value, the Δ*AIC*_*C*_, indicates that these models belong to the category of models with *essentially no support*.

### Whole-blood infection with *C*. *glabrata*

**Fig 8:**
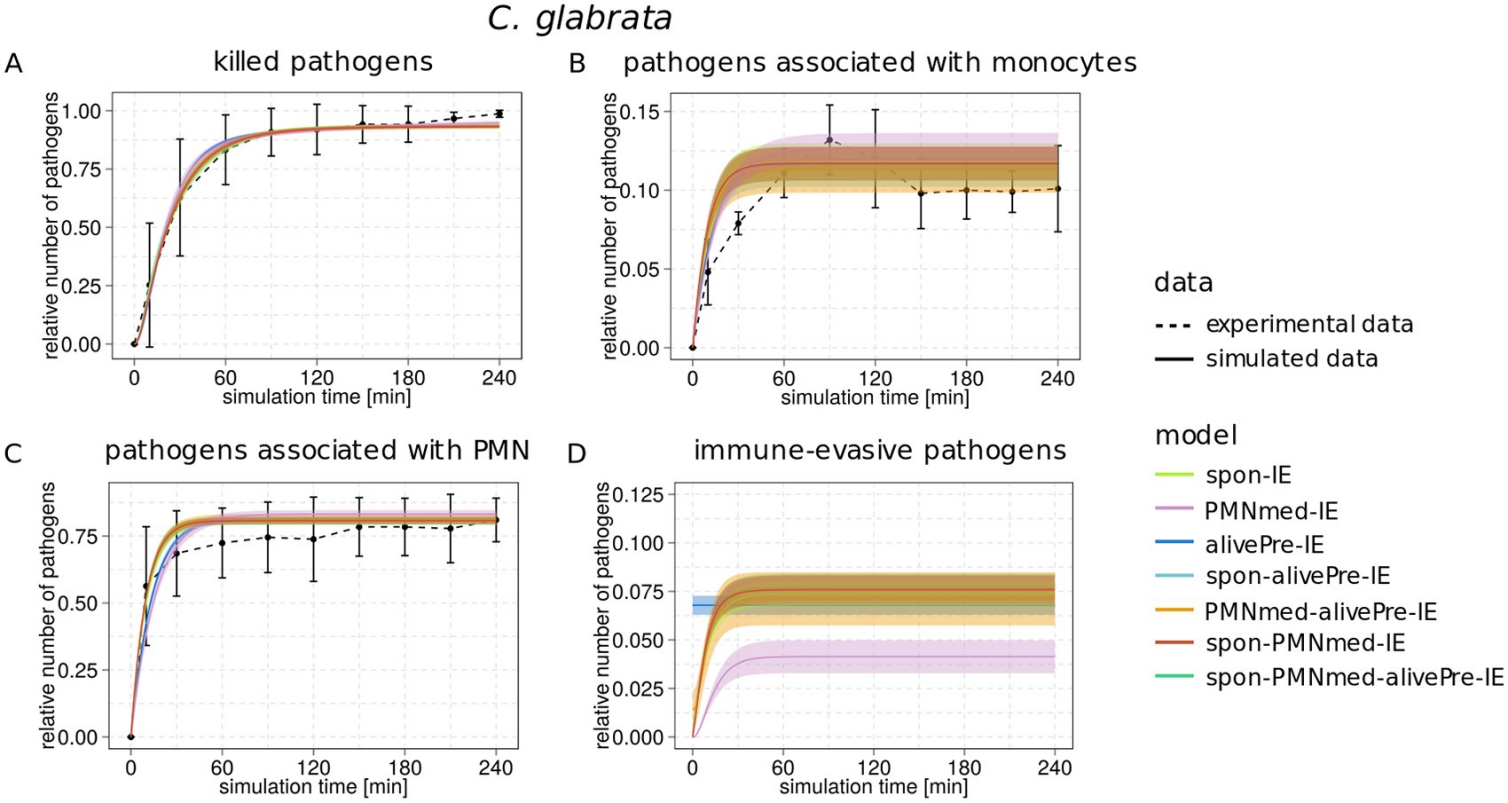
Kinetics of combined units of each immune-evasion model for infection scenario with *C*. *glabrata*. Experimental data from whole-blood infection assays (dashed lines) with corresponding standard deviations (SDs) are compared to the simulated data (solid lines) for *C*. *glabrata* infection scenario. The simulated kinetics by the spon-IE model (light green line), PMNmed-IE model (pink line), alivePre-IE model (dark blue line), spon-alivePre-IE model (light blue line), PMNmed-alivePre-IE model (orange line), spon-PMNmed-IE model (red line) and spon-PMNmed-alivePre-IE model (dark green line) are shown in their corresponding color. The thickness of solid lines indicates the mean ± SD of 50 simulations with transition rate values that were randomly sampled within their respective SD. Simulated kinetics of killed cells (A), pathogens associated with monocytes (B), pathogens associated with PMN (C) and immune-evasive cells (D) for a *C*. *glabrata* infection.

In our previous study, we found remarkable differences in the model predictions for *C*. *glabrata* infection regarding the transition rate values–especially for the rate of extracellular killing–from the spon-IE model and the PMNmed-IE model. In this study, we additionally compared the predictions from the alivePre-IE model and found its transition rate values to be either similar to or between the respective values quantified by the two other base models (see Fig 4F–4J). For example, the phagocytosis rate of neutrophils (Φ_*0*_) was predicted to be highest in the spon-IE model, followed by the alivePre-IE model and lowest in the PMNmed-IE model ($\Phi_{N}^{spon}>\Phi_{N}^{alivePre}>\Phi_{N}^{PMNmed}$). Consequently, this led to a quicker increase in the dynamics of *P*_*N*_ for the spon-IE model in comparison to the alivePre-IE model and the PMNmed-IE model, which is also visible by comparing the fraction of *P*_*N*_ at ten minutes post infection ($P_{N}^{spon}=50.4\pm 2.0\%$, $P_{N}^{alivePre}=42.5\pm 1.4\%$, $P_{N}^{PMNmed}=38.6\pm 2.2\%$) as shown in Fig 8C. However, four hours post infection with *C*. *glabrata*, the fraction of *P*_*N*_ was largest in the model with the smallest phagocytosis rate, *i*.*e*. the PMNmed-IE model. This was caused by additionally predicting a lower immune evasion rate in comparison to the spon-IE and the alivePre-IE model (see Fig 4H), which consequently gave the neutrophils and monocytes more time to phagocytose *C*. *glabrata* cells.

Despite the fact that the model dynamics of the combined units appear similar for the base models, the quantitative differences in the transition rate values are associated with somewhat different LSEs (see Fig 6B). The LSE of the alivePre-IE model is smaller than the LSE of the PMNmed-IE model and larger than the LSE of the spon-IE model. By additionally involving the model complexity into the model assessment using the *AIC*_*C*_ (see Methods), we found that the spon-IE model had the lowest *AIC*_*C*_, which indicates that the corresponding LSE is small enough to compensate for the higher number of parameters in the spon-IE model (see Table 1) in comparison to the alivePre-IE model. Moreover, the absolute difference between *AIC*_*C*_ values of the alivePre-IE model and the spon-IE model (${\Delta AIC}_{C}^{alivePre}=3.4$) indicates that the alivePre-IE model has less support than the spon-IE model (see Fig 7D). The PMNmed-IE model not only showed a larger LSE value than the spon-IE model, but also a higher number of parameters. Thereby, the *AIC*_*C*_ is larger and, moreover, the corresponding ${\Delta AIC}_{C}^{PMNmed}$ lies between the two categories referred to as *considerably less support* and *essentially no support*.

Interestingly, the four combined models of immune evasion predicted values for Φ_*N*_, Φ_*M*_ and *κ*_*N*_ that were markedly different from the values predicted by the two base models, alivePre-IE and PMNmed-IE model (see Fig 4E–4G). By comparing the LSE, we found that simulations of all four combined models have LSEs that differ from the spon-IE model, which is the base model with the lowest LSE. The statistical analysis revealed that this difference is significant with *P*′ < 0.01 for the spon-alivePre-IE model and with *P*′ < 0.001 for the PMNmed-alivePre-IE model, the spon-PMNmed-IE model and the spon-PMNmed-alivePre-IE model. However, the combined models contain more model parameters and, therefore, their *AIC*_*C*_ values are larger in comparison to that of the spon-IE model. The spon-alivePre-IE model has a lower LSE and thus the larger model complexity was compensated in comparison to the spon-IE model. The corresponding Δ*AIC*_*C*_ = 2.7 clearly shows the proximity to the category of models with *substantial support*. All other combined models failed to compensate for their model complexity with a better fit and are either close to or within the category of models with *essentially no support* (see Fig 7D).

Taken together, the alivePre-IE model made predictions that are quantitatively between those of the two other base models regarding transition rate values, LSE and *AIC*_*C*_. The largest differences were found for the rates of intracellular killing, extracellular killing and immune evasion. The spon-IE model was the best according to the *AIC*_*C*_ and the spon-alivePre-IE model was comparable with this model according to the categorization of the Δ*AIC*_*C*_ (see Methods). All other combined models contain more parameters and this larger complexity could not be compensated by a better fit to experimental data, leading to a larger difference Δ*AIC*_*C*_.

### Whole-blood infection with *S*. *aureus*

**Fig 9:**
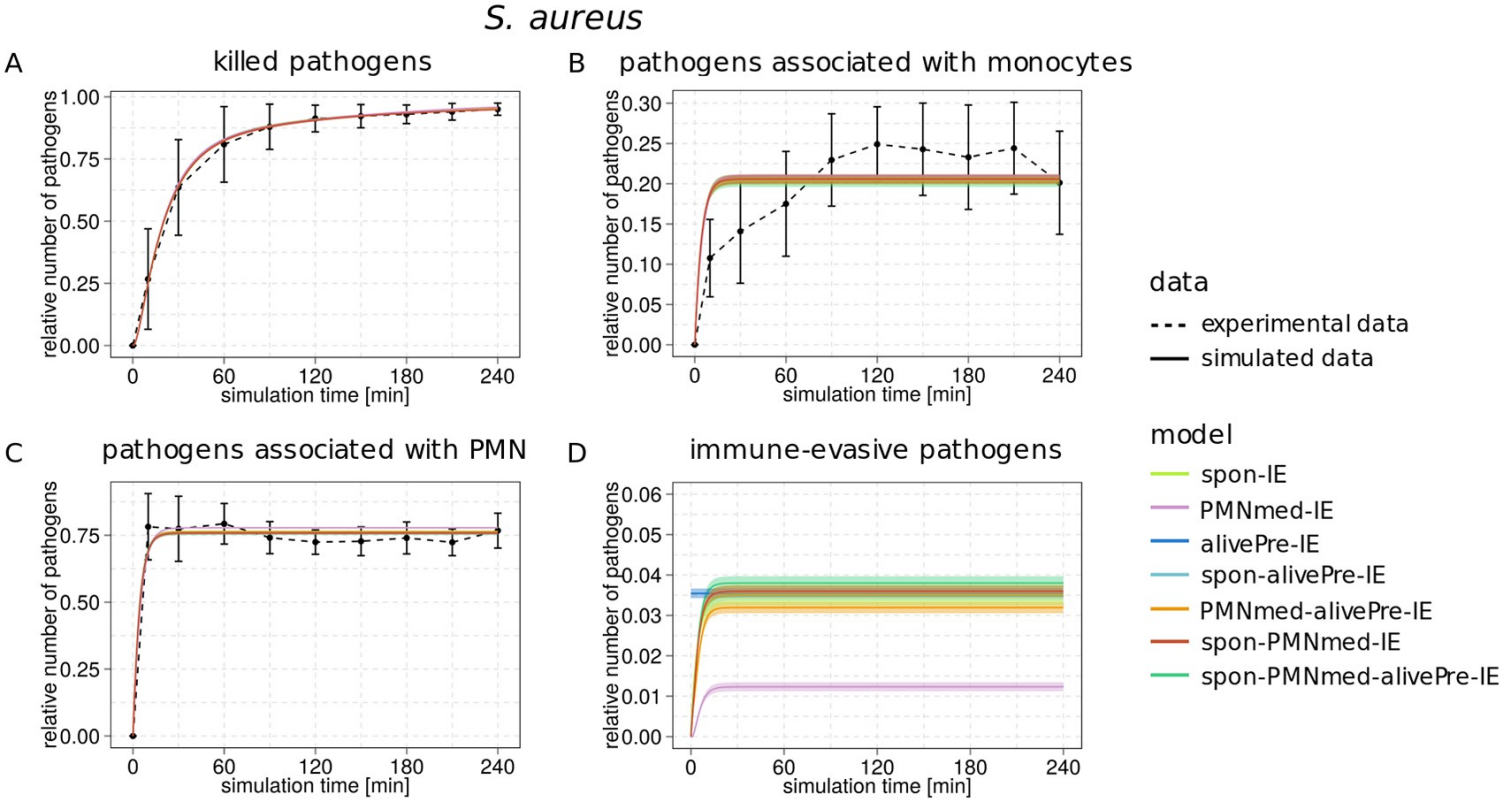
Kinetics of combined units of each immune-evasion model for infection scenario with *S*. *aureus*. Experimental data from whole-blood infection assays (dashed lines) with corresponding standard deviations (SDs) are compared to the simulated data (solid lines) for the *S*. *aureus* infection scenario. The simulated kinetics by the spon-IE model (light green line), PMNmed-IE model (pink line), alivePre-IE model (dark blue line), spon-alivePre-IE model (light blue line), PMNmed-alivePre-IE model (orange line), spon-PMNmed-IE model (red line) and spon-PMNmed-alivePre-IE model (dark green line) are shown in their corresponding color. The thickness of solid lines indicates the mean ± SD of 50 simulations with transition rate values that were randomly sampled within their respective SD. Simulated kinetics of killed cells (A), pathogens associated with monocytes (B), pathogens associated with PMN (C) and immune-evasive cells (D) for a *S*. *aureus* infection.

Extending to the infection scenario with *S*. *aureus* in this study, the immune reaction rates were quantified by calibrating the models to the observed association and killing kinetics of *S*. *aureus* cells. We found that the optimal reaction rate values of the spon-IE model and the alivePre-IE model showed a smaller deviation from each other than in comparison to the rates of the PMNmed-IE model. As can be seen in Fig 4I, the phagocytosis rates of monocytes (Φ_*M*_) and PMNs (Φ_*N*_) are smaller for the PMNmed-IE model in comparison to the other base models. Furthermore, the rate for immune evasion is smaller in the PMNmed-IE model (see Fig 4L). The evasion rate in this model is dependent on the first-phagocytosis events by PMNs, which mainly occurred during the first hour post infection, resulting in an immune-evasion rate close to zero at one hour post infection. At later times, extracellular pathogens (*P*_*AE*_ and *P*_*KE*_) were phagocytosed predominantly by immune cells, *i*.*e*. mainly by neutrophils. This led to a larger fraction of *P*_*N*_ for the PMNmed-IE model in comparison to the spon-IE model and the alivePre-IE model (see Fig 9). Additionally, the PMNmed-IE model predicted an optimal value for the intracellular killing rate for monocytes (*κ*_M_) that was the smallest compared to the other base models, whereas the rate of extracellular killing (*κ*_*EK*_(*t*)) was the largest. However, these differences do not have an effect on the resulting model dynamics with regard to the killed pathogens (*P*_*K*_), which is almost equal for all three base models (see Fig 9A). The LSE is larger for the PMNmed-IE model in comparison to the spon-IE model (*P*′ < 0.001) and the alivePre-IE model (*P*′ < 0.001). The three base models have similar LSE values that are not significantly different (*P*′ > 0.05) (see Fig 6C). However, in comparison to the other infection scenarios (see Fig 6A and 6B), the differences between the LSEs of the base models are smaller. Simulations of the alivePre-IE model led to the smallest *AIC*_*C*_ value being similar to the *AIC*_*C*_ of the spon-IE model with difference Δ*AIC*_*C*_ = 0.09, indicating that this model belongs to the category of models with *substantial support* (see Fig 7E and 7F). We observed a difference of Δ*AIC*_*C*_ = 4.19 for the PMNmed-IE model, indicating that this model belongs to the category of models with *considerably less support* than the alivePre-IE model with the smallest *AIC*_*C*_ value.

The combined models of immune evasion could be calibrated to the whole-blood infection assays with *S*. *aureus*, resulting in LSEs that are not significantly different from those of the model with the smallest LSE, *i*.*e*. the spon-alivePre-IE model. Considering the *AIC*_*C*_ score, we observed that this model is better than the other combined models, because the difference Δ*AIC*_*C*_ classifies the model in the category of *considerably less supported* models, whereas the other combined models where classified in the category of models with *essentially no support*.

Taken together, for the infection scenario with *S*. *aureus*, we observed similar dynamics for all models with predicted reaction rates that are mostly similar. We found only minor differences in the predictions from the PMNmed-IE model. Furthermore, we observed that for *S*. *aureus* whole-blood infection the alivePre-IE model simulation led to the smallest *AIC*_*C*_, being close to the *AIC*_*C*_ of the spon-IE model.

### Patterns of immune reaction rates for the three infection scenarios are highly model-specific

**Fig 10:**
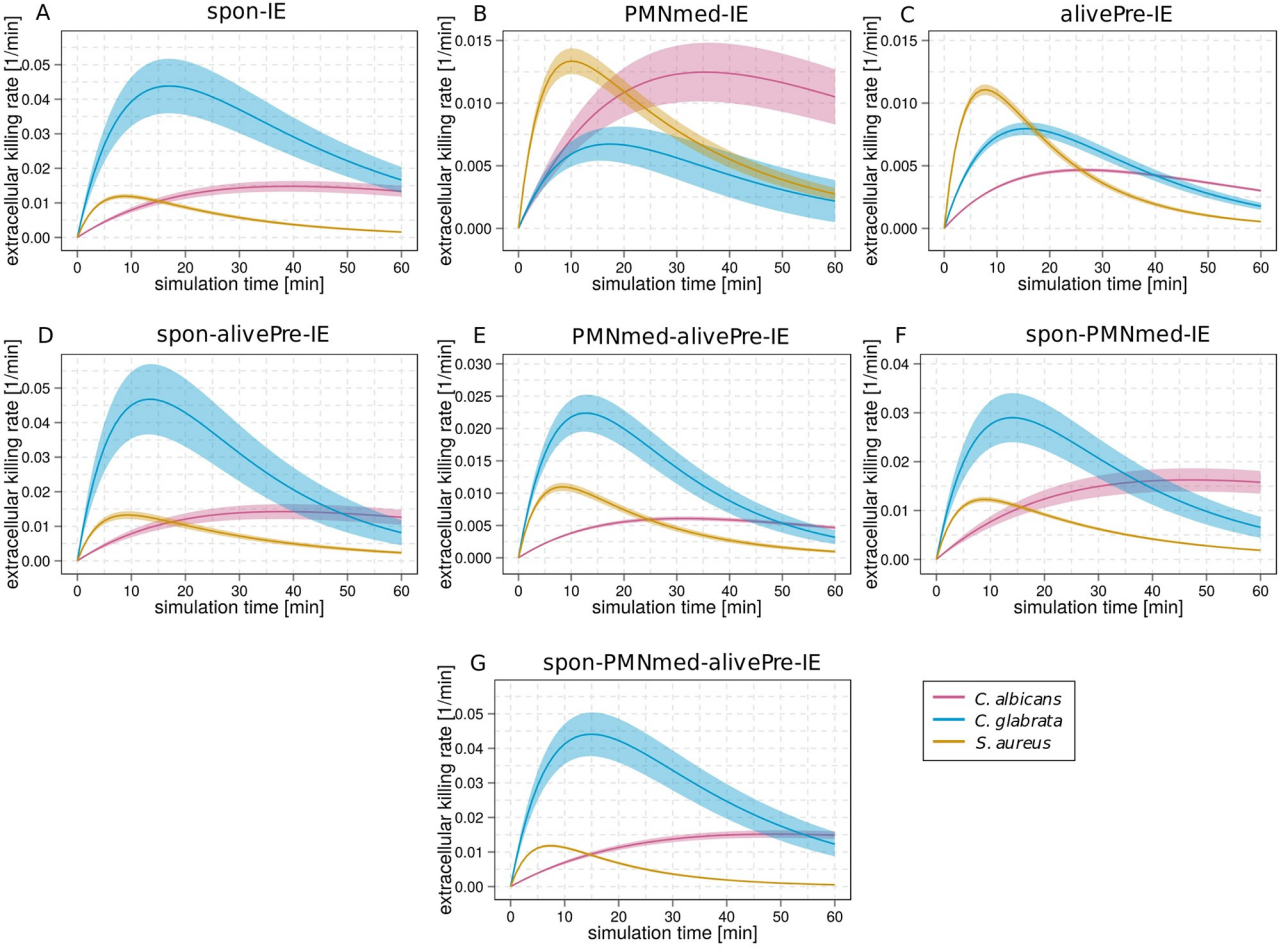
Kinetics of extracellular killing rates estimated by immune evasion models for the three infection scenarios. The predicted extracellular killing rate *κ*_*EK*_(*t*) is shown for an infection with *C*. *albicans* (pink line), *C*. *glabrata* (blue line) or *S*. *aureus* (orange line). The subfigures show the time course of extracellular killing for the spon-IE model (A), the PMNmed-IE model (B), the alivePre-IE model (C), the spon-alivePre-IE model (D), the PMNmed-alivePre-IE model (E), the spon-PMNmed-IE model (F) and the spon-PMNmed-alivePre-IE model (G). The thickness of the lines indicates the mean ± standard deviations obtained by 50 simulations with normally distributed transition rates.

After comparing the predictions by the various models for each infection scenario, we compared the predictions for all infection scenarios for each model separately. This is helpful in order to identify pathogen-specific patterns that differ between the immune evasion models. First, we compared the values of the rate of extracellular killing by antimicrobial peptides for the infection with *C*. *albicans*, *C*. *glabrata* and *S*. *aureus* at specific time points post infection. As shown in Fig 10A, the spon-IE model predicted the largest value of the extracellular killing rate for *C*. *glabrata* infection and the smallest value for *C*. *albicans* infection ($\kappa_{EK}^{C.g.}(t)>\kappa_{EK}^{S.a.}(t)>\kappa_{EK}^{C.a.}(t)$ at *t* = 10 *min*) ten minutes post infection. In contrast, the PMNmed-IE model predicted a larger value of the extracellular killing rate for infection with *S*. *aureus* in comparison to *C*. *albicans* and *C*. *glabrata* infection, while the latter two rates are not significantly different from each other ($\kappa_{EK}^{S.a.}(t)>\kappa_{EK}^{C.g.}(t)=\kappa_{EK}^{C.a.}(t)$ at *t* = 10 *min0*) (see Fig 10B). The third base model, the alivePre-IE model, also predicted that the infection by *S*. *aureus* cells leads to the largest extracellular killing rate, but the values for infection by *C*. *glabrata* and *C*. *albicans* are significantly different ($\kappa_{EK}^{S.a.}(t)>\kappa_{EK}^{C.g.}(t)>\kappa_{EK}^{C.a.}(t)$ at *t* = 10 *min*) (see Fig 10C).

As shown in Fig 10D–10G, the various combined immune evasion models predicted strikingly similar relations of the extracellular killing rates between the infection scenarios at ten minutes post infection. Similar to the pattern predicted by the spon-IE model, infection with *C*. *glabrata* induced a larger extracellular killing rate than infection with *S*. *aureus*, which in turn is larger than the value for *C*. *albicans* infection, *i.e. $\kappa_{EK}^{C.g.}(t)>\kappa_{EK}^{S.a.}(t)>\kappa_{EK}^{C.a.}(t)$* at *t* = 10 *min*. However, additional evaluation of the relation of extracellular killing rates between the pathogens at one hour post infection revealed that the spon-PMNmed-IE model predicted another pattern than the other four models. The extracellular killing rate is larger for infection with *C*. *albicans* in comparison to infection with *C*. *glabrata* (with *P*′ < 0.001) (Fig 10F). The other three combined immune evasion models (Fig 10D, 10E and 10G) and the spon-IE model (Fig 10A) predicted similar values for *C*. *albicans* and *C*. *glabrata* at one hour post infection. Therefore, we conclude that the rate for extracellular killing provides a marker that enables to differentiate between three immune evasion models, *i*.*e*. the PMNmed-IE model, the alivePre-IE model and the spon-PMNmed-IE model. Moreover, the other four models predicted a unique pattern, which in turn is distinct from the previous three models.

**Fig 11:**
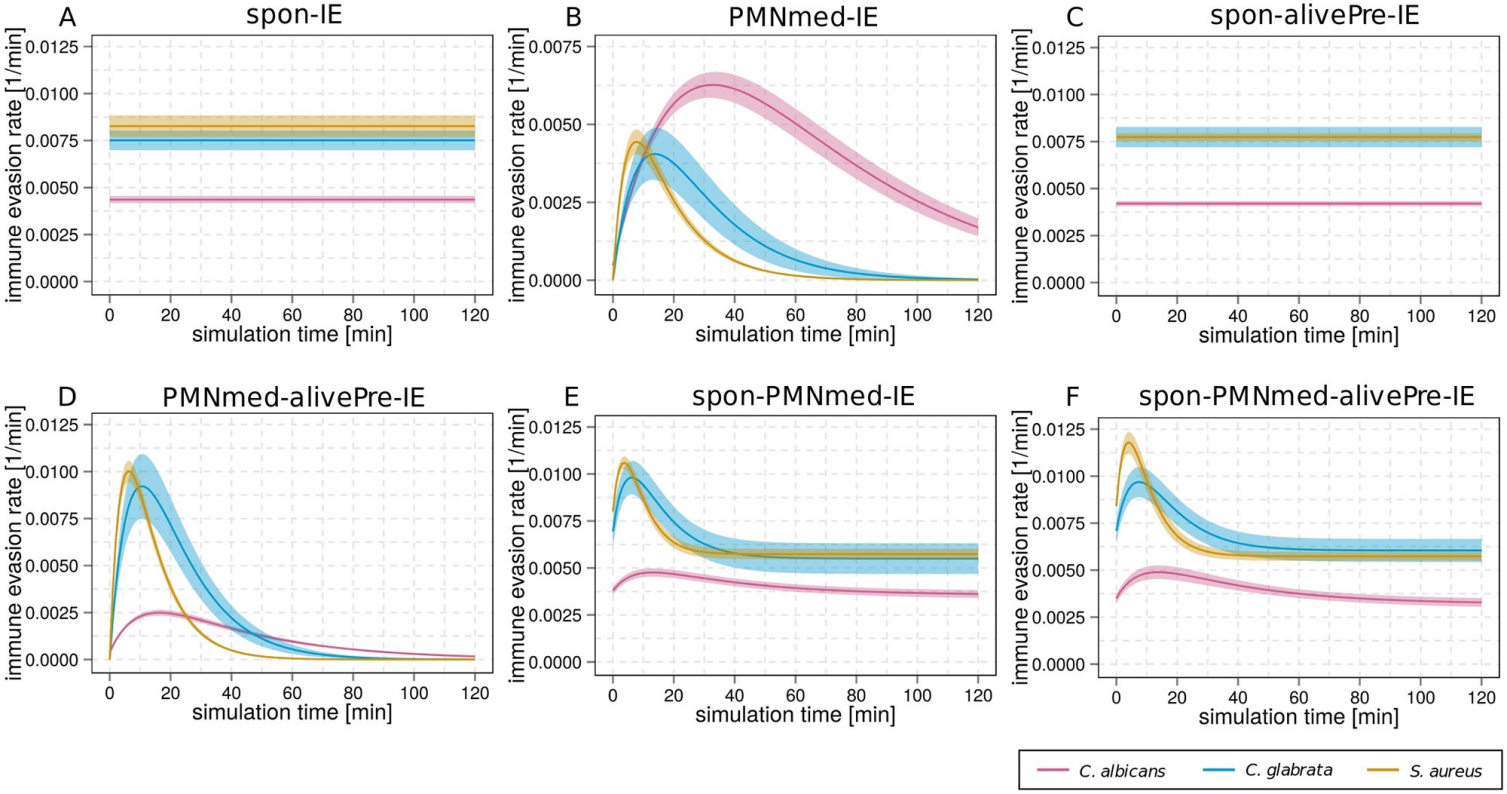
Kinetics of the immune-evasion rate estimated immune evasion models for the three infections scenarios. The predicted immune-evasion rate is shown for an infection with *C*. *albicans* (pink line), *C*. *glabrata* (blue line) or *S*. *aureus* (orange line). The subfigures show the results for the spon-IE model (A), the PMNmed-IE model (B), the alivePre-spon-IE model (C), the PMNmed-alivePre-IE model (D), the spon-PMNmed-IE model (E) and the spon-PMNmed-alivePre-IE model (F). Note that the alivePre-IE model does not contain a rate for the acquisition of immune evasion and therefore it is not included in this figure.

By comparing the relation of immune evasion rates between the models, we identified more model-specific patterns. Since the PMNmed-IE model, the alivePre-IE model and the spon-alive-IE model can be clearly differentiated based on the rate for extracellular killing, we here focus on the four models that have a unique pattern for extracellular killing, *i*.*e*. the spon-IE model (Fig 11A), the spon-alivePre-IE model (Fig 11C), the PMNmed-alivePre-IE model (Fig 11D) and the spon-PMNmed-alivePre-IE model (Fig 11F). The spon-IE model and the spon-alivePre-IE model have immune evasion rates that are constant during infection and the predicted patterns for the relation between the infection scenarios are similar. However, because of the constant nature of the immune evasion rate, these models can be differentiated from the PMNmed-alivePre-IE model and the spon-PMNmed-alivePre-IE model with their time-dependent immune evasion rates (see Fig 11D and 11F). Additionally, we observed for the spon-PMNmed-alivePre-IE model that the order of the immune evasion rates for the different infection scenarios was constant during infection, while the PMNmed-alivePre-IE model predicted that this order changed during infection. As can be seen in Fig 11D, ten minutes post infection the rate for *C*. *albicans* infection is the smallest in comparison to *C*. *glabrata* and *S*. *aureus* infection, while from 70 minutes post infection, the rate is the largest for *C*. *albicans*.

Additionally, we compared the relation of the other transition rates between the infection scenarios and we found that either all models predicted a similar pattern, such as for Φ_*M*_ and Φ_*N*_ with Φ^S.a.^ > Φ^*c*.*g*.^ > Φ^*c*.*a*.^, or the differences are not sufficient to differentiate between the spon-IE model and the spon-alivePre-IE model.

Taken together, the analysis of the relation of immune reaction rates between the three infection scenarios separately for each immune evasion model enabled us to identify distinct patterns for most immune evasion models. We found unique patterns for the PMNmed-IE model, the alivePre-IE model and the spon-PMNmed-IE model by analyzing the model predictions for the rate of extracellular killing. While the spon-IE model and the spon-alivePre-IE model predict similar patterns for all immune reaction rates, evaluating the rate for immune evasion revealed unique patterns for the PMNmed-alivePre-IE model and the spon-PMNmed-alivePre-IE model. This means that five out of seven immune-evasion models showed unique patterns for the rates of extracellular killing and immune evasion.

## Discussion

We applied a systems biology approach in order to assess hypotheses on the mechanism of immune evasion of pathogens in human whole-blood infection assays. To this end, we implemented different hypotheses into virtual infection models and comparatively assessed the quantitative model predictions for three different infection scenarios. In addition to infection scenarios with the fungal pathogens *C*. *albicans* and *C*. *glabrata*, which were already under investigation in previous studies [1, 2, 4, 5, 12], we infected human whole-blood samples with the bacterial pathogen *S*. *aureus*. By applying the same experimental setup and measurement procedures as for the fungal pathogens, we were able to directly compare the measured kinetics for pathogen survival and immune cell association. We found bacteria-specific characteristics, such as a quicker and even higher association with immune cells, in particular, with monocytes. Consequently, the fraction of *S*. *aureus* that was not associated with immune cells but remained extracellularly was relatively small. Since only these extracellular pathogens can be immune-evasive, we conclude that the development of an immune-evasive phenotype plays a minor role in the response to *S*. *aureus* infection in human whole blood.

In addition to our previous models of the immune evasion of pathogens in whole blood, *i*.*e*. the spon-IE model with spontaneous and the PMNmed-IE model with PMN-mediated immune evasion, we here implemented the pre-IE model. This model represents the hypothesis that evasive pathogens do not evolve during immune response, but rather are present as a subpopulation in the whole pathogen population even before infection. It should be noted that this hypothesis has been frequently put forward to explain the existence of immune-evasive pathogens against the possibility that immune evasion is the result of some process going on during the time course of the human whole-blood assay. By calibrating the pre-IE model to the experimental measurements of the three whole-blood infection scenarios, we could predict not only the fraction of pathogens that were immune-evasive, but also the fractions of these evasive cells that need to be alive and killed before infection. In the spirit of an image-based systems biology approach [15, 16], we made use of microscopy imaging and automated image analysis to test the model predictions. In this way we could falsify our initial hypothesis by measuring the true fraction of killed pathogens before infection. Since it turned out that there were almost no killed pathogens measured before infection–rather that this fraction is smaller as the fractions of extracellular pathogens and killed immune-evasive pathogens–the pre-IE model with alive and killed evasive pathogens needed to be rejected. Based on these results, we were able to start a new lap within the cycle of systems biology and tested the hypothesis of having a subpopulation of exclusively alive evasive cells within the so-called alivePre-IE model.

Next, we compared the model predictions for the three immune evasion models, each containing only one evasion mechanism and referred to as the three base models. However, since it cannot be excluded that more than one mechanism causes immune evasion of pathogens, we also generated models for each possible combination of the three evasion mechanisms, which we refer to as combined models. These contain at least two of the evasion mechanisms of the base models, leading to an increased model complexity that is reflected by a larger number of model parameters.

The models were again calibrated to experimental data of the three infection scenarios and in order to comparatively assess the quality of each model, we used the agreement with the experimental data in terms of the least-square error (LSE). However, it is evident that this measure alone can overvalue models with larger numbers of parameters [17–19], because these models have a larger degree of freedom and thereby may be calibrated to the experimental data with higher accuracy. We cope with the differing number of parameters of our models including the corrected Akaike Information criterion (*AIC*_*C*_) as a second measure. This measure involves a penalty for the number of model parameters, which is even more pronounced as for the Bayesian information criterion [20]. Although such information criteria are powerful tools for model selection [21], it should be noted that, in contrast to other studies [22–25], we do not consider an information criterion that penalizes the number of model parameters as a sufficient argument against a particular model. Information criteria measure the quality of a model from a mathematical point of view, without considering that particular biological processes may inevitably be associated with a certain number of required parameters. Furthermore, there are specific sources of uncertainty that must be considered in the course of finding the best model for a biological system by model calibration to experimental data. Warne *et al*. [19], described four key sources of uncertainty, which are 1) unknown model parameter values, 2) uncertainty in the model choice, 3) uncertainty that arise by the stochastic nature of the model and 4) uncertainties that are caused by systemic or measurement errors during the biological experiments [19]. We accounted for all these four sources of uncertainty by 1) applying a global estimation algorithm in order to provide optimal prerequisites for the search of unknown model parameters, 2) implementing various reasonable models in order to provide a set of potential models, 3) simulating the model with a large number of individuals to reduce the impact of stochasticity on the average model output and 4) repeating the experiments to account for biological uncertainties. Furthermore, the epistemic uncertainty of the model kinetics, which is caused by variation of the transition rate values [26, 27], was considered by performing 50 simulations with transition rate values that were randomly sampled within their respective standard deviation. In order to investigate how these uncertainties were caused by the different transition rates, a sensitivity analysis could be performed [26, 28, 29]. Since this analysis step does assess the transition rates but not the immune-evasion models in a comparative way, it would not help to reject or select immune-evasion mechanisms. Once the immune-evasion mechanism is experimentally validated by future studies, a sensitivity analysis should be performed. Furthermore, after reducing the number of immune evasion models, a long-term analysis of the model dynamics should be the focus of future studies. However, it is commonly known that this analysis requires a huge computational effort. Cornell *et*. *al*. [30] describe a method to analyse the dynamics of such individual-based models in a mathematical way. This more efficient method could be applied in future studies.

The calibration of the eight immune-evasion models to the three infection scenarios revealed model-specific predictions and differences in the quality of the fit to experimental data for each infection scenario. For infection with *C*. *albicans*, the spon-IE model showed the smallest LSE and *AIC*_*C*_ and may therefore be prioritized over the other models, whereas the alivePre-IE model is by far the model with the largest LSE and *AIC*_*C*_. The spon-IE model and the spon-alivePre-IE model are the models with the smallest LSE and the best *AIC*_*C*_ for the infection scenario with *C*. *glabrata*. Moreover, for infection with either of the two fungal pathogens, we observed that the more complex models could not compensate for their penalized complexity by a better agreement with the experimental data in comparison to the simpler immune evasion models. Similarly, for the infection scenario with *S*. *aureus*, we observed that the spon-IE model and the alivePre-IE model had the smallest LSE and *AIC*_*C*_, while in general all model predictions are more similar to each other in comparison to the other two infection scenarios.

In summary, even though we found differences in the model predictions for each infection scenario, it is not possible to either reject a model or to unambiguously identify one true model for all infection scenarios, because the majority of the models yielded simulation results within the experimentally measured standard deviations. However, we found that the differences between the models were larger for the infection scenarios with larger fractions of extracellular pathogens at four hours post infection, because the fraction of pathogens that can become evasive is larger in these scenarios. Infection scenarios with fungal pathogens, especially *C*. *albicans*, showed a larger fraction of extracellular cells and the differences between the model predictions and the corresponding LSEs and *AIC*_*C*_ are much larger in comparison to the infection scenario with *S*. *aureus*. This implies that the fungal pathogens seem much more suitable for further investigations of immune evasion mechanisms in human whole-blood assays. Nevertheless, by performing a whole-blood *S*. *aureus* infection in addition to the fungal infection scenarios, we found that these could still provide decisive information for the identification of the true evasion model or the rejection of unsuitable models. By analyzing specific model predictions for each of the three infection scenarios in a comparative fashion, we identified model-specific patterns. The relation between the infection scenarios was model-specific for the kinetics of the extracellular killing rate at ten and 60 minutes post infection and for the immune evasion rate. Thus, we identified unique patterns for five out of seven immune-evasion models, *i*.*e*. for the three base models, the spon-IE model, the PMNmed-IE model and the alivePre-IE model, as well as for two combined models, the spon-PMNmed-IE model and the spon-alivePre-IE model. For future studies, we suggest to exploit the pathogen-specific immune response in human whole-blood infection assays by extending the types of pathogens. For example, experimental investigations could also be done for Gram-negative bacteria, such as *Escherichia coli*, which was recently the focus of a study on infection assays with avian whole blood [31]. It will be interesting to see whether including more types of pathogens will extend the pathogen-specific parameter space, allowing to differentiate between mathematical models whose distinct immune-evasion mechanisms are currently not experimentally distinguishable.

Based on these predictions, we can now guide validation experiments which could reject or accept any of the models or even give rise for suggesting other immune-evasion models. For example, one can comparatively analyze the growth of pathogens within activated serum, which was previously enriched with antimicrobial peptides in response to an infection. By using serum without any immune cells, a decrease in the number of pathogens could only be caused by extracellular antimicrobial activity. Note that we can compare these measurements with our predictions in qualitative way. In contrast, determining the concentration of known antimicrobial peptides that were released upon first phagocytosis by neutrophils–like lactoferrin, elastase 2 and myeloperoxidase–would not provide direct information about their activity in pathogen killing. A comparative analysis of the time-dependent killing effect of antimicrobial peptides would still help rejecting or accepting three of the immune evasion models, *i*.*e*. the PMNmed-IE model, the alivePre-IE model and the spon-PMNmed-IE model. Two further models, the spon-IE models and the spon-alivePre-IE model, can be tested by additionally analyzing the immune evasion rate. Unfortunately, the experimental measurement of this rate is currently challenging, because the characteristics of immune-evasive pathogens are largely unknown. Furthermore, if the concentration of evasive pathogens could be measured, it could be possible to investigate whether pathogens become immune-evasive in activated or non-activated serum. Subsequently, one could compare the relative development of evasive pathogens in each of these media and compare qualitatively with the predictions of the various models. Finally, we have shown that the model-specific patterns of the relation of reaction rates between the three infection scenarios can be used as key data to navigate future experimental measurements, which in turn could validate the immune evasion models and ultimately identify the mechanism of immune evasion.

## Conclusion

Systems biology integrates computer-aided and experimental investigations to decipher mechanistic relationships in complex biological systems. With a focus on the network of immune reactions in terms of a state-based model, we investigated the undesired occurrence of immune evasion by pathogens in human bloodstream infections. We have already identified this phenomenon in previous studies, but one of the unanswered questions was whether these observations may be an artifact of the experimental procedure. In this study, we designed models and performed computer simulations to predict the consequences of assumptions, such as the initial existence of a fraction of immune-evasive pathogens. Subsequent imaging experiments revealed that this assumption cannot fully explain our observations in human whole-blood assays. The possibility to easily modify the structure of a mathematical model enabled making concrete predictions for additionally included mechanisms of immune evasion. Based on these predictions obtained from quantitatively estimating model parameters that best fit the experimental data, we derived model-specific patterns and propose new experiments that will contribute to the identification of immune-evasion mechanism in the future. From a general perspective, our present study is a prime example of applying the systems biology approach with the aim of directing research towards the identification of mechanisms in complex biological systems.

## Materials and methods

### Fungal strains and culture

*C*. *albicans* wild-type strain SC5314 [32], GFP-expressing *C*. *albicans* strain (*Ca*GFP) [1], *C*. *glabrata* wild-type strain ATCC2001 [33] and *C*. *glabrata* expressing GFP (*Cg*GFP) [34] were routinely seeded in YPD medium (2% D-glucose, 1% peptone, and 0.5% yeast extract, in water) and grown overnight at 30°C (*C*. *albicans strains)* and 37°C (*C*. *glabrata* strains), respectively, in a shaking incubator. Fungal cells were reseeded 1:50 in fresh YPD medium, grown until they reach the mid-log phase (OD_600_ ~ 1) followed by harvesting in HBSS.

Killing of *C*. *albicans* wild-type strain SC5314 or *C*. *glabrata* wild-type strain ATCC2001 was performed after staining with Cell Tracker^™^Green (Invitrogen) for 1 hour using 0.1% thimerosal (Sigma-Aldrich) at 37°C for 1 hour and followed by extensively washing with HBSS.

### *Staphylococcus aureus* strains and culture

*S*. *aureus* wild-type strain ATCC25923 and GFP-expressing *S*. *aureus* (*Sa*GFP) [35, 36] were cultivated overnight in lysogeny broth (LB) medium (10 g/l tryptone, 5 g/l yeast extract, 10 g/l sodium chloride, pH 7) at 37°C in a shaking incubator. In case of *Sa*GFP the lysogeny broth medium was supplemented with 10 μg/ml chloramphenicol. Bacterial cells were reseeded 1:100 in fresh lysogeny broth medium, grown until they reach the mid-log phase (OD_600_ = 0.6–0.7) followed by harvesting in HBSS containing 10% heat-inactivated human serum (hiHS, Sigma-Aldrich).

Killing of *S*. *aureus* wild-type strain ATCC25923 was performed after staining with Cell Tracker^™^Green for 30 minutes using 50% ethanol for four hours at 37°C and followed by extensively washing with HBSS+10%hiHS.

### Human whole-blood infection assay

This study was conducted in accordance with the Declaration of Helsinki and all protocols were approved by the Ethics Committee of the University Hospital Würzburg (permit number: 37/17). Human peripheral blood was collected from healthy donors with written informed consent. The whole-blood infection assay was performed as described previously [1]. Briefly, alive GFP-expressing C. *albicans*, *C*. *glabrata* or *S*. *aureus* cells were added to Hirudin-anti-coagulated blood (1x10^6^ fungal or bacterial cells per milliliter of blood) and incubated at 37°C on a rolling mixer for indicated time points. Treatment of blood with either HBSS or HBSS containing 10% hiHS served as mock-infected control samples. After incubation, the samples were immediately placed on ice and subjected to flow cytometric analyses.

### Flow cytometry

Analyses of immune cell populations in whole blood with regard to phagocytosis of fungal or bacterial cells were performed using differential FACS staining and subsequent measurement with a FACS Canto II (BD Bioscience). To distinguish different immune cells, whole blood was stained with mouse anti-human CD3 (clone SK7, T cells), CD19 (clone HIB19, B cells), CD56 (clone B159, NK-cells) and CD66b (clone G10F5, PMN) obtained from BD Biosciences. Monocytes were labeled with mouse anti-human CD14 antibody (clone 47-3D6, Abcam). The stained samples were treated with FACS Lysing solution (BD Biosciences) that lyses erythrocytes while preserving and fixing leukocytes, followed by washing and harvesting cells in CellWASH solution (BD Biosciences).

Detailed data analysis including gating of relevant immune cell populations was performed using FlowJo 7.6.4 software. The strategy used to evaluate the association of microorganisms to immune cells in human blood was shown for *Ca*GFP in Hünniger *et al*. [1].

### Propidium iodide staining and imaging

To evaluate the amount of dead *Ca*GFP, *Cg*GFP or *Sa*GFP cells in a fresh induction culture, microorganisms were stained with 2.5 ng/ml of propidium iodide (Sigma-Aldrich). Propidium iodide (PI) is widely used as a vital dye that is not permeant to live cells, but labels the nucleus in dying cells, which lack an intact plasma membrane. Thimerosal-killed Cell Tracker^™^Green-labeled *C*. *albicans* or *C*. *glabrata* and ethanol-killed Cell Tracker^™^Green-labeled *S*. *aureus* cells, respectively, served as controls for a positive PI staining. Fluorescence images of *C*. *albicans* and *C*. *glabrata* samples were acquired with the Zeiss AxioObserver Z.1 microscope using a 40x objective (EC Plan-Neofluar 40x/0,75) and processed using the ZEN 2.3 software (Carl Zeiss). Images of *S*. *aureus* samples were acquired with the LSM 780 confocal microscope using a 63x objective (Plan-Apochromat 63x/1.4 Oil DIC) and processed using the ZEN 2.3 software.

### Automated image analysis

The microscopy images consist of two different channels: the green fluorescence channel with GFP-labeled pathogens and the red fluorescence channel for pathogen live/dead PI staining (S27 Fig of S1 File). The microscopy images with either *C*. *albicans* cells or *C*. *glabrata* cells were analyzed and counted by the automated segmentation algorithm that is integrated within the framework AMIT (Algorithm for Migration and Interaction Tracking) [14, 37, 38]. In brief, the green fluorescence images were segmented and cell clusters were split into their constituent cells using the implemented cluster splitting algorithm that is based on the method of Farhan *et al*. [39]. Here, a cluster was split after identifying the concave contour regions between the borders of neighboring convex cells. Since the parameters of this cluster splitting method are strongly dependent on pathogen specific properties, such as the average diameter of the respective cells, we adapted these parameters individually for each type of pathogen following the guidelines given by Brandes *et al*. [14]. The respective parameter values are given in S4 Table of S1 File. In case of *C*. *albicans* cells and *C*. *glabrata* cells, we observed reproduction by budding where small outgrowths are formed. These smaller daughter cells adhere to the mother cell and are only evaluated as a new cell if they reach the size of an average cell. The cluster splitting algorithm detected cells with a large bandwidth of different area sizes. To prevent daughter cells from being counted as single cells, these were excluded by a histogram threshold method over the area sizes (see S4 Table of S1 File).

In the case of *S*. *aureus* cells with sizes of only 1 μm in diameter–corresponding to five pixels à 0.2 μm–an alternative segmentation approach was applied involving a pixel-based thresholding approach. Since the illumination was varying from image to image, handling of *S*.*aureus* images was done in the following way: Images showing local inhomogeneities with a small contrast between the cell border and the background intensity were pre-processed by a contrast enhancement, where the image intensity was stretched over the whole range of intensity values. In order to remove background noise and emphasize the cells itself, a median filter with kernel size 4 × 4 *px* was used. Finally, a global thresholding was performed on the image intensity histogram to reduce noise in the images. The number of *S*.*aureus* cells was then determined by dividing the amount of foreground pixels through the average cell size. In order to determine the total number of dead cells, the resulting segmented image was applied as a binary mask for the PI stained channel that was processed by the same three methods, *i*.*e*. thresholding, median filtering and contrast enhancement.

### Whole-blood infection modeling

In this study, we generated mathematical models with different mechanisms for immune evasion of pathogens in whole blood. These state-based models (SBMs) were derived from our previous models of whole-blood infection [1, 12]. In order to compare the dynamics of the SBMs with the time-resolved experimental measurements, we defined the five *combined units P*_*E*_, *P*_*M*_, *P*_*N*_, *P*_*A*_ and *P*_*K*_ as variables that combine specific states, which represent specific cell populations. The combined unit *P*_*E*_ is defined by

$$P_{E}\equiv P_{AE}+P_{KE}+P_{AIE}+P_{KIE}$$ (1)

and incorporates all states that represent extracellular pathogens, *i*.*e*. states for extracellular pathogens that are alive (*P*_*AE*_) or killed (*P*_*KE*_), or immune-evasive pathogens that are alive (*P*_*AIE*_) or killed (*P*_*KIE*_). Pathogens phagocytosed by PMN or monocytes are respectively recorded within the combined units

$$P_{N}\equiv \sum_{i\geq 0}\sum_{j\geq 0}(i+j)N_{i,j}$$ (2)

and

$$P_{M}\equiv \sum_{i\geq 0}\sum_{j\geq 0}(i+j)M_{i,j}.$$ (3)

Here, the states *N*_*i*,*j*_ and M_*i*,*j*_ represent neutrophils and monocytes respectively, with the number of *i* alive pathogens and *j* killed pathogens. We limit the maximal number of phagocytosed pathogen cells to nine (*i*.*e*. j<10), a value far above what has been observed in experiments [1]. The combined unit *P*_*A*_ contains all states that represent alive pathogens and is defined by

$$P_{A}\equiv P_{AE}+P_{AIE}+\sum_{i\geq 0}\sum_{j\geq 0}(M_{i,j}+N_{i,j})i,$$ (4)

whereas the combined unit of killed pathogens is given by

$$P_{K}\equiv P_{KE}+P_{KIE}+\sum_{i\geq 0}\sum_{j\geq 0}(M_{i,j}+N_{i,j})j.$$ (5)

Note that the total number of pathogens is given by *P* ≡ *P*_*E*_ + *P*_*N*_ + *P*_*M*_ and *P* ≡ *P*_*A*_ + *P*_*K*_. Biological processes that take place during the immune response to the pathogens are represented by state transitions characterized by transition rates. All SBMs contain at least four types of state transitions that are the phagocytosis by PMN or monocytes, the intracellular killing by PMN or monocytes and the extracellular killing of pathogens (see S5 Table of S1 File). The implementation of the immune-evasion mechanism of pathogens differs between the SBMs as described in the next subsection.

The SBMs were applied to simulate the immune response to 1 × 10^6^ pathogens in 1 *ml* blood containing 5 × 10^6^ PMN and 5 × 10^5^ monocytes; thus, the initial conditions of the model simulation are *P*_*A*_(*t* = 0) = 1 × 10^6^, *N*_0,0_(*t* = 0) 5 × 10^6^, *M*_0,0_(*t* = 0) 5 × 10^5^. The time-evolution of the SBMs is calculated by the random selection simulation algorithm using a time-step driven, synchronous update scheme [40] (see S1 Fig of S1 File for the flow chart of the algorithm). In this simulation algorithm, the simulation time is divided into discrete time-steps with distance Δ*t* and at each simulation time-step a transition from state *S* to state *S*′ can be performed with probability *P*_*S*→*S*′_ = *r*_*S*→*S*′_ × Δ*t*, where *r*_*S*→*S*′_ denotes the corresponding transition rate. In S5 Table of S1 File, a complete list of transition rates of the SBMs is given. Here it can be seen that the SBMs contain common transition rates for the following transitions: phagocytosis by PMN or monocytes, intracellular killing by PMN or monocytes as well as extracellular killing. The killing of pathogens in the extracellular space is caused by antimicrobial peptides released during the first-time phagocytosis of PMN. Therefore, the rate for extracellular killing *κ*_*EK*_(*t*) is defined by

$$\kappa_{EK}(t=n\Delta t)={\overset{-}{\kappa }}_{EK}\sum_{m=0}^{n}\frac{N_{PE}(t=m\Delta t)}{N_{0,0}(0)}e^{-\gamma \Delta t(n-m)},$$ (6)

and describes the time-dependent effect of antimicrobial peptides by the constant rate ${\overset{-}{\kappa }}_{EK}$, a half-life time characterized by the rate *γ* and the number of first-phagocytosis events (*N*_*PE*_) per PMN (*N*_0,0_).

For further information about the model structure, the simulation algorithm as well as the estimation of transition rate values using the global optimization algorithm *Simulated Annealing* we refer to our previous studies by Hünniger *et al*. [1] and Lehnert *et al*. [12].

In order to investigate the unresolved mechanism of immune evasion of pathogens, we generated various SBMs that differ in the implementation of the rate for the acquisition of an immune-evasive state. We implemented two models with solely one immune-evasion mechanism, two models without an evasion mechanism but instead with either populations of alive and killed pre-existing evasive cells or with only alive pre-existing cells. We refer to these models as base models in the following. Additionally, we implemented four so called combined models that are composed of at least two base models and thereby contain more than one option for the acquisition of immune evasion. The representation of the different immune-evasion mechanisms in the SBMs is described in the following section and an overview is given in Table 1. The source code of all models is available in the Immune evasion repository at https://asbdata.hki-jena.de/LehnertEtAl_PLOS/.

### Modeling immune evasion of pathogens

The population of pathogens that can evade the immune response in whole blood was first described in our studies by Hünniger *et al*. [1] and Lehnert *et al*. [12]. In these studies, experimental measurements revealed that at four hours post infection alive and extracellular pathogens are present. These observations gave rise to define the transitions

$$P_{AE}\to P_{AIE}$$ (7)

and

$$P_{KE}\to P_{KIE},$$ (8)

where extracellular pathogens that are alive (*P*_*AE*_) or killed (*P*_*KE*_) can become immune-evasive pathogens that are alive (*P*_*AIE*_) or killed (*P*_*KIE*_) and thereby cannot be phagocytosed by immune cells and/or killed. The corresponding transition rate was defined by

ρ=constant, (9)

since there were no information about this mechanism known and we consequently assumed that the process occurs spontaneously (see Fig 1A). In the following, we refer to this model as *spon-IE model*.

In the subsequent study by Prauße *et al*. [2], we took advantage of the strength of mathematical modeling and implemented the *PMNmed-IE model* to test the hypothesis that the acquisition of immune evasion is mediated by PMN. More precisely, we speculated that during first-time phagocytosis by PMNs not only molecules causing extracellular killing will be released but also specific molecules that aims at protecting pathogens against the immune system (see Fig 1B). In the PMNmed-IE model, we replaced the constant transition rate of the spon-IE model and defined the time-dependent rate for the acquisition of immune evasion by

$$\rho (t=n\Delta t)=\overset{-}{\rho }\sum_{m=0}^{n}\frac{N_{PE}(t=m\Delta t)}{N_{0,0}(0)}e^{-\gamma_{IE}\Delta t(n-m)}.$$ (10)

Similar to the time-dependent rate for extracellular killing *κ*_*EK*_(*t*) (see Eq 6), this rate represents the time-dependent effect of specific molecules that were secreted upon the first-time phagocytosis events by PMN (*P*_*PE*_). The effect of these molecules is specified by the constants $\overset{-}{\rho }$ and *γ*_*IE*_ and decreases exponentially.

In addition, we investigated the hypothesis that immune-evasive cells do not develop during immune response to pathogens but instead are present before infection as pre-existing immune-evasive cells (see Fig 1C). To this end, we implemented two models that do not contain the transition from the extracellular state to the immune-evasive state (see Eqs 7 and 8), but rather contain constant populations of immune-evasive cells that are present during the infection time. In the pre-IE model, we assume that both alive and killed evasive pathogens are present before infection and therefore set the number of alive and killed immune-evasive cells at time point of infection *t* = 0 to *P*_*AIE*_ > 0 and *P*_*KIE*_ > 0. The *alivePre-IE model* contains exclusively alive evasive pathogens (see Fig 1D) and therefore, we set *P*_*AIE*_ > 0 and *P*_*KIE*_ > 0 at time point of infection *t* = 0.

Thereby, the number of *a priori* unknown transition rates decreased in these two models in comparison to the spon-IE and the PMNmed-IE models. However, the numbers of alive and killed immune-evasive cells at time *t* = 0 *min* are additional *a priori* unknown values and have to be estimated in the course of the parameter estimation. The total number of parameters of each SBM that has to be estimated is given in Table 1. As a starting value of evasive cells, the algorithm randomly chooses a value in the range of the mean ± standard deviation of experimentally measured pathogens that are free in extracellular space and not associated with immune cells at four hours post infection (see Fig 2B).

Furthermore, we implemented four SBMs with more than one of the above described evasion mechanisms in order to investigate the possibility that immune evasion of pathogens could be developed in various ways. As given in Table 1, we implemented not only three SBMs with two evasion mechanisms, *i*.*e*. the spon-PMNmed-IE model, the spon-alivePre-IE model and the PMNmed-alivePre-IE model, but also one SBM with three immune-evasion mechanisms, the spon-PMNmed-alivePre-IE model.

### Model parameter estimation by Simulated Annealing

The estimation of optimal values for the transition rates of the different immune evasion mechanism was rformedperformed by the global optimization method *Simulated Annealing* based on the *Metropolis Monte Carlo* scheme [41]. Starting with a random position in the parameter space, the algorithm performs a random walk and decides for the next step based on a score of the deviations between the model simulation with model parameters $\vec{p}$ and experimental data, *i*.*e*. the weighted sum of least-square errors (LSE)

$$E[\vec{p}[=\sum_{c}\omega_{c}\cdot \varepsilon_{c}[\vec{p]}.$$ (11)

Here, *ω*_*c*_ is defined as the specific weight of the LSE

$$\varepsilon_{c}[\vec{p}]=\frac{1}{2}\sum_{k}(x_{k,c}^{dat}-x_{k,c}^{sim}[\vec{p}])²,$$ (12)

which is defined as the sum of the squared difference between the experimental data ($x_{k,c}^{exp}$) and the comparable simulated data ($x_{k,c}^{sim}$), which forms the combined unit c, for each time point k. The definition of each combined unit is provided in the methods section “Whole-Blood Infection Modeling”. The weights used for the infection scenarios with *C*. *albicans* and *C*. *glabrata* were taken over from our previous studies [1, 2]. For infection with *S*. *aureus*, we used the same weights as for *C*. *albicans* infection.

In course of the random walk through the parameter space, the next step will be performed by accepting a parameter set $\vec{p\prime }$ with a smaller $E[\vec{p\prime }]$ in comparison to $E[\vec{p}]$, the score of the current parameter set $\vec{p}$, *i*.*e*. $\Delta Ε={Ε}^{\prime }[\vec{p}]-Ε[\vec{p}]<0$. However, the Metropolis Monte Carlo scheme enables that also parameter sets with a larger LSE are accepted with a certain probability, the acceptance probability, which is dependent on the Boltzmann distribution and decreases with the increasing number of fitting steps *f*. The acceptance function plays the role of the “system’s temperature”, which decreases during the annealing process. The flowchart in S2 Fig of S1 File shows the algorithm. For a detailed description of this algorithm we refer to our previous studies of Hünniger *et al*. [1] and Lehnert *et al*. [12]. The source code of this algorithm is available in the Immune evasion repository at https://asbdata.hki-jena.de/LehnertEtAl_PLOS/.

We repeated the whole fitting procedure to increase the statistical robustness of this procedure. Furthermore, we performed this procedure for different system sizes by starting with *P* = 100 *cells*, *M*_0,0_ = 50 *cells* and *N*_0,0_ = 500 *cells* and stepwise increasing the number of cells by a factor of ten, while keeping the ratio of pathogens and immune cells constant. The optimal parameter set of a system size was used as starting point for the fitting procedure of the next larger system size until the system size of 1 *ml* blood with *P = 10*^*6*^ *cells*, *M*_0,0_ = 5 · 10^5^ *cells* and *M*_0,0_ = 5 · 10^6^ *cells* is reached.

### Model comparison based on information criterion

In order to compare the different mathematical models for the immune evasion of pathogens, we applied the Akaike information criterion (AIC) [13]. This score aims to rank the different models by including not only the agreement of the model kinetics with the experimental data, like the LSE (see Eq 12), but also by incorporating the complexity of the models regarding the number of parameters. For least-square estimation, the *AIC* is defined by

$$AIC=n\;\text{ln}\left(\frac{{\hat{\sigma }}^{2}}{n}\right)+2K$$ (13)

as described by Burnham et al. [42]. Here, *K* denotes the number of parameters that is directly proportional to the penalty defined in the second term. The first term of Eq 13 represents the penalty by the number of independent experimental data points *n* and by the deviation of the model and the experimental data in terms of the sum of squared residuals

$$\hat{\sigma }={\sum_{i=1}^{i=n}\left(x_{i\;}^{exp}-x_{i}^{sim}[{\vec{p}}_{opt}]\right)}^{2}.$$ (14)

This variable is calculated by the sum of the squared differences between experimental data (*x*^*exp*^) and simulated data ($x^{sim}[{\vec{p}}_{opt}]$) resulting from simulating the model with the optimal parameter set ${\vec{p}}_{opt}$ at each of the *ith* experimentally measured data points (*i* = 1, 2, 3, …, 27). The corrected version of the AIC, the

$$AIC_{c}=AIC+\frac{2K(K+1)}{n-K-1}$$ (15)

must be applied if *n/K* ≤ 40, *i*.*e*. the number of model parameters is large relative to the number of experimental data points *n* [42]. For each of the *m* models, the *AIC*_*C*_ value was calculated and, subsequently, the relative measure

$${\Delta AIC}_{c}^{i}=AIC_{c}^{i}-AIC_{c}^{min}$$ (16)

was used to rank the models. Here, $\Delta AIC_{c}^{i}$ denotes the relative *AIC*_*C*_ difference of the *ith* model with $AIC_{c}^{i}$ to the model with the smallest *AIC*_*C*_ ($AIC_{c}^{min}$). Based on this difference the models were categorized by means of the following assessment guidelines [43]

$$C(\Delta AIC_{c}^{i})=\{\begin{array}{cc} \Delta AIC_{c}^{i}\leq 2: & model\;i\;has\;substantial\;support,\\ 4\leq \Delta AIC_{c}^{i}\leq 7: & model\;i\;has\;considerably\;less\;support,\\ \Delta AIC_{c}^{i}>10: & model\;i\;has\;essentially\;no\;support. \end{array}$$

### Statistical analysis

Statistical analysis was performed by applying the following steps. First, we test whether the underlying data is normally distributed using the Shapiro-Wilk test. If the data was normally distributed, the unpaired t-test was used to test for significant differences. If the data was not normally distributed the Mann-Whitney U test (Wilcoxon rank-sum test) was applied to test unpaired samples for significant differences. If comparisons were made between several data sets, a multiple comparison correction (Bonferroni’s correction) was performed afterwards. The corrected P-value is given by *P*′. Significance is shown as *P′ < 0.05, **P′ < 0.001, ***P′ < 0.001.

## Supporting information

S1 File (PDF)
