# Supplementary material for: Comparative assessment of immune evasion mechanisms in human whole-blood infection assays by a systems biology approach
Source: PLoS One. 2021 Apr 1;16(4):e0249372. doi: 10.1371/journal.pone.0249372 (PMC8016326; doi:10.1371/journal.pone.0249372)
Supplement: S1 File — (PDF) [file pone.0249372.s001.pdf]

Assessment of immune evasion mechanisms in human whole-blood infection assays by a systems biology approach

Short Title: Assessing pathogen immune evasion in human whole-blood

Authors

Teresa Lehnert[1,2], Maria T.E. Prauße[1,3], Kerstin Hünniger[4,5], Jan-Philipp Praetorius[1,3], Oliver Kurzai[2,4,5], Marc Thilo Figge[1,2,3,*]

Affiliations

1 Applied Systems Biology, Leibniz Institute for Natural Product Research Infection Biology, Hans Knöll Institute (HKI), Jena, Germany

2 Center for Sepsis Control and Care (CSCC), Jena University Hospital, Jena.

3 Institute of Microbiology, Faculty of Biological Sciences, Friedrich Schiller University Jena, Jena

4 Fungal Septomics, Leibniz Institute for Natural Product Research Infection Biology, Hans Knöll Institute (HKI), Jena, Germany

5 Institute of Hygiene and Microbiology, University of Würzburg, Würzburg, Germany

* Corresponding author: Marc Thilo Figge, email: thilo.figge@leibniz-hki.de

Supporting Figures

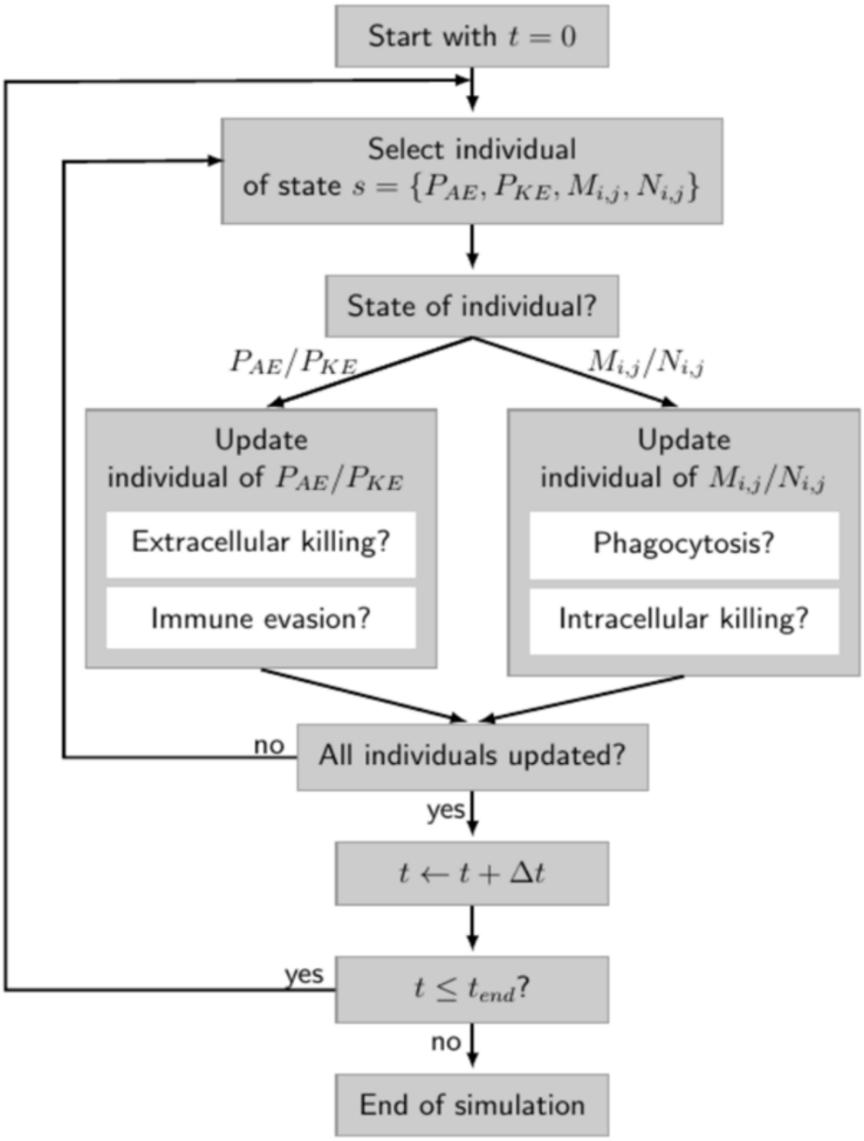

S1 Fig. Flowchart of the algorithm used for generating simulations with the state-based whole-blood infection model. For each time step Δt during the simulation time $t = 0, \dots, t_{end}$ the system's development is implemented in such a way that each individual is addressed and updated in respect to their current state. Depending on the individual being in state s it is updated to state s' by testing its transition *i.e.* testing the associated rate $r^{s \to s'}$ whether the

probability $P_{s \to s'} = r^{s \to s'} \Delta t$ is met. This is done for each individual for each time step. These individuals comprise alive extracellular pathogens (P_{AE}) and killed extracellular pathogens (P_{KE}) but also monocytes $M_{i,j}$ and PMN $N_{i,j}$ containing i alive pathogens and j killed pathogens. All extracellular pathogens are tested for immune evasion whereas alive extracellular pathogens are also tested for extracellular killing. Individuals which are part of the states $M_{i,j}$ and $N_{i,j}$ are tested whether they phagocytose pathogens or kill them intracellularly. Further explanations concerning transitions are given in the Methods section. The flowchart is adapted from Lehnert et al. [14].

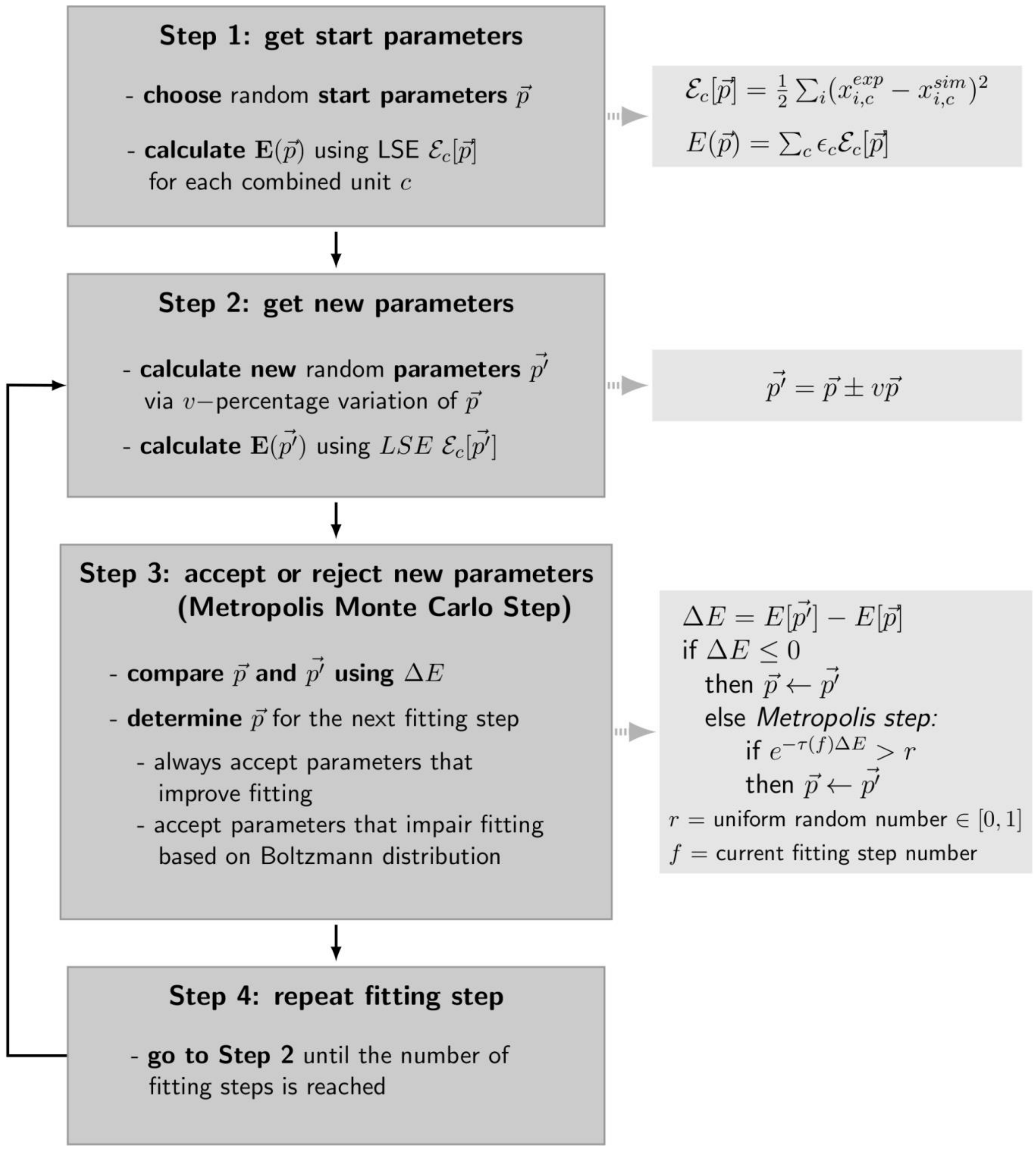

S2 Fig. Flowchart of the global optimization method Simulated Annealing based on the Metropolis Monte Carlo Scheme. This algorithm is explained in further details in the Methods Section „Model Parameter Estimation by Simulated Annealing“. This flowchart is adapted from Lehnert et al. [14].

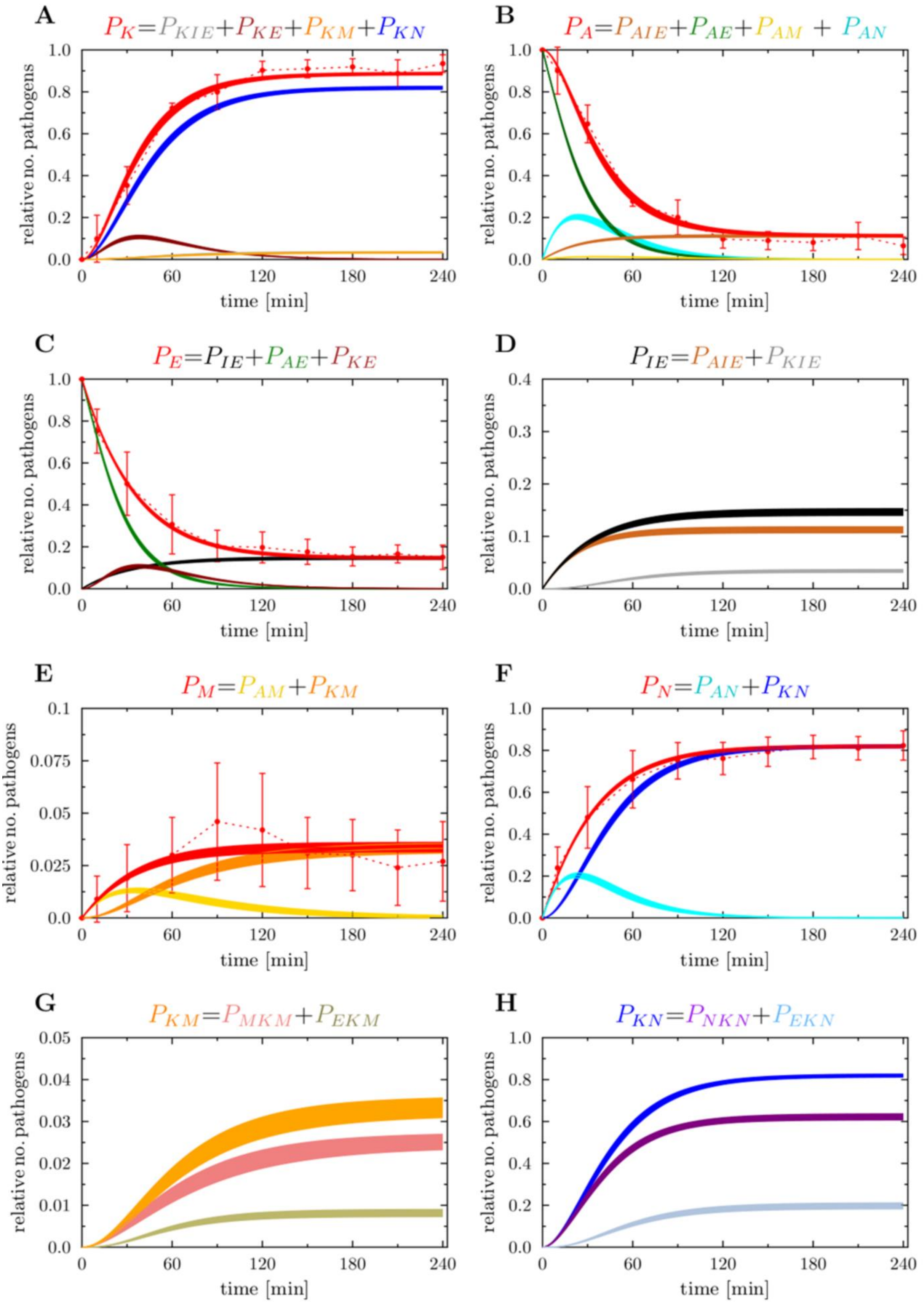

A
$P_K = P_{KIE} + P_{KE} + P_{KM} + P_{KN}$
B
$P_A = P_{AIE} + P_{AE} + P_{AM} + P_{AN}$
C
$P_E = P_{IE} + P_{AE} + P_{KE}$
D
$P_{IE} = P_{AIE} + P_{KIE}$
E
$P_M = P_{AM} + P_{KM}$
F
$P_N = P_{AN} + P_{KN}$
G
$P_{KM} = P_{MKM} + P_{EKM}$
H
$P_{KN} = P_{NKN} + P_{EKN}$
relative no. pathogens
time [min]

S3 Fig. Result of the spon-IE model simulation generated by optimized transition rates for a *C. albicans* infection. Red dotted lines correspond to the experimental data acquired from whole-blood infection assays with *C. albicans*. Solid lines correspond to the simulated data. The thickness of the solid lines represents the mean $\pm$ standard deviation obtained by 50 simulations with transition rate values that were randomly sampled within their corresponding standard deviation. (A) Kinetics of simulated killed pathogens (P_K) (solid, red line) compared to the experimentally measured killed cells (dotted, red line) by survival assays. The combined unit P_K comprises killed immune-evasive cells (P_{KIE}), killed extracellular cells (P_{KE}), killed cells within monocytes (P_{KM}) and killed cells within PMN (P_{KN}). (B) Kinetics of simulated alive pathogens (P_A) (solid, red line) compared to experimentally measured alive cells (dotted, red line) by survivalassays. The combined unit P_A comprises alive immune-evasive cells (P_{AIE}), alive extracellular cells (P_{AE}), alive cells within monocytes (P_{AM}) and alive cells associated with PMN (P_{AN}). (C) Kinetics of simulated extracellular cells (P_E) (solid, red line) compared to the experimental data (dotted, red line) acquired via flow cytometry analysis. (D) Kinetics of simulated immune-evasive cells (P_{IE}) (solid, black line). (E) Kinetics of simulated cells associated with monocytes (P_M) (solid, red line) compared to the experimental data (dotted, red line). (F) Kinetics of simulated cells associated with PMN (P_N) (solid, red line) compared to the experimental data (dotted, red line). (G) Kinetics if simulated cells which were killed intracellularly by monocytes (P_{MKM}) and those which were extracellularly killed and later phagocytosed by monocytes (P_{EKM}). (H) Kinetics if simulated cells which were killed intracellularly by PMN (P_{NKN}) and those which were extracellularly killed and later phagocytosed by PMN (P_{EKN}).

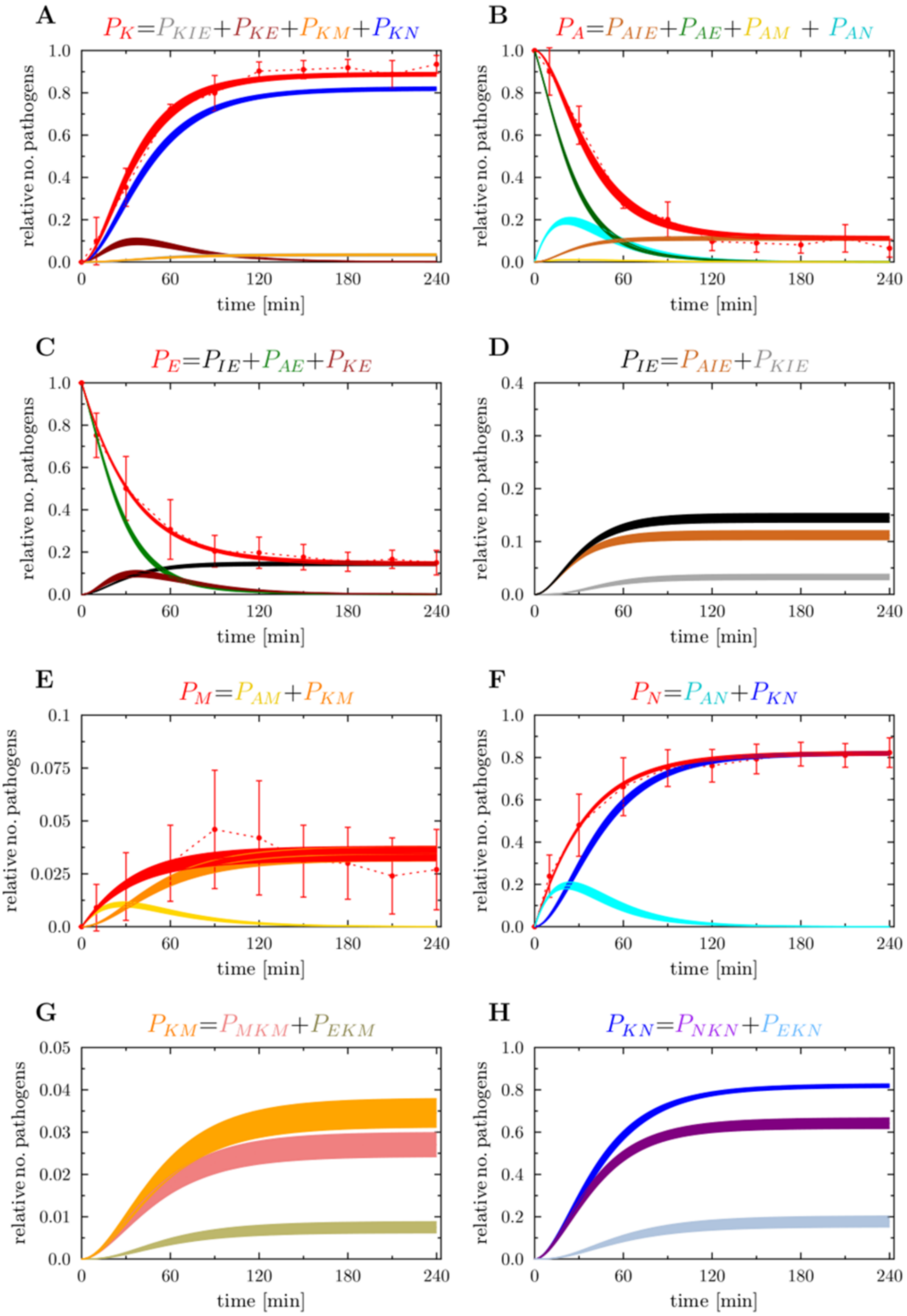

S4 Fig. Result of the PMNmed-IE model simulation generated by optimized transition rates for a *C. albicans* infection. See caption of S3 Fig for the description of the subfigures.

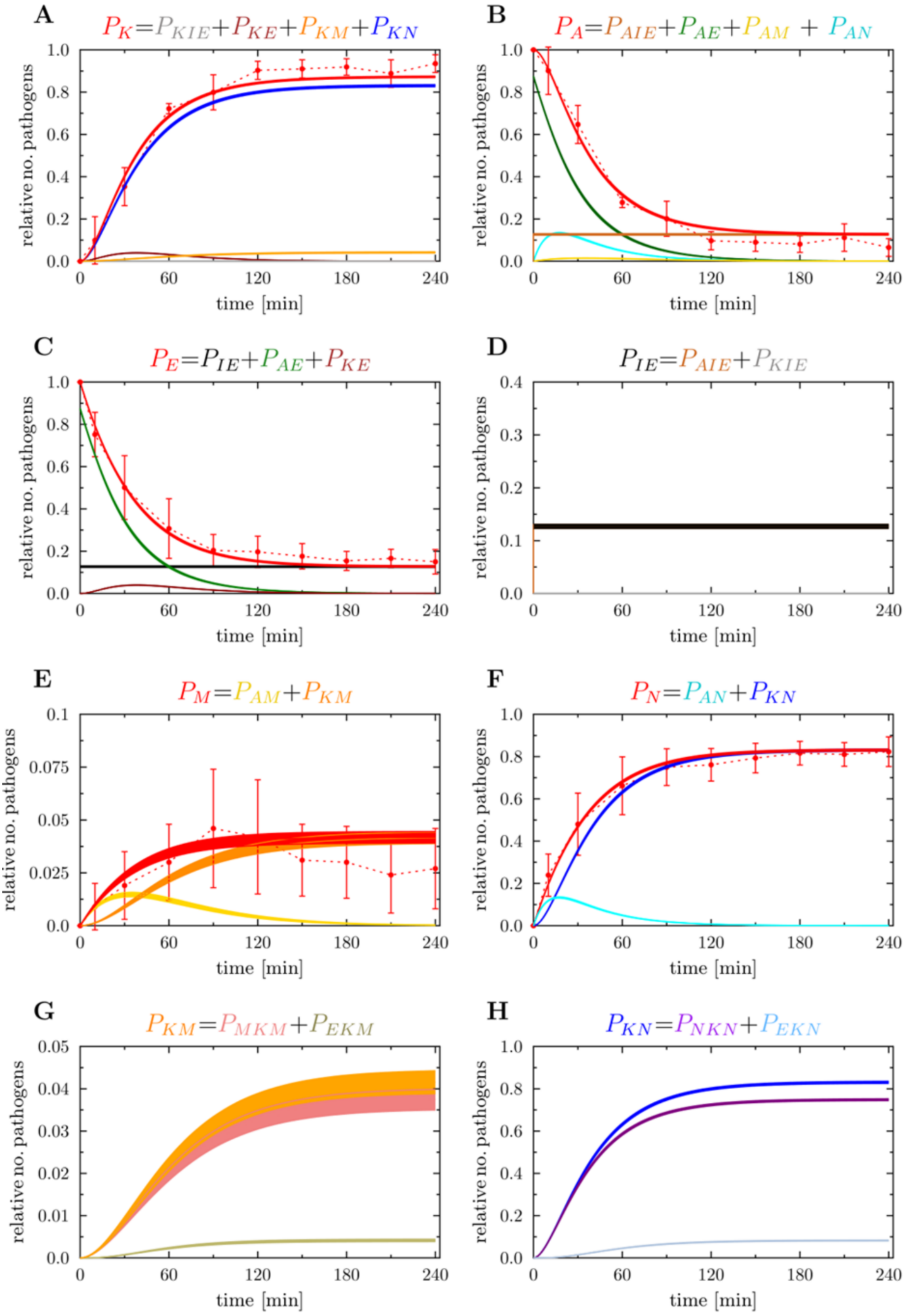

S5 Fig. Result of the alivePre-IE model simulation generated by optimized transition rates for a *C. albicans* infection. See caption of S3 Fig for the description of the subfigures.

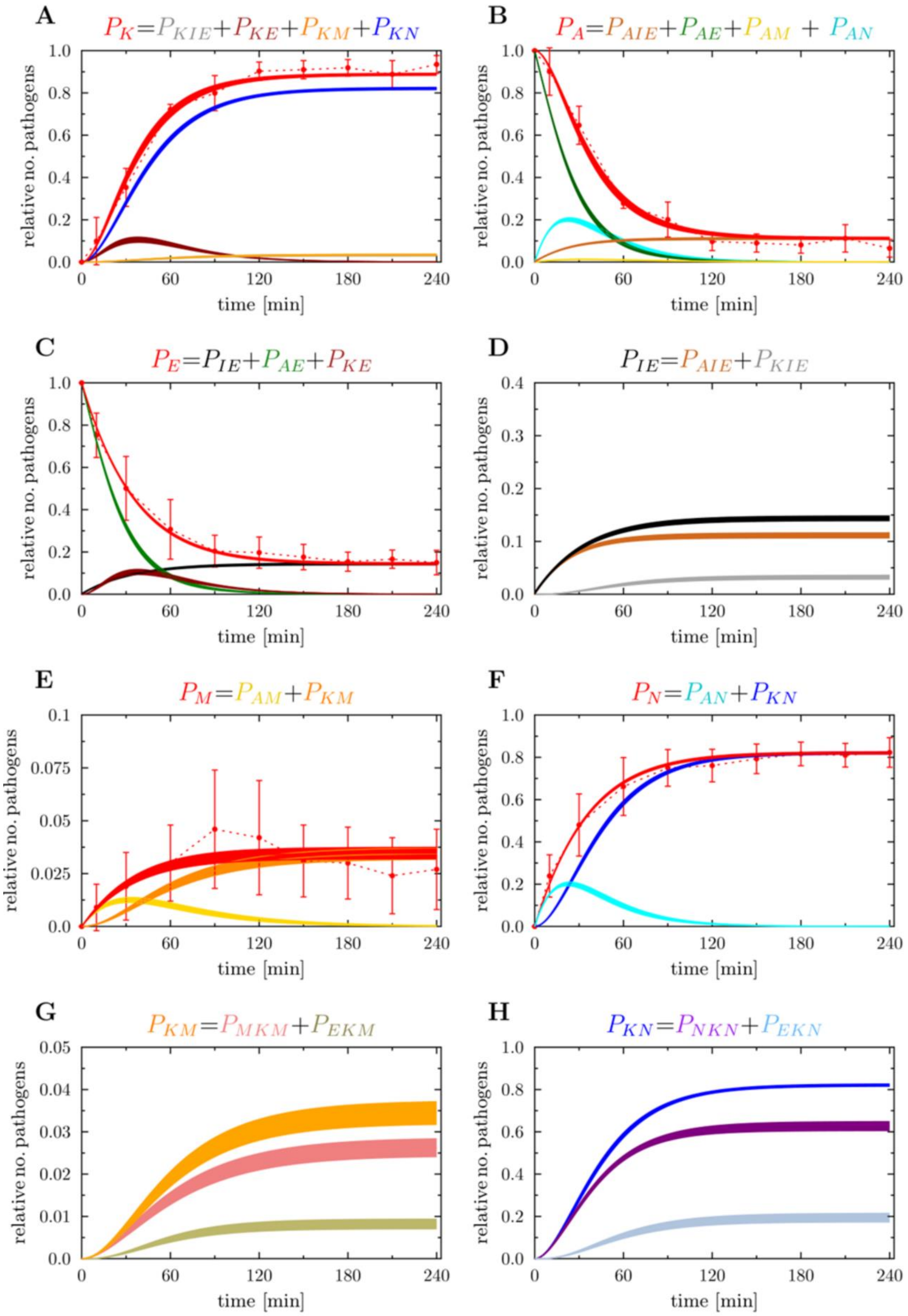

S6 Fig. Result of the spon-alivePre-IE model simulation generated by optimized transition rates for a *C. albicans* infection. See caption of S3 Fig for the description of the subfigures.

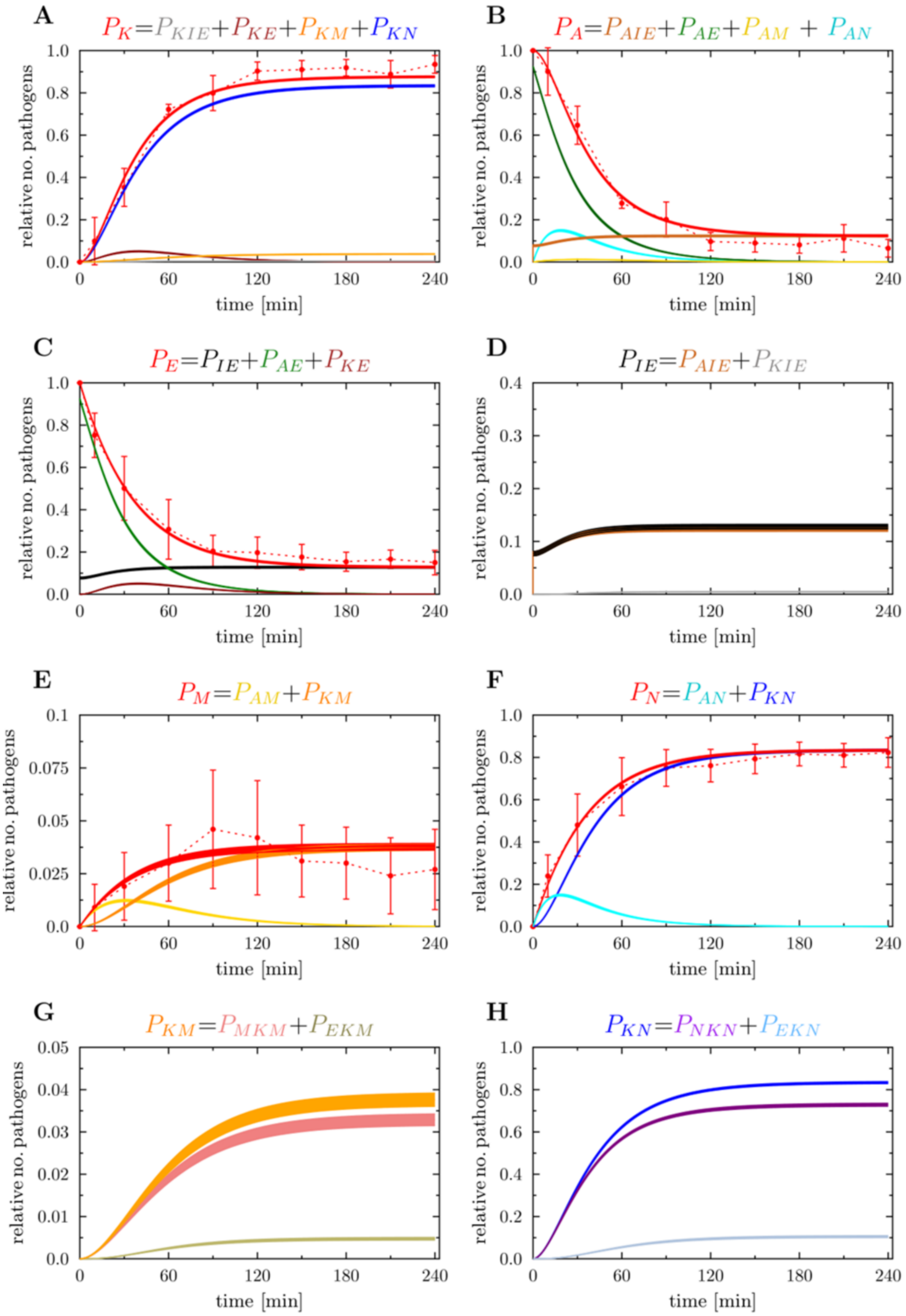

S7 Fig. Result of the PMNmed-alivePre-IE model simulation generated by optimized transition rates for a *C. albicans* infection. See caption of S3 Fig for the description of the subfigures.

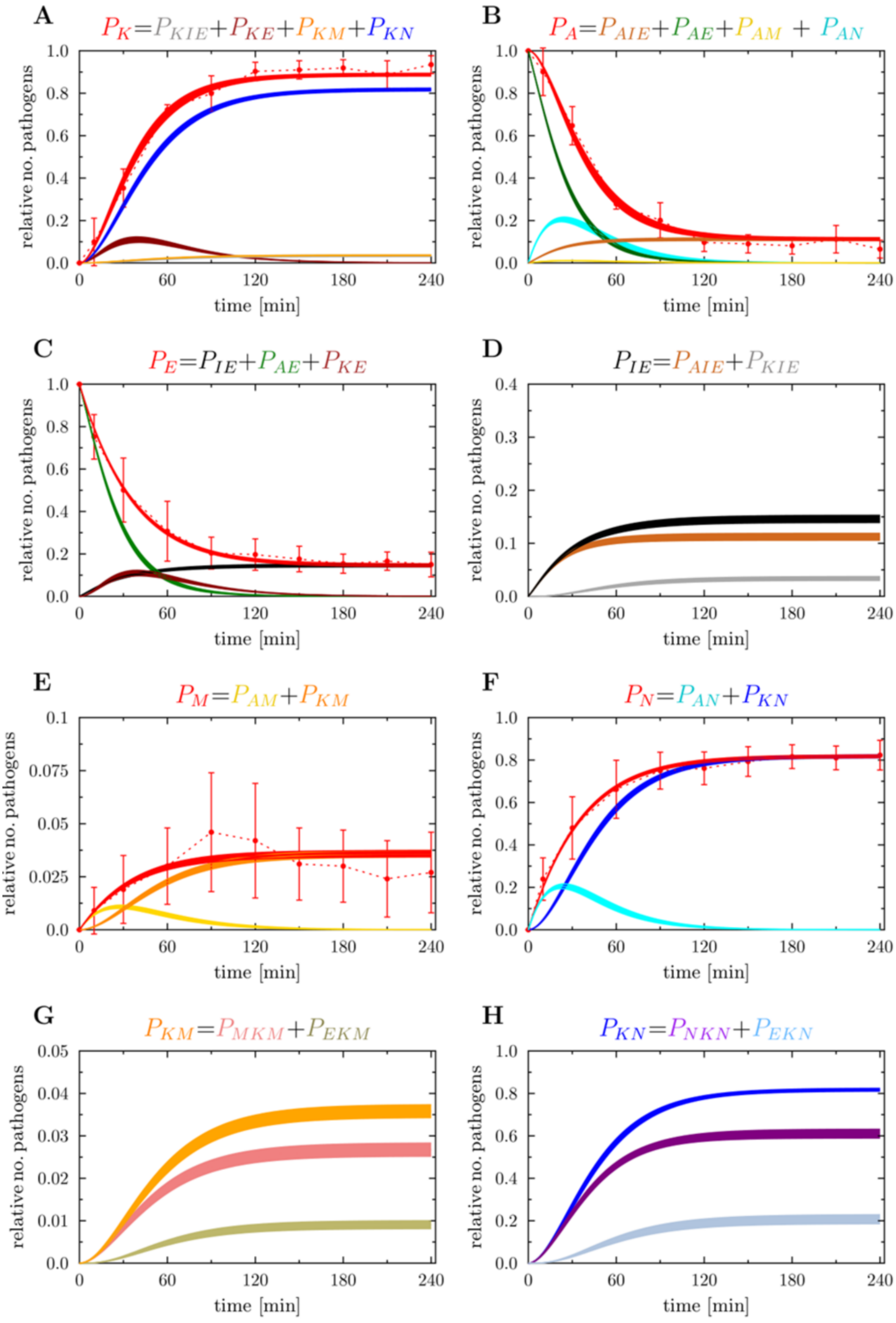

S8 Fig. Result of the spon-PMNmed-IE model simulation generated by optimized transition rates for a *C. albicans* infection. See caption of S3 Fig for the description of the subfigures.

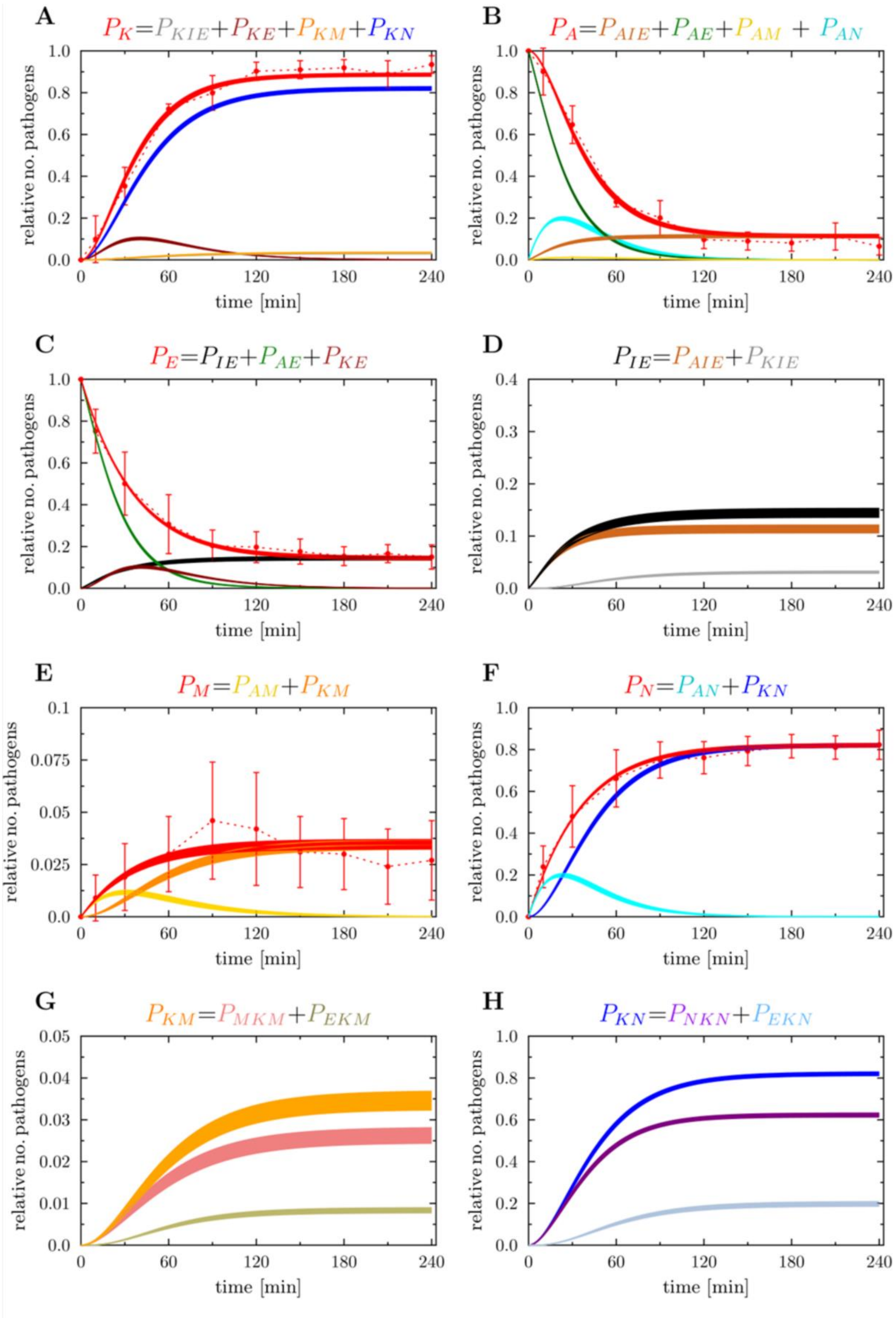

S9 Fig. Result of the spon-PMNmed-alivePre-IE model simulation generated by optimized transition rates for a *C. albicans* infection. See caption of S3 Fig for the description of the subfigures.

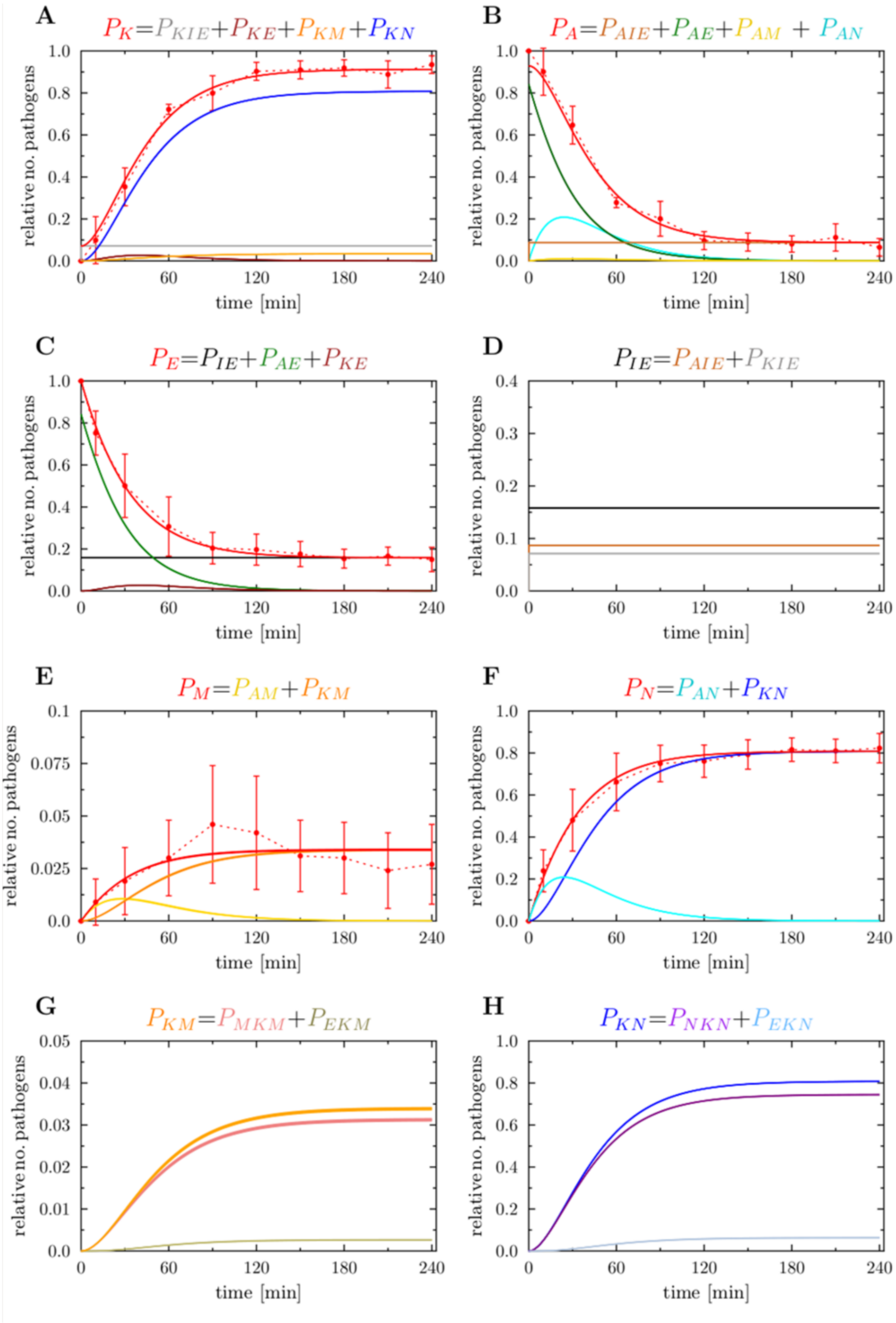

S10 Fig. Result of the pre-IE model simulation generated by optimized transition rates for a *C. albicans* infection. See caption of S3 Fig for the description of the subfigures.

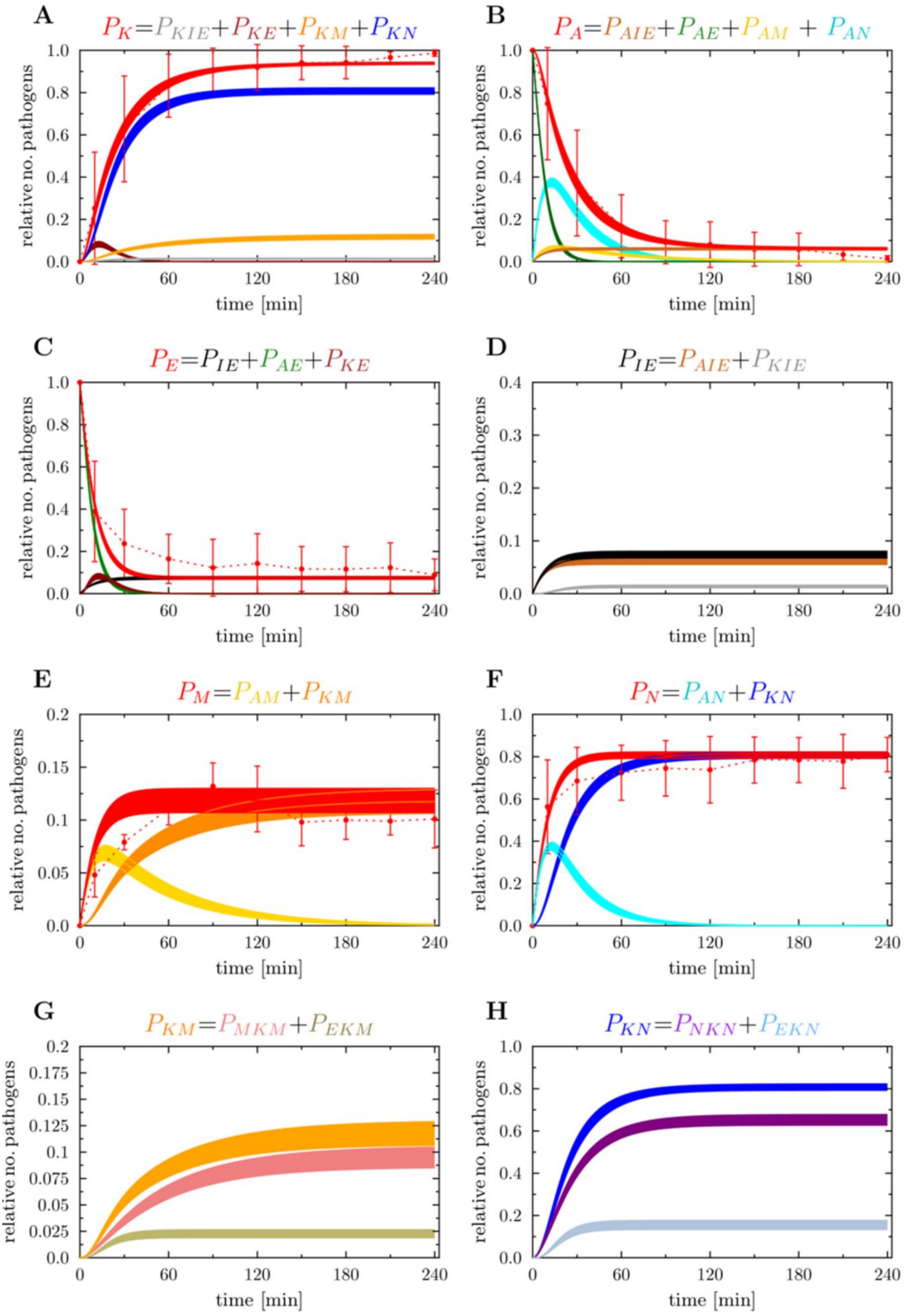

A
$P_K = P_{KIE} + P_{KE} + P_{KM} + P_{KN}$
B
$P_A = P_{AIE} + P_{AE} + P_{AM} + P_{AN}$
C
$P_E = P_{IE} + P_{AE} + P_{KE}$
D
$P_{IE} = P_{AIE} + P_{KIE}$
E
$P_M = P_{AM} + P_{KM}$
F
$P_N = P_{AN} + P_{KN}$
G
$P_{KM} = P_{MKM} + P_{EKM}$
H
$P_{KN} = P_{NKN} + P_{EKN}$
relative no. pathogens
time [min]

S11 Fig. Result of the spon-IE model simulation generated by optimized transition rates for a *C. glabrata* infection. Red dotted lines correspond to the experimental data acquired from whole-blood infection assays with *C. glabrata*. Solid lines correspond to the simulated data. The thickness of the solid lines represents the mean $\pm$ standard deviation obtained by 50 simulations with transition rate values that were randomly sampled within their corresponding standard deviation. (A) Kinetics of simulated killed pathogens (P_K) (solid, red line) compared to the experimentally measured killed cells (dotted, red line) by survival assays. The combined unit P_K comprises killed immune-evasive cells (P_{KIE}), killed extracellular cells (P_{KE}), killed cells associated with monocytes (P_{KM}) and killed cells associated with PMN (P_{KN}). (B) Kinetics of simulated alive pathogens (P_A) (solid, red line) compared to experimentally measured alive cells (dotted, red line) by survival assays. The combined unit P_A comprises alive immune-evasive cells (P_{AIE}), alive extracellular cells (P_{AE}), alive cells associated with monocytes (P_{AM}) and alive cells associated with PMN (P_{AN}). (C) Kinetics of simulated extracellular cells (P_E) (solid, red line) compared to the experimental data (dotted, red line) acquired via flow cytometry analysis. (D) Kinetics of simulated immune-evasive cells (P_{IE}) (solid, black line). (E) Kinetics of simulated cells associated with monocytes (P_M) (solid, red line) compared to the experimental data (dotted, red line). (F) Kinetics of simulated cells associated with PMN (P_N) (solid, red line) compared to the experimental data (dotted, red line). (G) Kinetics if simulated cells which were killed intracellularly by monocytes (P_{MKM}) and those which were extracellularly killed and later phagocytosed by monocytes (P_{EKM}). (H) Kinetics if simulated cells which were killed intracellularly by PMN (P_{NKN}) and those which were extracellularly killed and later phagocytosed by PMN (P_{EKN}).

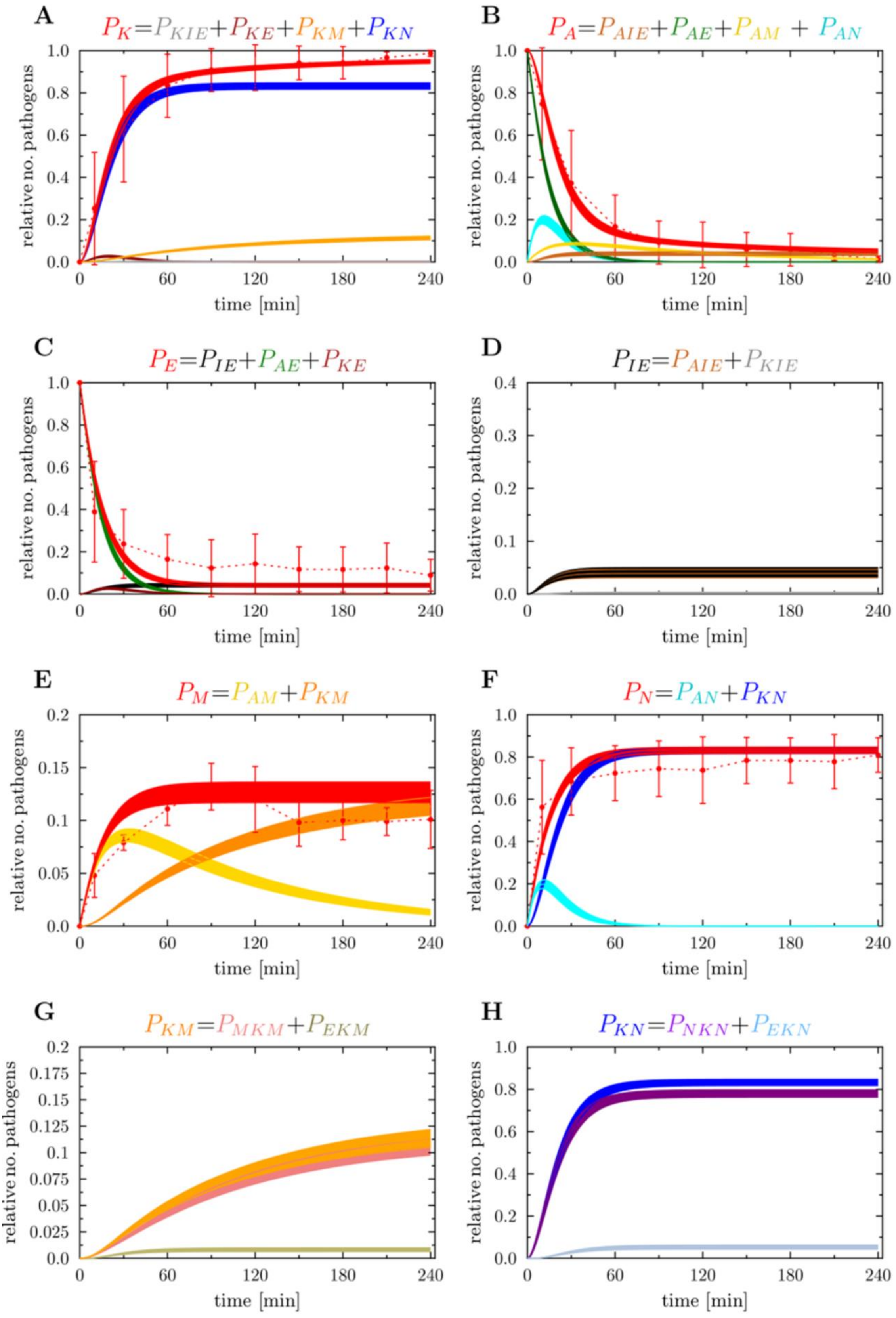

S12 Fig. Result of the PMNmed-IE model simulation generated by optimized transition rates for a *C. glabrata* infection. See caption of S11 Fig for the description of the subfigures.

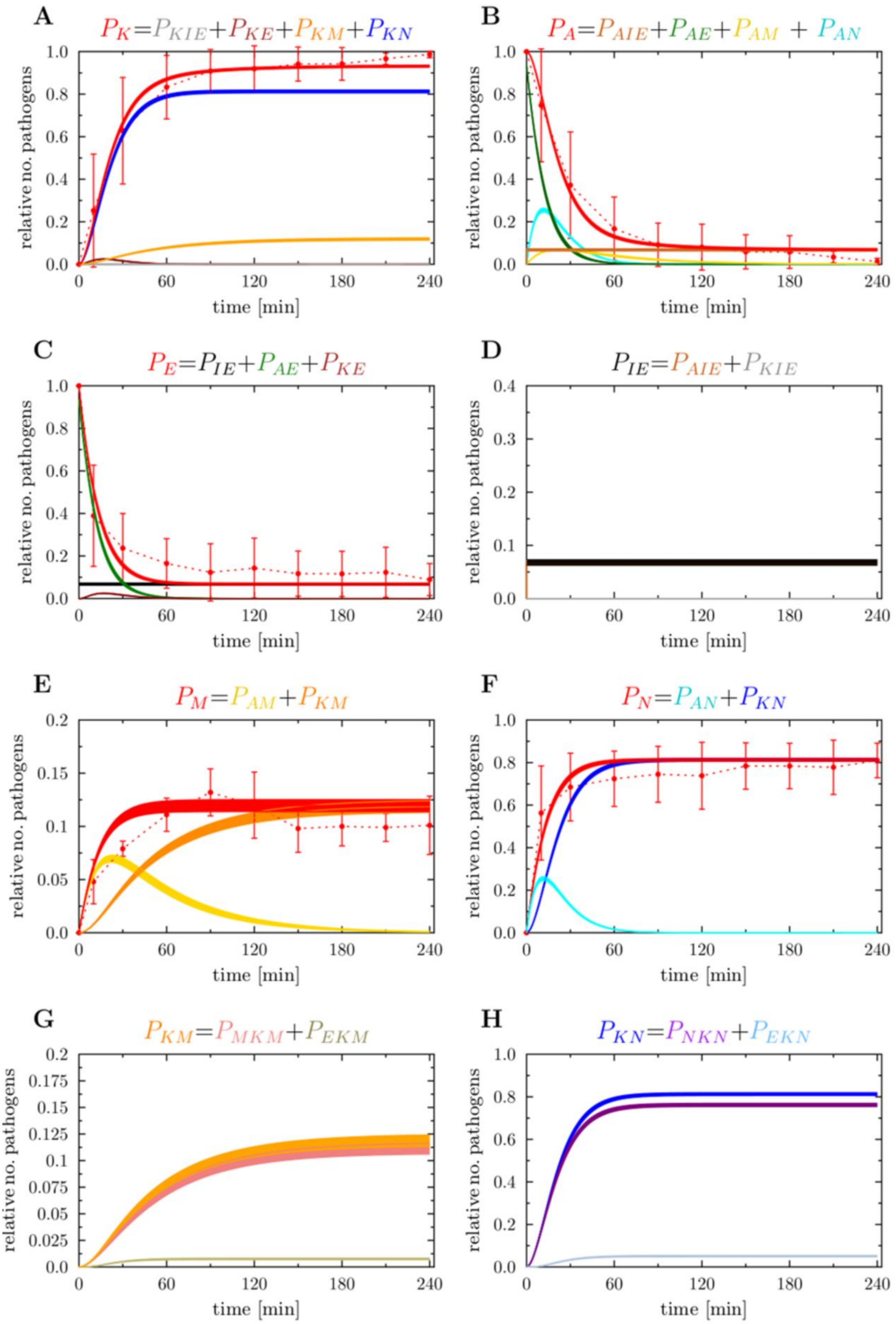

S13 Fig. Result of the alivePre-IE model simulation generated by optimized transition rates for a *C. glabrata* infection. See caption of S11 Fig for the description of the subfigures.

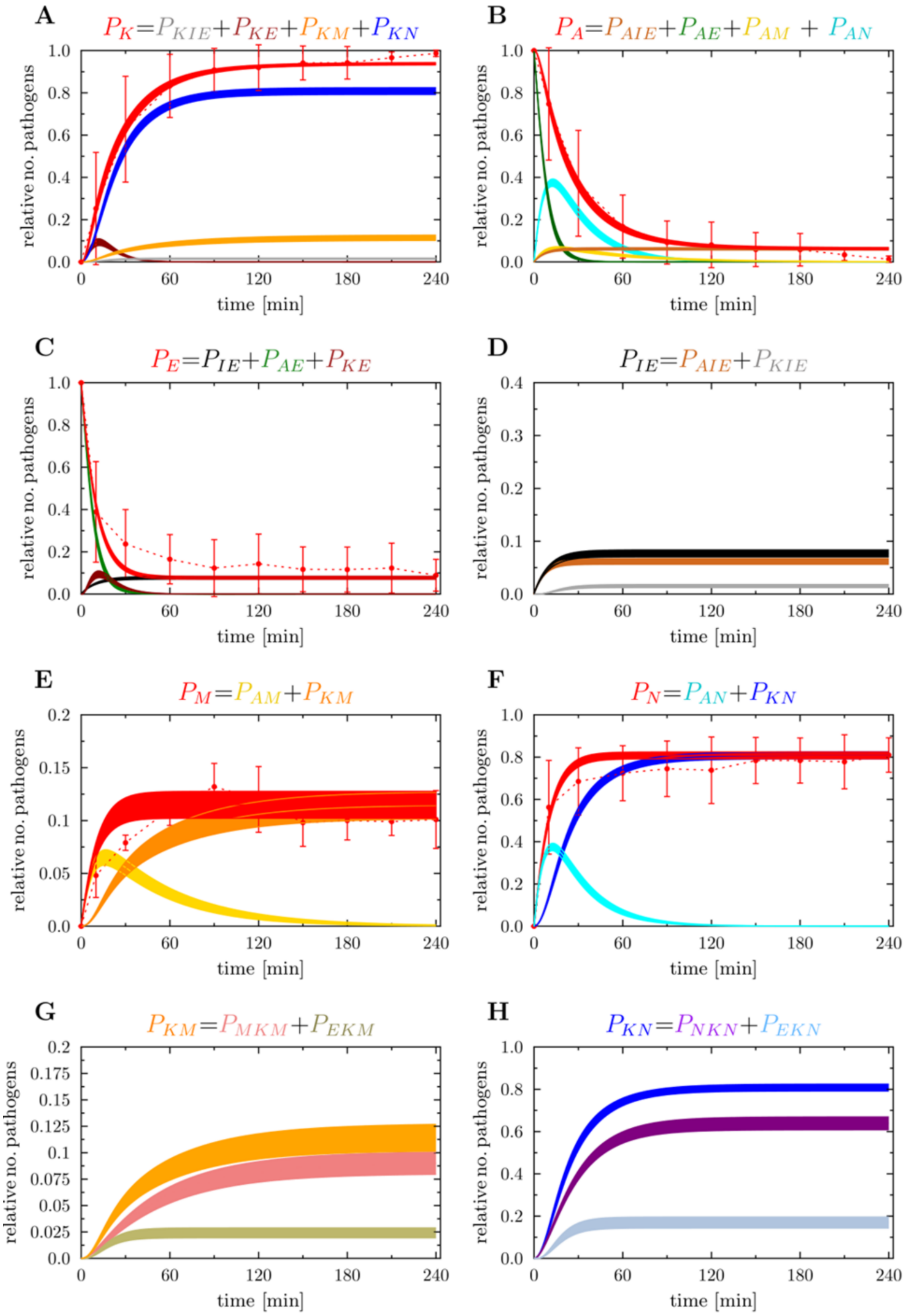

S14 Fig. Result of the spon-alivePre-IE model simulation generated by optimized transition rates for a *C. glabrata* infection. See caption of S11 Fig for the description of the subfigures.

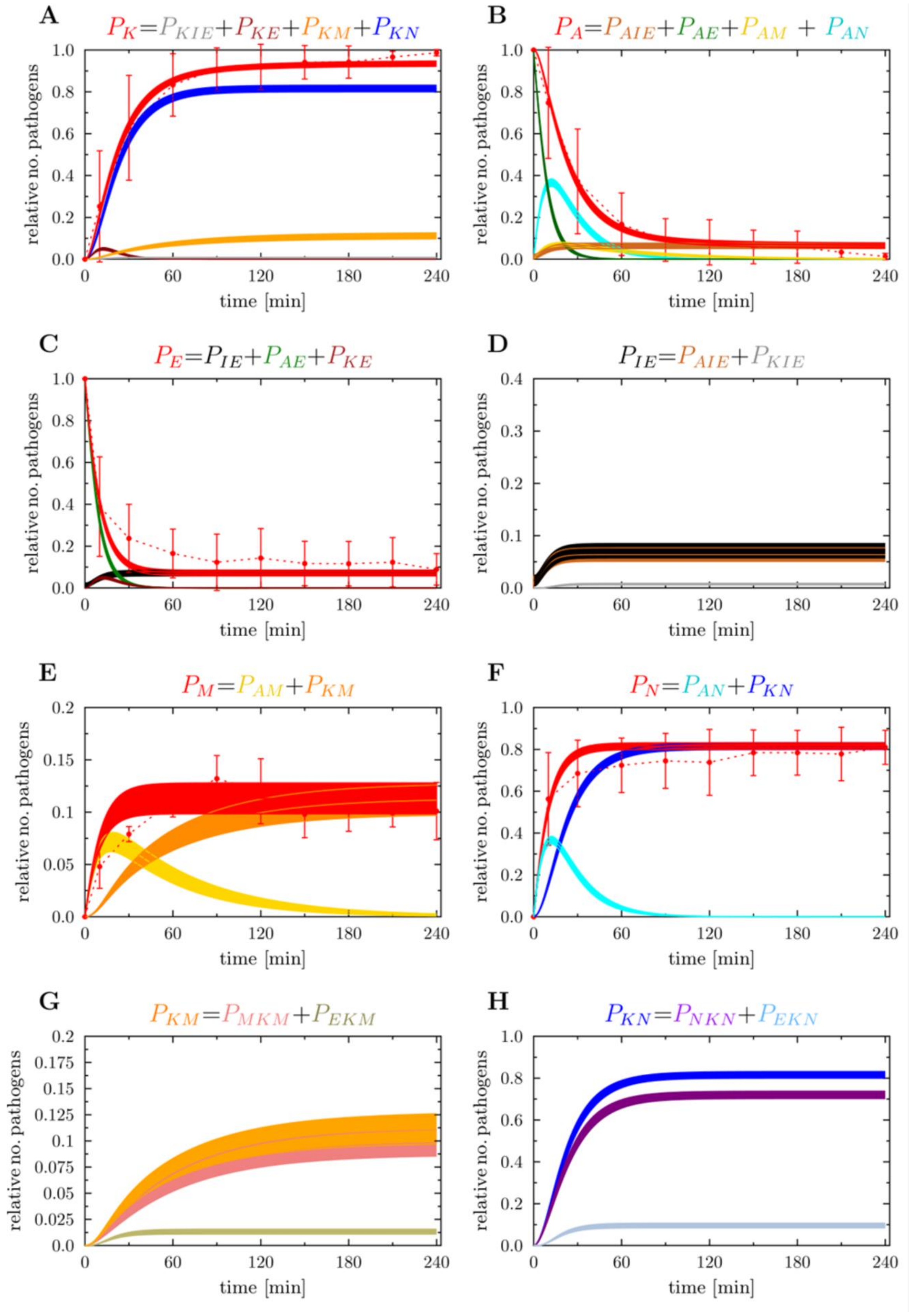

S15 Fig. Result of the PMNmed-alivePre-IE model simulation generated by optimized transition rates for a *C. glabrata* infection. See caption of S11 Fig for the description of the subfigures.

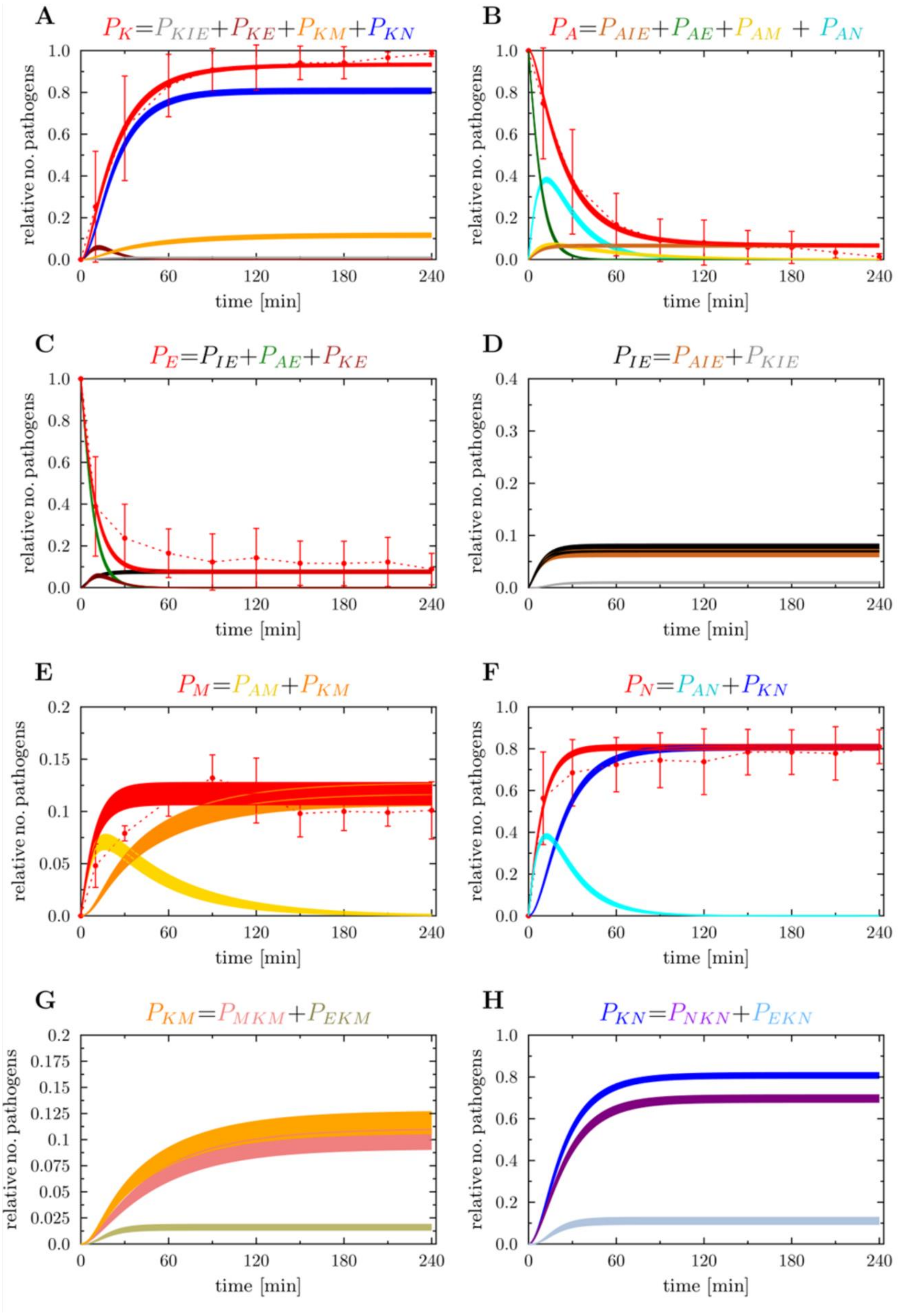

S16 Fig. Result of the spon-PMNmed-IE model simulation generated by optimized transition rates for a *C. glabrata* infection. See caption of S11 Fig for the description of the subfigures.

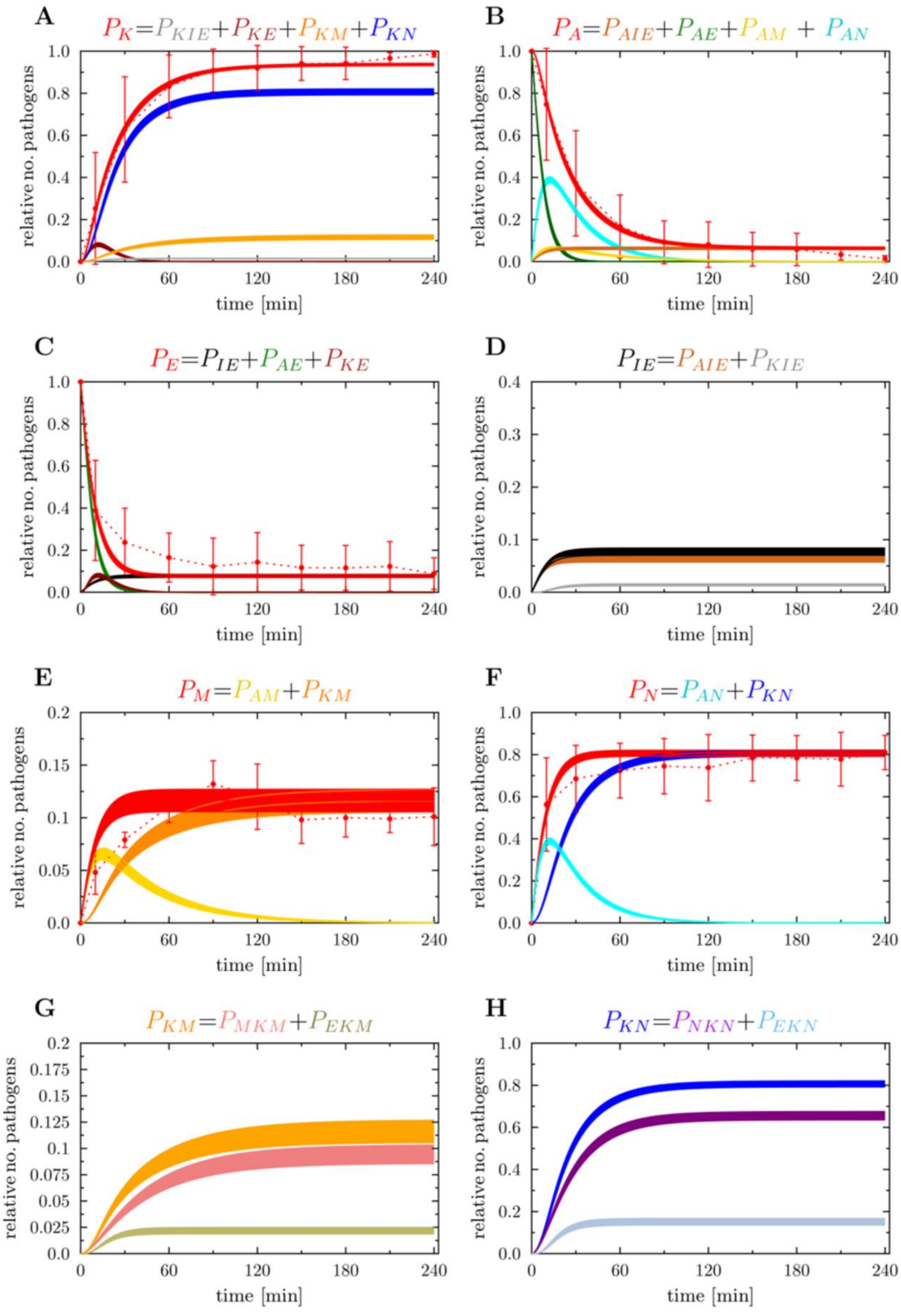

S17 Fig. Result of the spon-PMNmed-alivePre-IE model simulation generated by optimized transition rates for a *C. glabrata* infection. See caption of S11 Fig for the description of the subfigures.

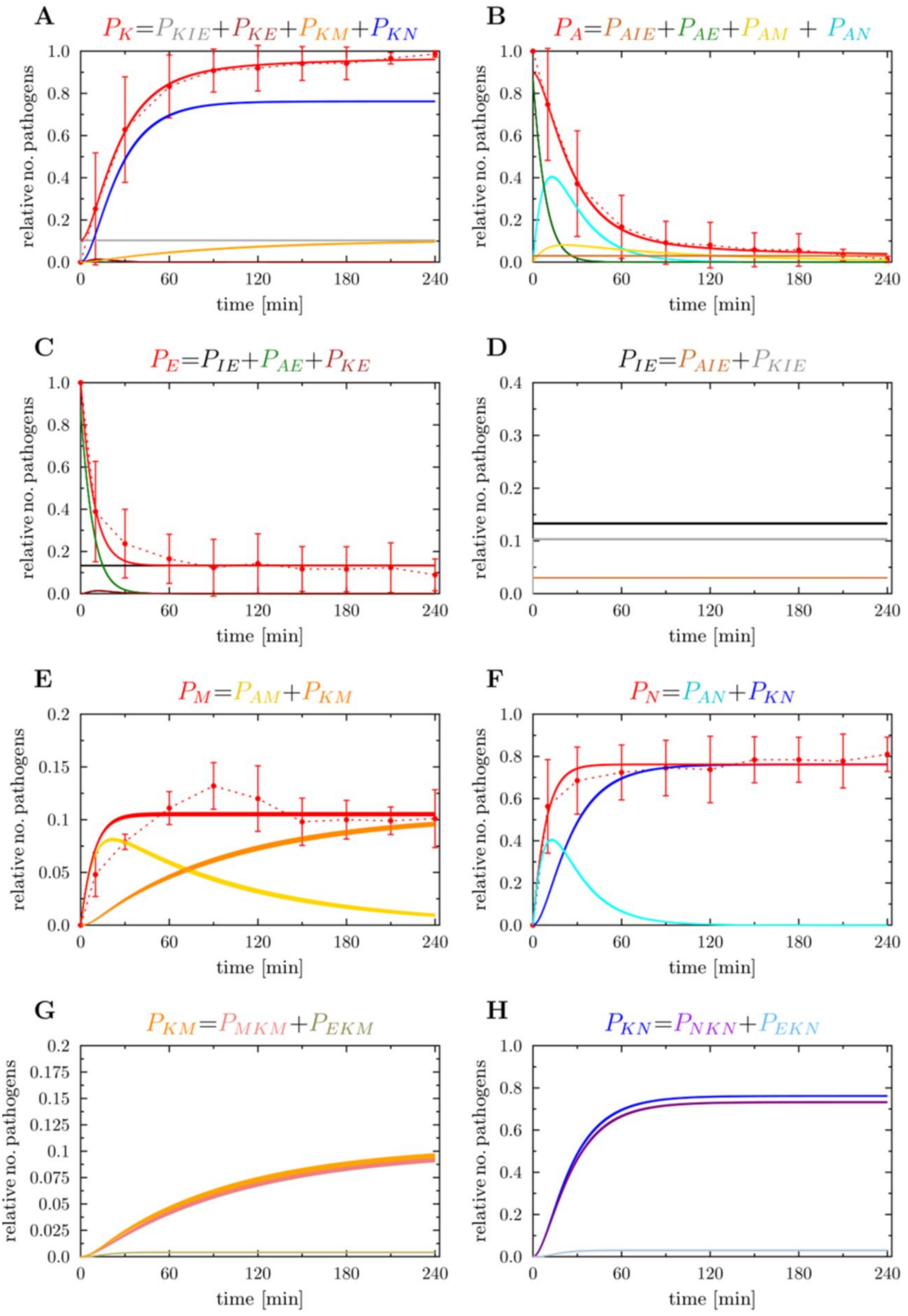

S18 Fig. Result of the pre-IE model simulation generated by optimized transition rates for a *C. glabrata* infection. See caption of S11 Fig for the description of the subfigures.

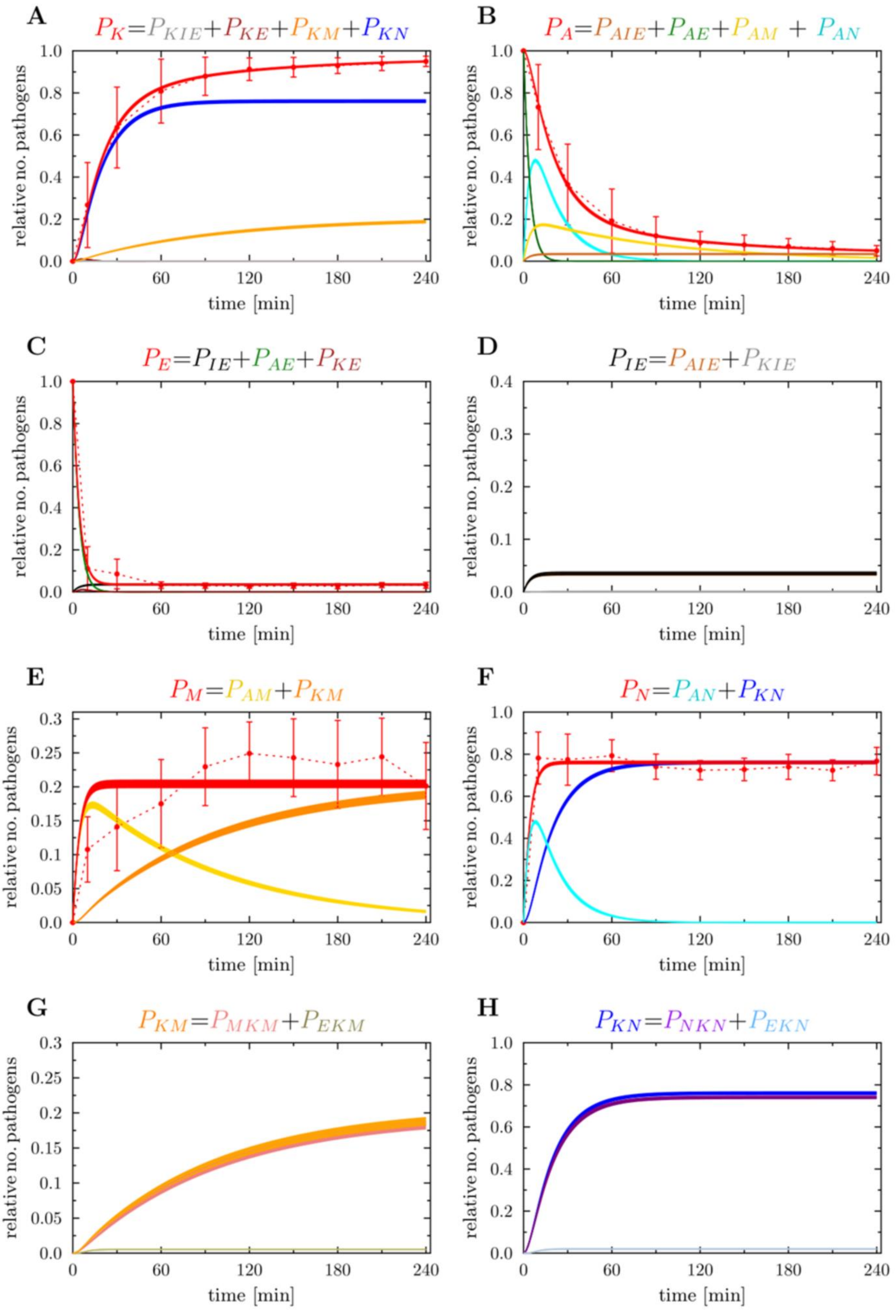
A
P_K=P_KIE+P_KE+P_KM+P_KN
B
P_A=P_AIE+P_AE+P_AM + P_AN
C
P_E=P_IE+P_AE+P_KE
D
P_IE=P_AIE+P_KIE
E
P_M=P_AM+P_KM
F
P_N=P_AN+P_KN
G
P_KM=P_MKM+P_EKM
H
P_KN=P_NKN+P_EKN
relative no. pathogens
time [min]

S19 Fig. Result of the spon-IE model simulation generated by optimized transition rates for a *S. aureus* infection. Red dotted lines correspond to the experimental data acquired from whole-blood infection assays with *S. aureus*. Solid lines correspond to the simulated data. The thickness of the solid lines represents the mean $\pm$ standard deviation obtained by 50 simulations with transition rate values that were randomly sampled within their corresponding standard deviation. Each combined unit is depicted with its sub combined units. (A) Kinetics of simulated killed pathogens (P_K) (solid, red line) compared to the experimentally measured killed cells (dotted, red line) by survival assays. The combined unit P_K comprises killed immune-evasive cells (P_{KIE}), killed extracellular cells (P_{KE}), killed cells associated with monocytes (P_{KM}) and killed cells associated with PMN (P_{KN}). (B) Kinetics of simulated alive pathogens (P_A) (solid, red line) compared to experimentally measured alive cells (dotted, red line) by survival assays. The combined unit P_A comprises alive immune-evasive cells (P_{AIE}), alive extracellular cells (P_{AE}), alive cells associated with monocytes (P_{AM}) and alive cells associated with PMN (P_{AN}). (C) Kinetics of simulated extracellular cells (P_E) (solid, red line) compared to the experimental data (dotted, red line) acquired via flow cytometry analysis. (D) Kinetics of simulated immune-evasive cells (P_{IE}) (solid, black line). (E) Kinetics of simulated cells associated with monocytes (P_M) (solid, red line) compared to the experimental data (dotted, red line). (F) Kinetics of simulated cells associated with PMN (P_N) (solid, red line) compared to the experimental data (dotted, red line). (G) Kinetics if simulated cells which were killed intracellularly by monocytes (P_{MKM}) and those which were extracellularly killed and later phagocytosed by monocytes (P_{EKM}). (H) Kinetics if simulated cells which were killed intracellularly by PMN (P_{NKN}) and those which were extracellularly killed and later phagocytosed by PMN (P_{EKN}).

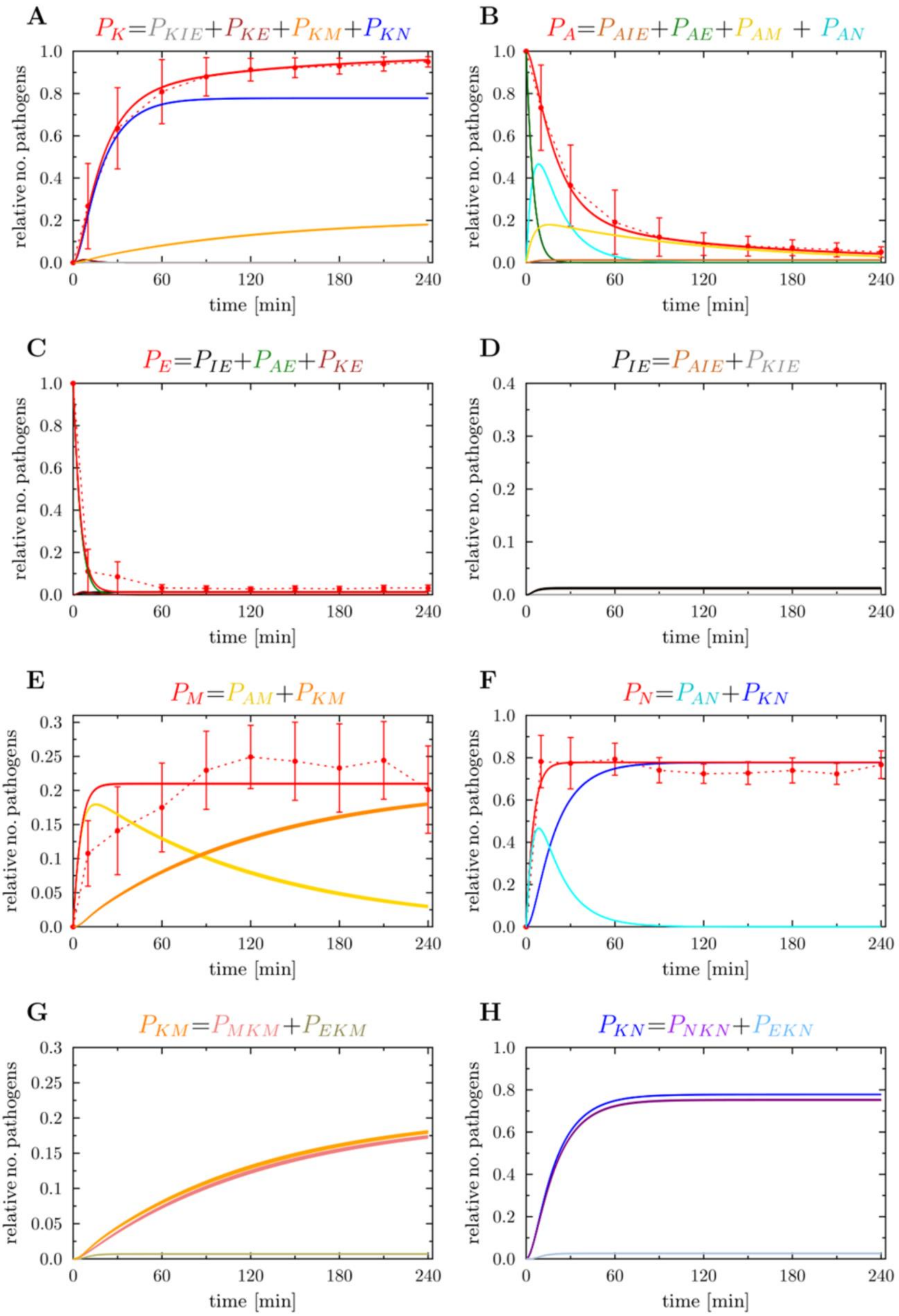

S20 Fig. Result of the PMNmed-IE model simulation generated by optimized transition rates for a *S. aureus* infection. See caption of S19 Fig for the description of the subfigures.

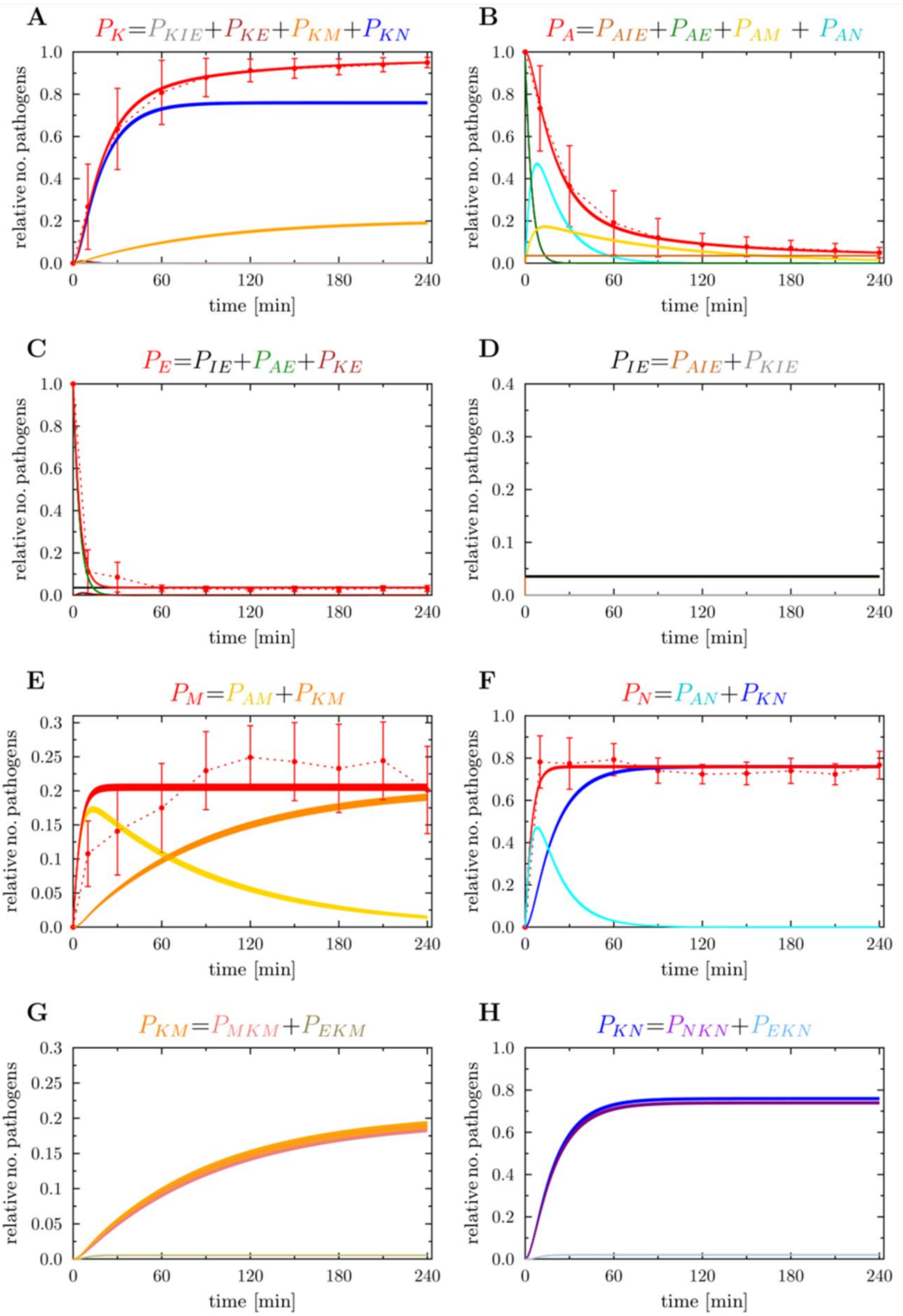

S21 Fig. Result of the alivePre-IE model simulation generated by optimized transition rates for a *S. aureus* infection. See caption of S19 Fig for the description of the subfigures.

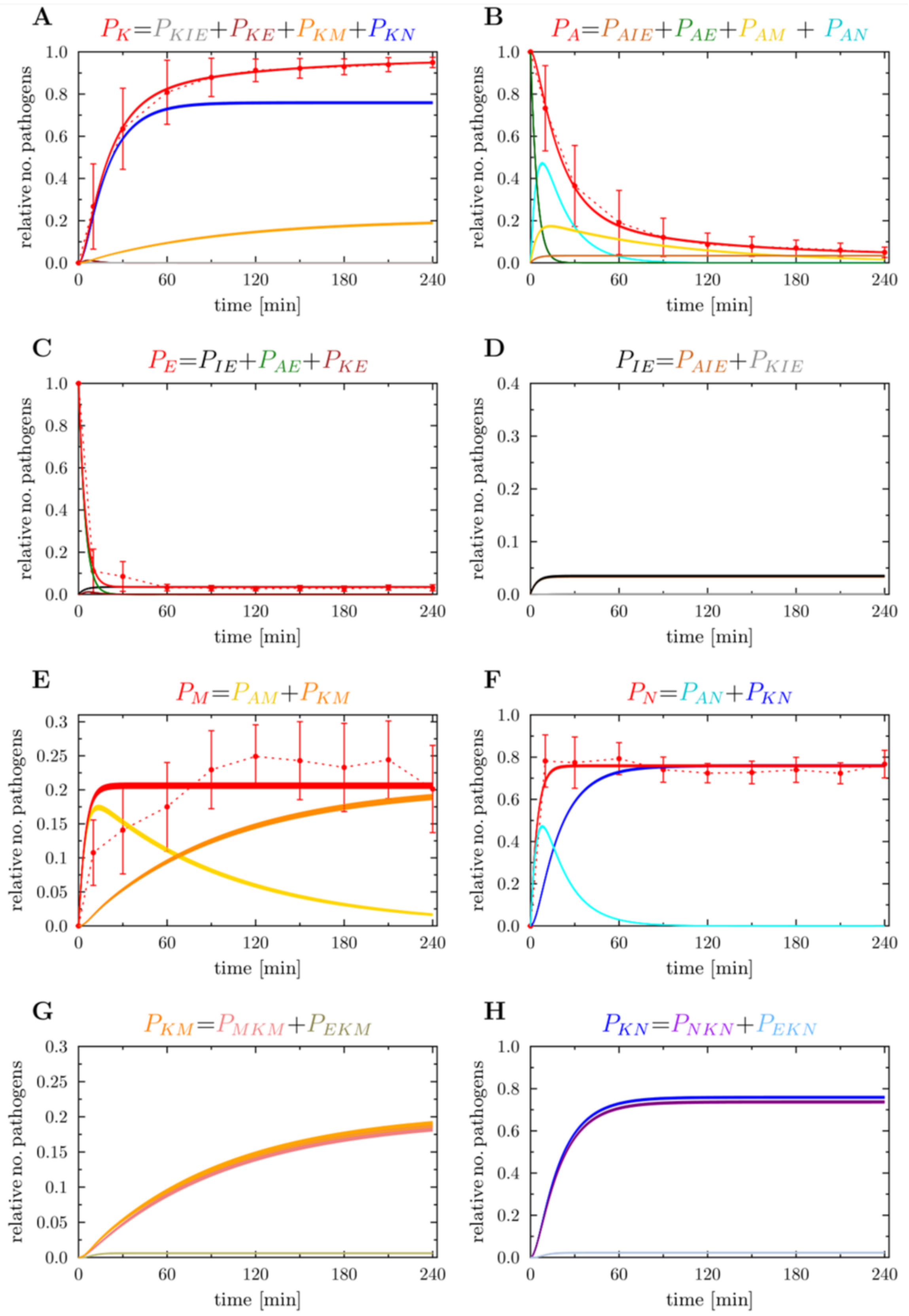

S22 Fig. Result of the spon-alivePre-IE model simulation generated by optimized transition rates for a *S. aureus* infection. See caption of S19 Fig for the description of the subfigures.

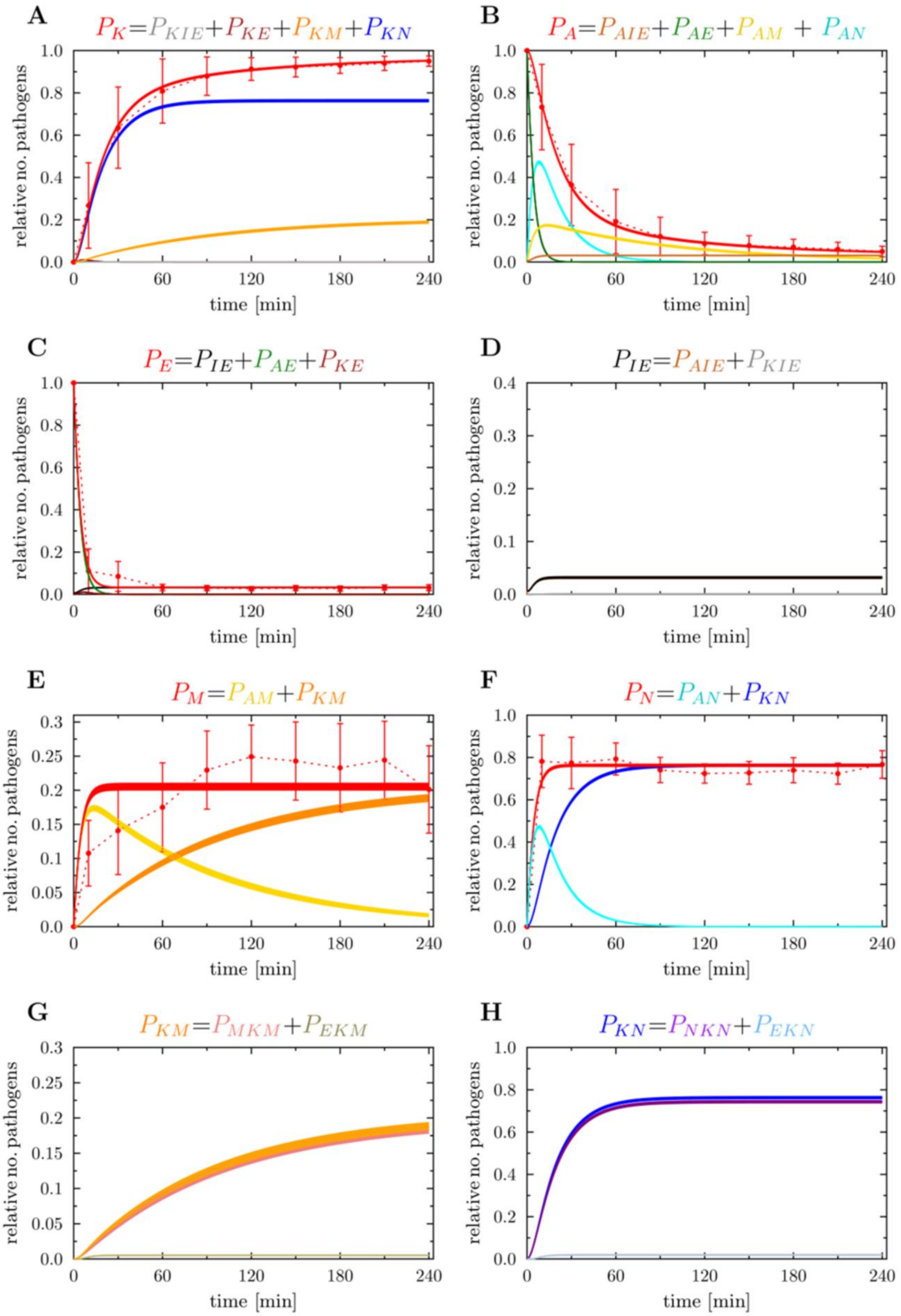

S23 Fig. Result of the PMNmed-alivePre-IE model simulation generated by optimized transition rates for a *S. aureus* infection. See caption of S19 Fig for the description of the subfigures.

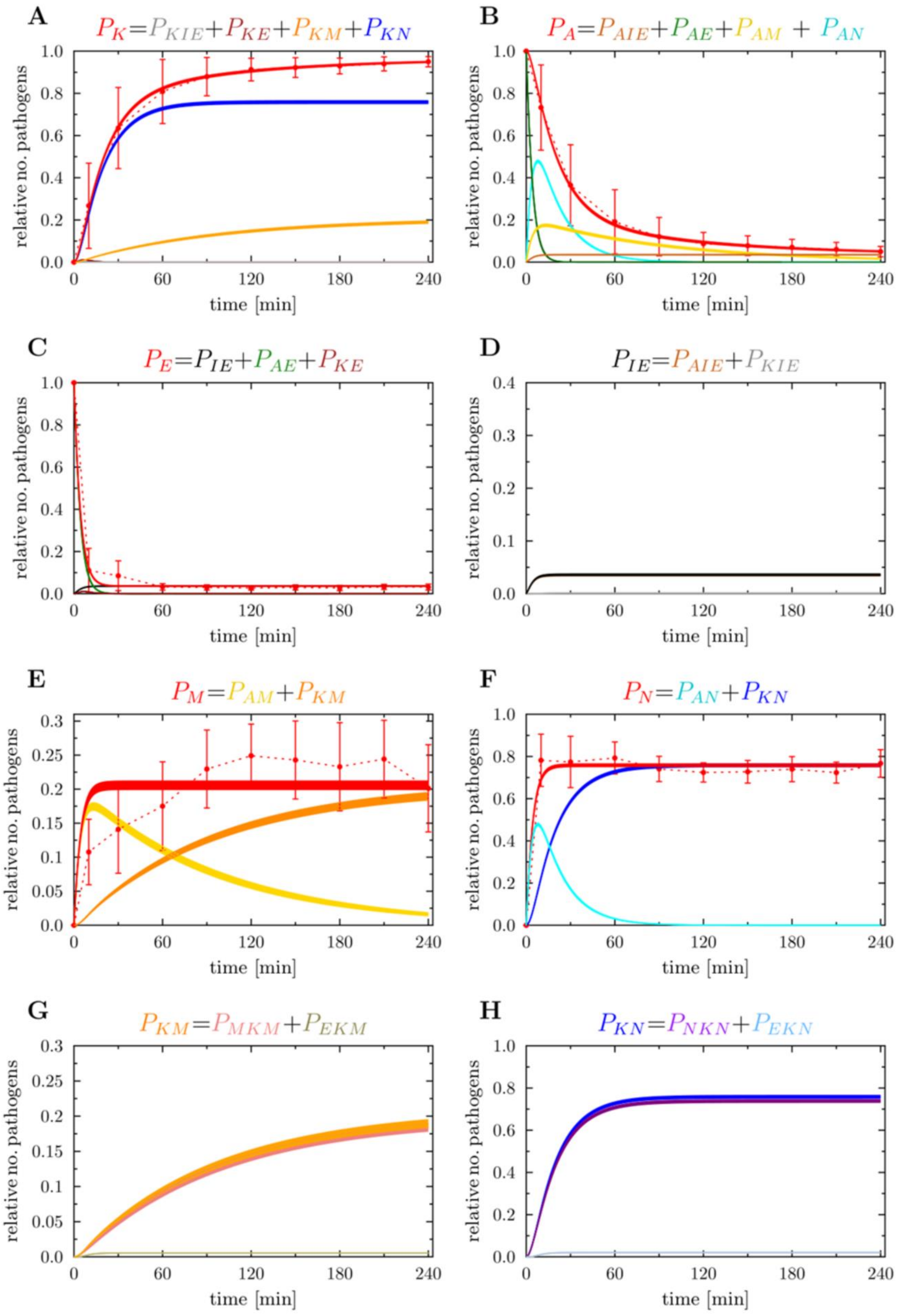

S24 Fig. Result of the spon-PMNmed-IE model simulation generated by optimized transition rates for a *S. aureus* infection. See caption of S19 Fig for the description of the subfigures.

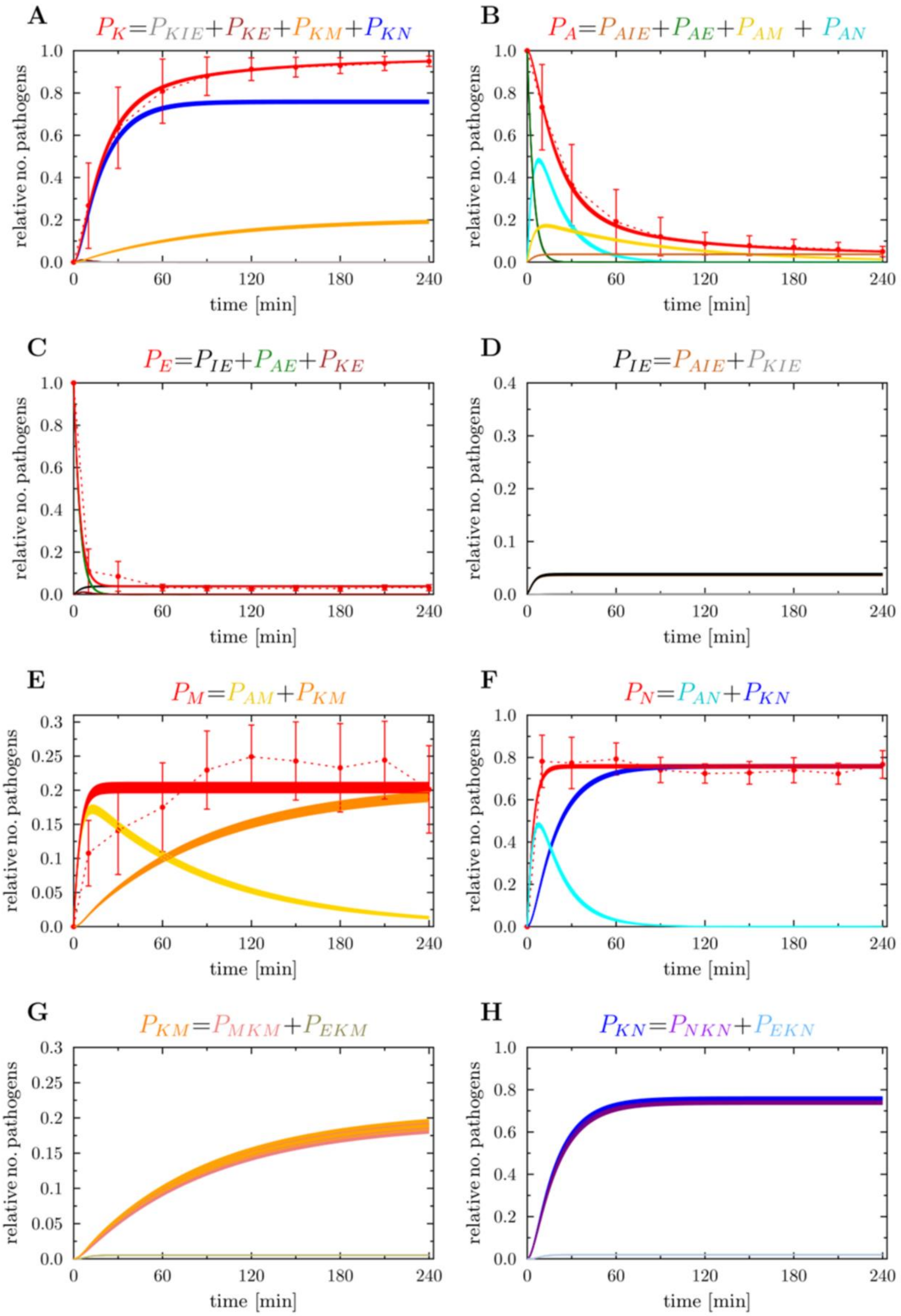

S25 Fig. Result of the spon-PMNmed-alivePre-IE model simulation generated by optimized transition rates for a *S. aureus* infection. See caption of S19 Fig for the description of the subfigures.

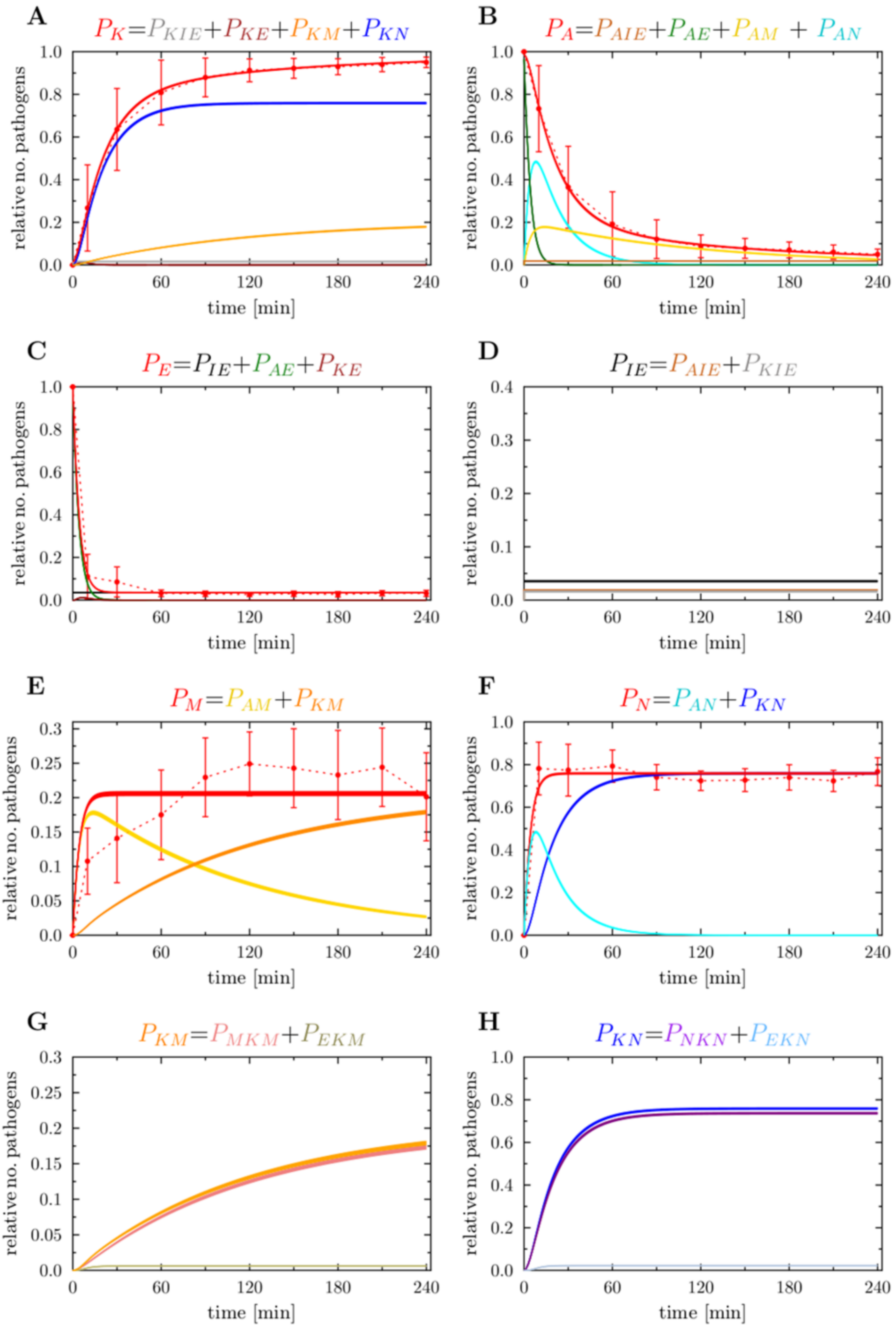

S26 Fig. Result of the pre-IE model simulation generated by optimized transition rates for a *S. aureus* infection. See caption of S19 Fig for the description of the subfigures.

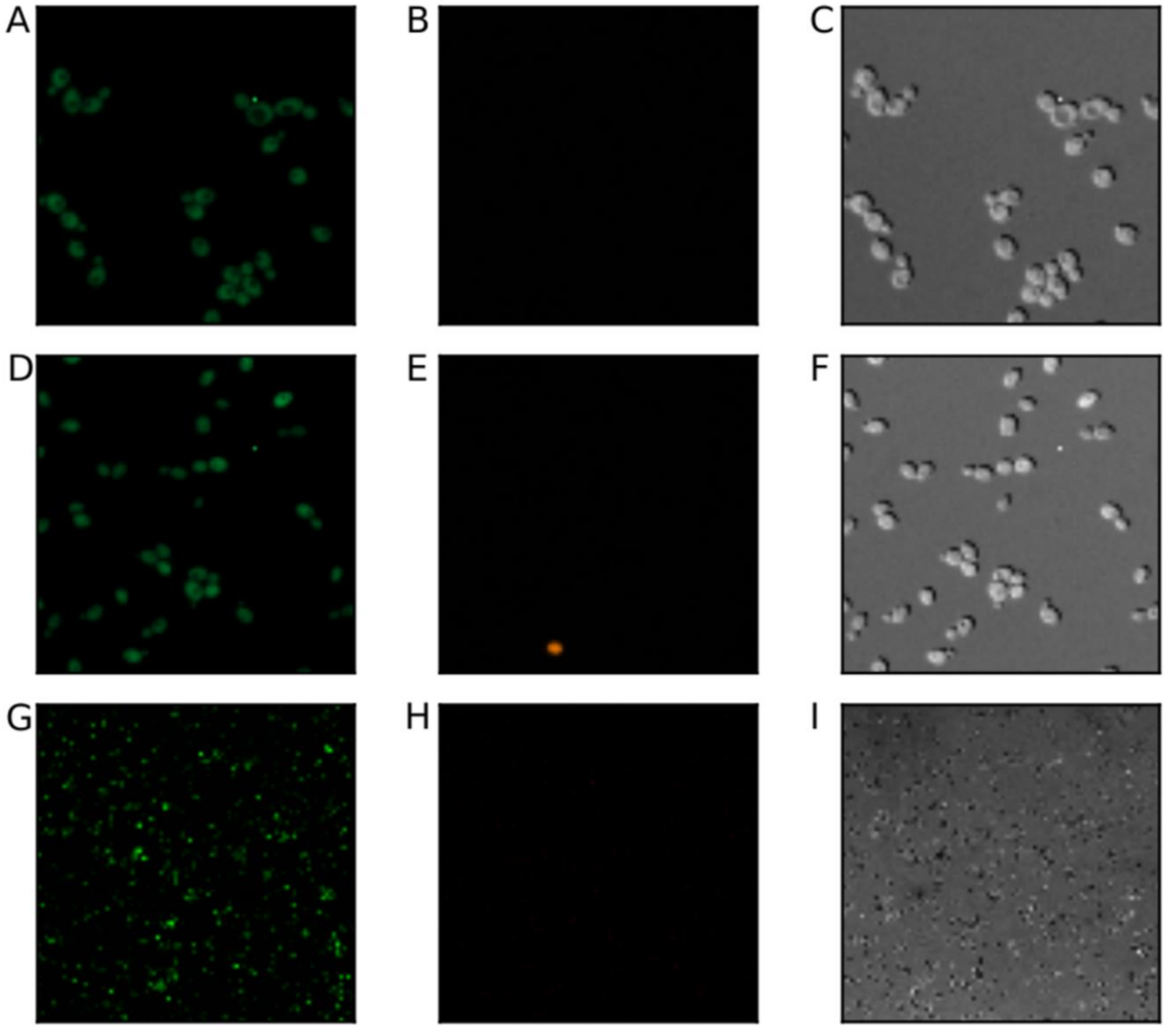

S27 Fig. Example microscopy images of the various pathogens as quantified by automated image analysis. The rows represent exemplary images for *C. albicans* (A-C) , *C. glabrata* (D-F) and *S. aureus* (G-I). The left column shows the images with GFP-labeled pathogens. Images with PH staining are given in the middle column. The right column shows transmitted light images for each pathogen.

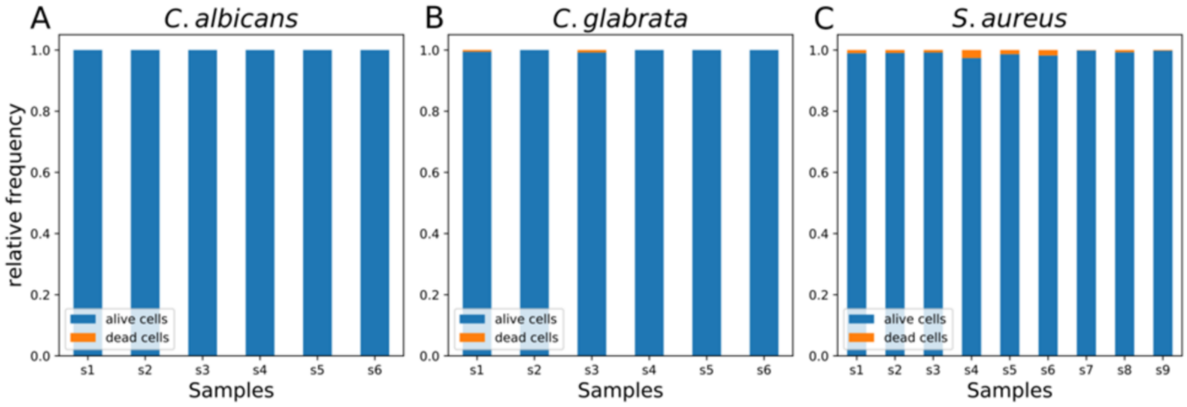

S28 Fig. Fraction of alive and dead *C. albicans* cells (A), *C. glabrata* cells (B) and *S. aureus* cells (C) as quantified by automated image analysis. While the *C. albicans* cells and *C. glabrata* cells were counted from six single microscopy images each, the *S. aureus* cells were counted from nine videos with ten time frames each. In (C) the bars and the error bars of each sample depict the mean and the standard deviation over the ten respective time frames.

Supporting Tables

C. albicans	mean x 10^-2 [min^-1] ± SD x 10^-2 [min^-1] (CV [%])						
rate	spon-IE model	PMNmed-IE model	alivePre-IE model	spon-alivePre-IE model	PMNmed-alivePre-IE model	spon-PMNmed-IE model	spon-PMNmed-alivePre-IE model
ϕ_N	2.97 $\pm$ 0.061 (2.1)	2.86 $\pm$ 0.054 (1.9)	3.3 $\pm$ 0.064 (1.9)	3.0 $\pm$ 0.04 (1.4)	3.1 $\pm$ 0.039 (1.2)	2.9 $\pm$ 0.057 (1.9)	2.9 $\pm$ 0.045 (1.5)
ϕ_M	1.23 $\pm$ 0.075 (6.1)	1.2 $\pm$ 0.13 (10.6)	1.7 $\pm$ 0.11 (6.5)	1.2 $\pm$ 0.096 (7.7)	1.4 $\pm$ 0.05 (3.5)	1.3 $\pm$ 0.006 (4.7)	1.2 $\pm$ 0.075 (6.1)
κ_N	5.3 $\pm$ 0.49 (9.2)	5.7 $\pm$ 0.97 (16.9)	10.6 $\pm$ 0.32 (3.0)	5.4 $\pm$ 0.47 (8.6)	9.1 $\pm$ 0.36 (4.0)	5.0 $\pm$ 0.44 (8.8)	5.5 $\pm$ 0.39 (7.1)
κ_M	2.1 $\pm$ 0.31 (14.6)	3.5 $\pm$ 0.42 (12.2)	2.8 $\pm$ 0.19 (6.8)	2.4 $\pm$ 0.27 (11.0)	3.3 $\pm$ 0.15 (4.6)	3.5 $\pm$ 0.44 (12.5)	3.0 $\pm$ 0.38 (13.0)
$\bar{\kappa}_{EK}$	21.8 $\pm$ 1.8 (8.0)	20.4 $\pm$ 3.33 (16.3)	10.7 $\pm$ 0.3 (2.8)	21.8 $\pm$ 2.72 (12.5)	11.5 $\pm$ 0.57 (5.0)	22.1 $\pm$ 2.89 (13.1)	18.9 $\pm$ 1.32 (7.0)
γ	2.13 $\pm$ 0.19 (8.8)	2.69 $\pm$ 0.34 (12.5)	4.9 $\pm$ 0.098 (2.0)	2.4 $\pm$ 0.42 (17.6)	3.5 $\pm$ 0.54 (15.7)	1.5 $\pm$ 1.0 (68.1)	1.4 $\pm$ 1.16 (85.7)
ρ	0.44 $\pm$ 0.021 (4.8)			0.4 $\pm$ 0.014 (3.4)		0.4 $\pm$ 0.02 (5.7)	0.3 $\pm$ 0.021 (6.5)
$\bar{\rho}$		10.8 $\pm$ 0.34 (7.1)			8.9 $\pm$ 0.38 (4.3)	5.3 $\pm$ 0.64 (12.2)	7.0 $\pm$ 1.24 (17.7)
γ_{IE}		3.0 $\pm$ 0.35 (11.6)			10.2 $\pm$ 0.11 (1.1)	14.1 $\pm$ 0.18 (1.3)	13.1 $\pm$ 0.15 (1.1)
population	mean [%] $\pm$ SD [%]						
P_{IE}			12.7 $\pm$ 0.4	0.2 $\pm$ 0.2	7.7 $\pm$ 0.4		0.02 $\pm$ 0.02

S1 Table. The estimated transition rates and alive immune-evasive population are shown for each model for infection scenario with *C. albicans*. The transition rates are given in the following order: the phagocytosis by PMN (ϕ_N) and monocytes (ϕ_M), intracellular killing by PMN (κ_N) and monocytes (κ_M) and the transition rates $\bar{\kappa}_{EK}$ and γ which determine give the extracellular killing. Furthermore, the spontaneous immune-evasion rate ρ and the transition rates $\bar{\rho}$ and γ_{IE}which, combined, give the PMN-mediated immune-evasion rate. The last entry is the estimated

population of alive immune-evasive cells which is initialized at the beginning of the simulation. The mean values and standard deviations (SD) of the transition rates and population were acquired through the global fitting procedure Metropolis Monte Carlo explained in the Methods section. The coefficient of variation (CV) refers to the ratio of the SD to the mean.

C. glabrata	mean x 10^-2^ [min^-1^] ± SD x 10^-2^ [min^-1^] (CV [%])						
**rate**	**spon-IE model**	**PMNmed-IE model**	**alivePre-IE model**	**spon-alivePre-IE model**	**PMNmed-alivePre-IE model**	**spon-PMNmed-IE model**	**spon-PMNmed-alivePre-IE model**
ϕ_N	10.0 ± 0.55 (5.5)	6.5 ± 0.49 (7.6)	8.0 ± 0.42 (5.2)	10.0 ± 0.55 (5.5)	10.1 ± 0.77 (7.6)	10.6 ± 0.58 (5.4)	10.5 ± 0.7 (6.6)
ϕ_M	14.3 ± 1.37 (9.6)	9.9 ± 0.64 (6.5)	11.9 ± 0.49 (4.1)	14.1 ± 1.28 (9.1)	14.2 ± 1.35 (9.5)	15.4 ± 1.53 (9.9)	15.1 ± 1.49 (9.9)
κ_N	4.9 ± 0.43 (8.7)	13.8 ± 2.52 (18.3)	10.3 ± 0.32 (3.1)	4.6 ± 0.34 (7.5)	6.1 ± 0.53 (8.6)	5.5 ± 0.37 (6.7)	4.7 ± 0.3 (6.4)
κ_M	2.2 ± 0.23 (10.6)	1.1 ± 0.073 (6.6)	2.4 ± 0.15 (6.4)	2.3 ± 0.33 (14.3)	2.0 ± 0.24 (12.4)	2.3 ± 0.33 (13.9)	2.7 ± 0.22 (8.1)
$\bar{\kappa}_{EK}$	47.5 ± 9.86 (20.8)	10.7 ± 1.61 (15.1)	11.7 ± 0.54 (4.6)	59.2 ± 10.43 (17.6)	29.7 ± 3.92 (13.2)	35.2 ± 5.83 (16.6)	51.5 ± 6.72 (13.1)
γ	3.1 ± 0.34 (10.9)	5.4 ± 1.25 (23.1)	5.2 ± 0.37 (7.0)	5.3 ± 0.81 (15.3)	5.8 ± 1.15 (20.0)	4.4 ± 2.95 (66.9)	3.8 ± 2.59 (68.8)
ρ	7.6 ± 0.047 (6.2)			0.8 ± 0.056 (7.3)		0.5 ± 0.089 (16.3)	0.6 ± 0.053 (8.9)
$\bar{\rho}$		7.3 ± 1.67 (22.8)			13.8 ± 2.6 (18.7)	9.0 ± 1.43 (15.9)	6.7 ± 0.98 (14.5)
γ_{IE}		7.9 ± 1.79 (22.7)			8.6 ± 0.66 (7.6)	18.2 ± 0.77 (4.2)	13.3 ± 0.53 (4.0)
**population**	**mean [%] ± SD [%]**						
P_{IE}			6.7 ± 0.5	0.1 ± 0.1	1.2 ± 1.0		0.02 ± 0.02

S2 Table. The estimated transition rates and alive immune-evasive population are shown for each model for infection scenario with *C. glabrata*. The transition rates are given in the following order: the phagocytosis by PMN (ϕ_N) and monocytes (ϕ_M), intracellular killing by PMN (κ_N) and monocytes (κ_M) and the transition rates $\bar{\kappa}_{EK}$ and γ which determine the extracellular killing. Furthermore, the spontaneous immune-evasion rate ρ and the transition rates $\bar{\rho}$ and γ_{IE}which, combined, give the PMN-mediated immune-evasion rate. The last entry is the estimated population of alive immune-evasive cells which is initialized at the beginning of the simulation.

The mean values and standard deviations (SD) of the transition rates and population were acquired through the global fitting procedure Metropolis Monte Carlo explained in the Methods section. The coefficient of variation (CV) refers to the ratio of the SD to the mean.

S. aureus	mean x 10^{-2} [min^{-1}] $\pm$ SD x 10^{-2} [min^{-1}] (CV [%])						
**rate**	**spon-IE model**	**PMNmed-IE model**	**alivePre-IE model**	**spon-alivePre-IE model**	**PMNmed-alivePre-IE model**	**spon-PMNmed-IE model**	**spon-PMNmed-alivePre-IE model**
$\boldsymbol{\phi_N}$	21.7 $\pm$ 0.52 (2.4)	19.6 $\pm$ 0.044 (0.2)	21.9 $\pm$ 0.37 (1.7)	21.2 $\pm$ 0.33 (1.6)	21.1 $\pm$ 0.44 (2.1)	21.7 $\pm$ 0.68 (2.8)	22.4 $\pm$ 0.61 (2.7)
$\boldsymbol{\phi_M}$	58.1 $\pm$ 1.37 (2.4)	52.7 $\pm$ 0.12 (3.0)	59.1 $\pm$ 1.14 (1.9)	57.6 $\pm$ 0.99 (1.7)	56.9 $\pm$ 1.19 (2.1)	59.0 $\pm$ 1.79 (3.0)	60.5 $\pm$ 2.0 (3.3)
$\boldsymbol{\kappa_N}$	6.1 $\pm$ 0.18 (3.0)	6.3 $\pm$ 0.031 (0.5)	6.2 $\pm$ 0.15 (2.4)	6.1 $\pm$ 0.047 (0.8)	6.2 $\pm$ 0.13 (2.1)	6.1 $\pm$ 0.13 (2.1)	6.1 $\pm$ 0.26 (4.3)
$\boldsymbol{\kappa_M}$	1.3 $\pm$ 0.048 (3.8)	0.1 $\pm$ 0.029 (3.0)	1.3 $\pm$ 0.055 (4.2)	1.3 $\pm$ 0.033 (2.6)	1.3 $\pm$ 0.068 (5.5)	1.3 $\pm$ 0.063 (5.0)	1.4 $\pm$ 0.058 (4.3)
$\boldsymbol{\bar{\kappa}_{EK}}$	12.0 $\pm$ 0.57 (4.7)	13.0 $\pm$ 1.03 (7.9)	12.2 $\pm$ 0.46 (3.7)	12.9 $\pm$ 1.1 (8.8)	11.4 $\pm$ 0.79 (6.9)	12.0 $\pm$ 0.58 (4.8)	12.8 $\pm$ 0.52 (4.1)
$\boldsymbol{\gamma}$	4.4 $\pm$ 0.31 (7.1)	3.5 $\pm$ 0.28 (8.2)	6.4 $\pm$ 0.24 (3.8)	3.8 $\pm$ 0.29 (7.9)	5.3 $\pm$ 0.27 (5.1)	4.0 $\pm$ 1.28 (31.7)	6.4 $\pm$ 1.48 (22.9)
$\boldsymbol{\rho}$	0.8 $\pm$ 0.064 (7.7)			0.8 $\pm$ 0.024 (3.1)		0.6 $\pm$ 0.034(5.9)	0.6 $\pm$ 0.028 (4.9)
$\boldsymbol{\bar{\rho}}$		5.1 $\pm$ 0.41 (8.1)			13.0 $\pm$ 0.73 (5.6)	7.6 $\pm$ 0.3 (3.9)	8.6 $\pm$ 0.71 (8.3)
$\boldsymbol{\gamma_{IE}}$		7.4 $\pm$ 0.26 (3.6)			10.6 $\pm$ 0.48 (4.5)	19.8 $\pm$ 0.15 (0.8)	15.9 $\pm$ 0.42 (2.7)
**population**	**Mean [%] $\pm$ SD [%]**						
$\boldsymbol{P_{IE}}$			3.6 $\pm$ 0.1	0.1 $\pm$ 0.05	0.6 $\pm$ 0.2		0.03 $\pm$ 0.03

S3 Table. The estimated transition rates and alive immune-evasive population are shown for each model for infection scenario with *S. aureus*. The transition rates are given in the following order: the phagocytosis by PMN (ϕ_N) and monocytes (ϕ_M), intracellular killing by PMN (κ_N) and monocytes (κ_M) and the transition rates $\bar{\kappa}_{EK}$ and γ which determine the extracellular killing. Furthermore, the spontaneous immune-evasion rate ρ and the transition rates $\bar{\rho}$ and γ_{IE}which, combined, give the PMN-mediated immune-evasion rate. The last entry is the estimated population of alive immune-evasive cells which is initialized at the beginning of the simulation. The mean values and standard deviations (SD) of the transition rates and population were

acquired through the global fitting procedure Metropolis Monte Carlo explained in the Methods section. The coefficient of variation (CV) refers to the ratio of the SD to the mean.

Algorithm-parameter	n	m	$dist_{min}$	$Area_{min}$	Rectangle-$width_{min}$	Rectangle-$width_{max}$	Rectangle-$length_{min}$	Rectangle-$length_{max}$	$Area_{threshold}$
C. albicans	38	12	1	70	12	75	39	74	534
**Killed *C. albicans***	38	12	1	70	12	76	38	75	480
C. glabrata	30	10	1	70	10	60	32	58	410
**Killed *C. glabrata***	24	8	1	70	8	50	26	50	150

S4 Table. The parameter used for the cluster splitting algorithm in which the search of the single cells is performed by scanning the convex edges of neighboring cells. All parameters were individually adapted to the different types of pathogens. The recommended settings from Brandes *et al.* [16] were used: n is the average single cell diameter and is used to scan the contours within a cluster and its concave regions. To determine the distance of the endpoints of a chord to the corresponding convexity point, m is set to a third of n. The minimum distance between a convex point and its corresponding chord is determined by $dist_{min}$, whose value depends on the depth of the convexity region within a cell cluster. $Area_{min}$ is the minimum number of pixels of a region, which is then segmented with the cluster splitting process. For the underlying images, a below-average size was specified to prevent a region from being lost. In order to find the convexity points mentioned above, the search rectangle grows in width and length from the minimum distance of the pixels to the corresponding maximum distance (Rectangle-$width_{min,}$ Rectangle-$width_{max,}$ Rectangle-$length_{min,}$ Rectangle-$length_{max}$). The $Area_{threshold}$ parameter is used as a post-processing step according to the cluster method in order not to include too small cells and spores in the number of all cells on an image.

transition rate	description	state transition
Φ_N	phagocytosis by neutrophils	$N_{i,j} + P_{AE} \rightarrow N_{i+1,j}$<br>$N_{i,j} + P_{KE} \rightarrow N_{i+1,j}$
Φ_M	phagocytosis by monocytes	$M_{i,j} + P_{AE} \rightarrow M_{i+1,j}$<br>$M_{i,j} + P_{KE} \rightarrow M_{i+1,j}$
κ_N	intracellular killing by neutrophils	$N_{i,j} \rightarrow N_{i-1,j+1}$
κ_M	intracellular killing by monocytes	$M_{i,j} \rightarrow M_{i-1,j+1}$
$\kappa_{EK}(t)$	extracellular killing by antimicrobial peptides released upon first-time PMN phagocytosis with decreasing activity<br><br>rate depends on the activity of antimicrobial peptides, characterized by the rate $\bar{\kappa}_{EK}$, and the decay of their antimicrobial activity, characterized by γ, as defined in Hünniger et al. (2014) (1) and Lehnert et al. (2015) (2)	$P_{AE} \rightarrow P_{KE}$
ρ	constant rate for the acquisition of immune evasion for spon-IE model	$P_{AE} \rightarrow P_{AIE}$<br>$P_{KE} \rightarrow P_{KIE}$
$\rho(t)$	time-dependent rate for the acquisition of immune evasion for PMNmed-IE model<br><br>rate depends on the activity of effector molecules, characterized by $\bar{\rho}$ and the decay of their activity as characterized by γ_{IE} (see Eq. 10 in the manuscript)	$P_{AE} \rightarrow P_{AIE}$<br>$P_{KE} \rightarrow P_{KIE}$

S5 Table. Transition rates of the state-based virtual infection models. For details see Methods section and Hünniger *et al.* [1] and Lehnert *et al.* [14].

Hünniger K*, Lehnert T*, Bieber K, Martin R, Figge MT[+], Kurzai O[+] (2014) A virtual infection model quantifies innate effector mechanisms and *Candida albicans* immune escape in human blood. *PLOS Comput Biol* 10(2), e1003479, */[+]authors contributed equally.

Lehnert T*, Timme S*, Pollmächer J, Hünniger K, Kurzai O, Figge MT (2015) Bottom-up modeling approach for the quantitative estimation of parameters in pathogen-host interactions. *Frontiers in Microbiology* 6(608), *authors contributed equally.
